# Quantitative analysis of differences in copy numbers using read depth obtained from PCR-enriched samples and controls

**DOI:** 10.1186/s12859-014-0428-5

**Published:** 2015-01-28

**Authors:** Frank Reinecke, Ravi Vijaya Satya, John DiCarlo

**Affiliations:** 1 Bioinformatics Assay Design & Analysis, QIAGEN GmbH, Max-Volmer-Straße 4, Hilden, 40724 Germany; 2 Bioinformatics Assay Design & Analysis, QIAGEN Sciences Inc., 6951 Executive Way, Frederick MD, 21703 USA

**Keywords:** Copy number variation, Amplicon sequencing, PCR enrichment, Next-generation-sequencing

## Abstract

**Background:**

Next-generation sequencing (NGS) is rapidly becoming common practice in clinical diagnostics and cancer research. In addition to the detection of single nucleotide variants (SNVs), information on copy number variants (CNVs) is of great interest. Several algorithms exist to detect CNVs by analyzing whole genome sequencing data or data from samples enriched by hybridization-capture. PCR-enriched amplicon-sequencing data have special characteristics that have been taken into account by only one publicly available algorithm so far.

**Results:**

We describe a new algorithm named quandico to detect copy number differences based on NGS data generated following PCR-enrichment. A weighted *t*-test statistic was applied to calculate probabilities (*p*-values) of copy number changes. We assessed the performance of the method using sequencing reads generated from reference DNA with known CNVs, and we were able to detect these variants with 98.6% sensitivity and 98.5% specificity which is significantly better than another recently described method for amplicon sequencing. The source code (R-package) of quandico is licensed under the GPLv3 and it is available at https://github.com/reineckef/quandico.

**Conclusion:**

We demonstrated that our new algorithm is suitable to call copy number changes using data from PCR-enriched samples with high sensitivity and specificity even for single copy differences.

**Electronic supplementary material:**

The online version of this article (doi:10.1186/s12859-014-0428-5) contains supplementary material, which is available to authorized users.

## Background

Cancer arises as a result of changes that have occurred in the genomes of cells. These somatic mutations may encompass several distinct classes of DNA sequence changes including substitutions of one base by another, insertions or deletions of small or large segments of DNA, rearrangements, and changes from normal copy number [1]. Copy number variations have been found to play an important role in cancer development [2] and are therefore of special interest to researchers in this field.

Various experimental methods can be used to detect CNV. Array comparative genomic hybridization (aCGH), is among the most commonly applied procedures [3,4]. Other array-based systems – although originally designed to genotype single nucleotide polymorphisms (SNP) – can also be used to provide information about potential copy number differences in addition to the genotypes [5].

Sequencing-based copy number analysis initially focused on paired-end-mapping [6,7]. These methods have been later refined to map actual breakpoints [8] and to use split-reads that span or cross breakpoints [9]. Frequencies obtained for the minor or *b*-allele may provide supporting evidence, and more complex methods integrate all available types of data [10,11].

Coverage or read depth has been used to detect CNVs in genome-scale datasets [12,13]. A few algorithms accept sequencing data generated following enrichment based on hybridization-capture [14,15]. Several review articles provide comprehensive lists and comparisons of available methods [16-18].

Multiplex PCR-based enrichment (MPE) focuses sequencing efforts on specific genes of interest or other regions, enabling deep sequencing and the identification of low allelic-fraction variants. However, the data obtained from this technology has special characteristics. Only a very small fraction of the genome is sequenced and these regions – typically exons of a limited number of genes – can be scattered all over the genome or focus on just a few loci. Each of these regions is addressed by a varying number of PCR amplicons that contribute to the total read depth.

A number of factors influence the observed read depth, including sequence variations which can lead to differing PCR enrichment efficiencies. Sequence reads that contain variants – especially insertions or deletions – may not map correctly to the reference genome and consequently lead to reduced observed read depth.

We have developed quandico to enable robust detection of CNVs by taking the special characteristics of MPE-derived data into account. Very recently, one approach named ONCOCNV has been published that is also tailored for PCR-based target-enrichment [19], and we have compared its performance with quandico using the same MPE-derived data in this paper.

## Methods

### Sequencing

To generate data from samples containing validated copy number variations across various regions of the genome, reference samples from the CNV Reference Panel CNVPANEL01 from the Coriell Institute for Medical Research (Camden, NJ, USA) were used. The cell line DNAs contained in this panel have been characterized as part of the Genetic Testing Reference Materials Coordination Program, using several commercial chromosomal microarray platforms and by several Cytogenetics experts from various institutes, and are recommended to be applied for developing and validating copy number assays [20].

A custom-made gene panel (CNA902Y) covering 35 genes in 40 clusters with 1783 individual amplicons (3566 primers) was designed to address the known variations in the reference material. The length of all amplicons was between 120 and 180 base pairs. Samples used with this panel were NA01201, NA01416, NA05067, NA09888, NA11672, NA12606, NA12878, NA13019, NA13783 and NA14164 together with two controls NA12878 and NA19219 for sequencing runs M62 and M63. Data from these samples are hereafter referred to as the training datasets.

A second custom panel (NGHS-991Y) was generated to validate the final algorithm developed using the CNA902Y results. This second validation panel contained 5512 primers targeting 56 genes in 60 clusters. Samples enriched using this panel were NA06226, NA09216, NA09367, NA10925, NA10985, NA11213, NA14485 and NA16595 together with NA12878 and NA19240 serving as controls for sequencing run M117. Please see Additional file 1 for sample details. We hereafter call data from run M117 the validation dataset.

Target enrichment was done as follows. 40 ng genomic DNA from each sample and control were amplified by PCR, purified and used for constructing barcoded Illumina DNA libraries following the QIAGEN GeneRead DNAseq Gene Panel Handbook. Libraries were quantified using QIAGEN’s GeneRead DNAseq Library Quant System. Illumina 150×2 paired-end sequencing was performed on a MiSeq instrument following manufacturer’s user manual (Illumina) to generate FASTQ files.

### Algorithm description

The CNV calling algorithm quandico was implemented as an R-package. However, the complete data processing pipeline, which extracts the raw counts from the reads and formats the output, was equally important. We used standard tools (if possible) and scripts written in Perl or Python for custom analyses and automation.

#### Sequence data processing

The sequence reads were aligned to the reference genome using BWA-MEM [21] and were then preprocessed according to the Broad best practice guidance except for duplicate removal (insertion/deletion realignment, base quality score recalibration, and base alignment quality scoring). Finally, the primer sequences were clipped away from the preprocessed reads by scanning the alignment around the original primer sites. On-target reads that contained the primer region in the alignment were trimmed and the event was counted. The resulting counts *C* for every primer *i* were written to a text file for processing with the quandico algorithm (see Additional file 2).

#### Normalization

The ratio of counts obtained for primer *i* between sample and the control was defined as *r*_*i*_=*l* *o* *g*_2_(*C*_*Si*_/*C*_*Ci*_). Each ratio was normalized using the global median *l* *o* *g*_2_-ratio $\tilde {r}$ to generate the normalized *l* *o* *g*_2_ ratio *x*_*i*_ (equation 1). This method assumes that the median value is a robust estimate for an unchanged locus, which is true if less than half of all loci (primers) are affected by either gain or loss.

(1) $$ x_{i} = \log_{2}\frac{C_{Si}}{C_{Ci}} - \tilde{r}   $$

#### Clustering

Algorithms that perform copy number estimation on genome-scale data usually include a final segmentation step that identifies regions of equal copy number. This approach is not feasible using MPE-data, which are very dense in targeted regions but have large gaps in between. Therefore, we assume that each of the relatively small targeted regions is of homogeneous copy number and that the CNV-causing events mainly occur in the remaining much larger fraction of the genome. We cluster read count data by position and do not provide any segmentation analysis of data assigned to the same cluster.

The normalized *l* *o* *g*_2_-ratios (*x*_*i*_) were grouped into clusters (*X*) based on their coordinates using a custom Perl script. This clustering ensures a balance between granularity and predictive power, so that larger genes with many amplicons in distant exons were split into more than one cluster and other smaller but neighboring or overlapping genes formed a single cluster.

First, we assigned all amplicons from the same chromosome to one cluster. Subsequently, the largest gap between any two amplicons in the same cluster was taken to split that region into two clusters if both newly formed clusters contained at least ten amplicons or the gap was larger than 250 kbp. Clusters with less than 100 amplicons were only separated at large gaps of at least 100 kbp.

#### Outlier removal

Variance can be caused not only by statistical scatter and experimental measurement variation, but also by factors that create true outliers. The real sampling process that we rely on here is the elongation of a specific oligonucleotide hybridized to the target DNA during PCR enrichment. Biochemically, this process can be impaired by mutations of the primer binding site. Furthermore, it is known that PCR efficiencies are highly variable among PCR reactions, and that the main factor which defines the efficiency of a reaction is the amplicon sequence [22]. Natural variations, expected mainly for non-matched controls, or somatic mutations if matched controls are used, can account for amplicon variability. Larger deletions or insertions of regions could cause additional problems in mapping the sequence reads correctly.

To reduce noises originating from these true outliers, a Shapiro and Wilk’s *W* test for normality [23] was performed using the *l* *o* *g*_2_ ratios of each cluster separately. If this test rejected normal distribution (*p*-value ≤0.05), the element with the largest difference from the weighted mean *μ* ^∗^ (equation 2) was removed, and the test was repeated until all remaining measurements were normally distributed. At most one third of data points was removed this way, resulting in the subset *X* ^′^ out of *X*. The numbers of total primers per cluster (*n*=|*X*|) and the effective subset used (*n* ^′^=|*X* ^′^|) were reported.

#### Weighted mean and variance

The resulting set of values for every cluster *X* ^′^ followed a distribution around *μ* with a standard deviation *σ*. To account for higher predictive power of ratios derived from higher original read counts, every value was weighted. To calculate the weighted mean *μ* ^∗^ (equation 2), the sum of original read counts for every comparison was used as the weight (*w*_*i*_=*C*_*Si*_+*C*_*Ci*_, and $W=\sum _{i \in X'} w_{i}$). Because the individual counts of *C*_*Si*_ and *C*_*Ci*_ can be regarded as repetitive sampling from the same distribution (the extension event at primer *i*), the unbiased weighted variance (${\sigma _{w}^{2}}$) is given by equation 3. The effective sampling size (*n* ^∗^) is influenced by these weights and is defined by equation 4, and the standard error (SE) is given by equation 5, where *z* is the quantile function of the *t* distribution and *α* corresponds to the significance level (0.05 was used here).

(2) $$\begin{array}{@{}rcl@{}} \mu^{*} &=& \frac{1}{W} \sum_{i \in X'} w_{i} x_{i}  \end{array} $$

(3) $$\begin{array}{@{}rcl@{}} {\sigma_{w}^{2}} &=& \frac{1}{W-1} \sum_{i \in X'} w_{i} \left(x_{i} - \mu^{*}\right)^{2} \end{array} $$

(4) $$\begin{array}{@{}rcl@{}} n^{*} &=& \left(\sum_{i \in X'}{w_{i}}\right)^{2}\left/\sum_{i \in X'}{{w_{i}^{2}}}\right. \end{array} $$

(5) $$\begin{array}{@{}rcl@{}} \mathrm{SE_{\mu^{*}}} &=& z\left(1-\frac{\alpha}{2}, n^{*}-1\right) \frac{\sigma_{w}}{\sqrt{n^{*}}}  \end{array} $$

The weighted mean *μ* ^∗^ was subsequently used to calculate the copy number of the sample (*N*_*S*_) which requires knowledge of the copy number of the control (*N*_*C*_) in the same region (which is usually 2 for diploid genomes, but different for chromosomes X and Y depending on gender). The standard error SE was used to calculate the lower and upper bounds of the confidence interval (*N*_*S*(min,max)_) using equation 6.

(6) $$ \begin{aligned} N_{S} &= N_{C} \times 2^{\mu^{*}} \\ N_{S(\text{min})} &= N_{C} \times 2^{\left(\mu^{*} - \mathrm{SE_{\mu^{*}}}\right)} \\ N_{S(\text{max})} &= N_{C} \times 2^{\left(\mu^{*} + \mathrm{SE_{\mu^{*}}}\right)}  \end{aligned}  $$

#### Hypothesis testing

If there is no difference in copy number between sample and control, the hypothesis to test against is *μ* ^∗^=*μ*_0_, and the expected *l* *o* *g*_2_ value for unchanged loci (where *C*_*S*_=*C*_*C*_) is *μ*_0_= log2(*C*_*S*_/*C*_*C*_)= log2(1)=0. The *t*-test was performed and the corresponding *p*-value was obtained via the t-distribution function Pr (equation 7).

(7) $$ \begin{aligned} t & = \sqrt{n^{*}} \frac{\mu^{*}-\mu_{0}}{\sigma_{w}} \\ p & = 2 \times \text{Pr}\left(-|t|, n^{*}\right)  \end{aligned}  $$

Normal distribution of data is a prerequisite for applying a *t*-test. A reviewer pointed out that, in the presence of two or more subclones, if we assume their *l* *o* *g*_2_ ratios are approximately normal, the derived *l* *o* *g*_2_-ratios from this clonal composition would follow a mixture of two Gaussian distributions, which is not the same as a normal distribution. This is correct for methods that are able to measure individual cells independently, but would not apply if the DNA of a sample that is used as template to perform the PCR enrichment in our experiment is isolated from many cells at once, and these cells could potentially be populations of subclones. Details are provided in Additional file 3.

#### Dispersion correction

**Figure 1:**
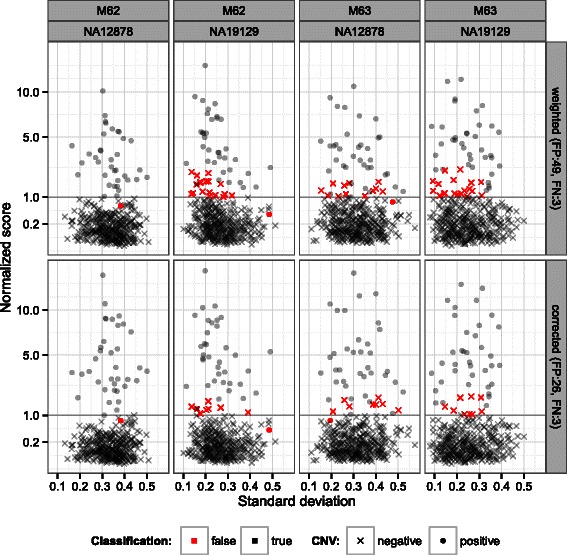
**Dispersion correction.** Illustration of the dispersion correction effect by *ϕ*. First row: before correction, second row: after correction. Calls for the sequencing data sets (M62 and M63) and both control samples (NA12898, NA19129) are plotted separately (in columns). A CNV is called if the determined *Q* score is higher than the threshold (normalized to 1 in this diagram). False classifications (FN: false negative, FP: false positive) are shown in red. Loci with known CNVs in the sample are shown as dots and loci with normal copy number are plotted as crosses.

The factor $\phi =\sqrt {\sigma _{w}}\times (1+|\mu ^{*}|)$ in equation 8 was introduced to correct false classifications. Without correction, clusters with expected negative result and with low dispersion showed over-estimated *p*-values (Figure 1, top row: × colored in red, false positives). On the other hand, *p*-values for clusters with known CNVs were under-estimated if the dispersion was high (∙, also red, false negatives). The *p*-values alone did not allow optimal discrimination. After correction with the factor *ϕ*, the linear discrimination yielded both fewer false positives and fewer false negatives (bottom row in Figure 1).

The magnitude of the observed difference |*μ* ^∗^| is incorporated into factor *ϕ* (hence the final score *Q*) so that no additional threshold for the *l* *o* *g*_2_ scaled values needs to be applied. The data in Figure 1 is shown in groups of runs and controls (columns) to demonstrate the general truth of this finding.

(8) $$\begin{array}{@{}rcl@{}} Q & =& -10 \times \log_{10}(p) \times \phi  \end{array} $$

(9) $$\begin{array}{@{}rcl@{}} P & =& -10 \times \ln(\mathrm{SE_{\mu^{*}}})  \end{array} $$

The copy number for every cluster of the sample was estimated to be *N*_*S*_. The score *Q* was used to set a threshold (*Q*≥50) to identify clusters with significant copy number changes and values *P* correlated with the precision of the copy number estimate (equation 9).

*Q* was used to populate the filter-column of the variant call format (VCF) output file, because it is the discriminating score that indicated copy number changes. Additionally calculated copy number values *N*_*S*_ (confidence range *N*_*S*(min)_ – *N*_*S*(max)_) and the call precision score *P* were added to the optional annotation tags for every entry.

### Algorithm comparison

We used sequencing data from runs M62 and M63 to compare the performances of quandico and ONCOCNV, because each run contains two control samples (NA12878 and NA19129) and ONCOCNV requires a set of at least four control samples to generate a baseline, and the independent validation dataset only contains two controls. All test samples have been enriched with the panel CNA902Y, but we excluded regions on chromosome X from the analysis, because it was not possible to specify gender per sample in ONCOCNV. In addition, we excluded sample NA09888 from run M63 because ONCOCNV reported gains for most regions except for the region 8q23.1q24.12, which is in fact a true deletion (see Additional file 4). We named this subset of the training dataset the comparison dataset.

#### ONCOCNV

We downloaded version 5.5 of the package from https://oncocnv.curie.fr/. FASTQ files were processed as described above, and the BAM files after primer trimming were used as input for ONCOCNV together with a file containing amplicon coordinates of panel CNA902Y. The segmentation step was set to use the cghseg method, because it mainly generated one cluster per gene. For reported breakpoints, every segment was counted separately.

#### Quandico

To create the same number of target regions, quandico was run with the ‘–no-cluster‘ option, which uses gene annotation for cluster generation. Instead of using a single control for every comparison, we have combined all read counts obtained from the four controls into one synthetic control, by averaging the primer-trimming-counts of the same four control BAM files used to generate the baseline for ONCOCNV.

#### Assessment

The output files of both packages are provided in Additional file 4. For ONCOCNV, we used the summary output files. Every reported copy number of 2 was regarded as *negative* and all different from 2 were called *positive*. Events were counted as *true positive* if the estimated copy number gain, copy number loss or no change agrees with the annotation provided by the Coriell Institute (but not requiring an exact match of copy numbers). Otherwise, events were counted as *false positive*. In other words, if the reported copy number was 3.5, but the sample actually had three copies of the region in question, the event was still counted as *true positive*.

Results obtained by quandico were classified using the assigned score *Q* (from the VCF) directly. Every cluster that exceeded the default score-threshold (50) was counted as *positive* (*negative* otherwise), regardless of the reported copy number. Comparing to the annotation provided by the Coriell Institute, the event was counted as *true positive* or *false positive* as described above for ONCOCNV.

### Ethical approval

The condition for use of NIGMS Repository Samples are governed by the Coriell Institutional Review Board (IRB) in accordance with the U.S. Department of Health and Human Services (DHHS) regulations (45 CFR Part 46). The Research Intent was approved by Coriell IRB before we received the samples. This study was conducted in compliance with the regulations for the protection of human subjects issued by the Office for Human Research Protections (Washington, DC) of DHHS.

## Results and discussion

We assessed the performance of quandico using sequencing reads generated from previously characterized samples, and we investigated the influence of different algorithm refinements.

Ten samples and two controls were used to generate the training datasets, which consist of two sets of FASTQ files with different amounts of total sequence data. The first set had a median read depth of 1500–2000 × (high coverage, M62) and the second set had a median read depth of 500–1000 × (medium coverage, M63).

All 1600 individual copy number calls (40 clusters, 10 samples, two different controls and two independent sequencing runs) were routinely investigated during refinement of the algorithm. The count data are included in Additional file 2.

### Algorithm refinement

**Figure 2:**
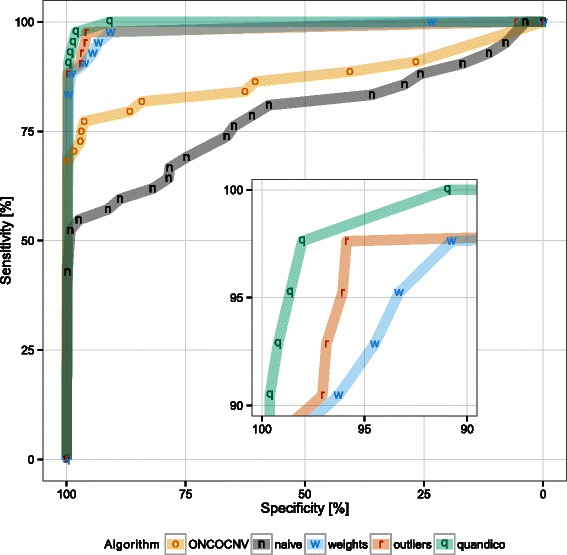
**ROC curve.** Receiver operator characteristic curves for ONCOCNV (o) and our own development using the comparison dataset (Table 2): Plain *t*-test statistics without corrections (naive, n) compared to the performance achieved by using weights (w) or removing outliers (r). The final algorithm quandico (q) includes both of these steps together with an additional dispersion balancing. Symbols are plotted for local maxima. The inset shows a magnification of the region above 90% specificity and sensitivity.

**Figure 3:**
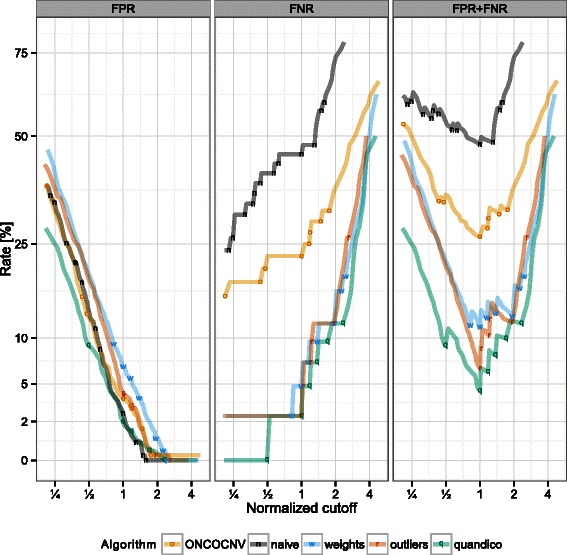
**False positive/negative rates.** False positive rate (FPR) and false negative rates (FNR) observed on the comparison dataset. The optimal threshold for every algorithm was determined by selecting the value that generated the minimal sum of FPR and FNR. The scores for every individual algorithm (x-axis) were then divided by the identified threshold (normalized to 1) for comparison. For algorithm details, see legend of Figure 2.

Initially, a *t*-test statistic using *l* *o* *g*_2_ ratios of all primer sites in a certain cluster was used, but classification performance based on the obtained *p*-values alone was not satisfactory (Figure 2, naive). Removal of outliers had a significant effect, mainly on the false negative rate. A similar effect can be seen by calculating weighted means instead of simple averages per cluster (Figure 3, outliers and weighted).

Investigation of the score distribution and analysis of the remaining false classifications led to an additional step that we call dispersion correction. Clusters with apparently very low dispersion were prone to be assigned with significant *p*-values leading to false positive calls. On the other hand, clusters with a true copy number change but high dispersion rarely reached the significance threshold. In other words, correct classification was shown to be dependent on both the *p*-value and the standard error. To correct for this effect, the Phred-scaled *p*-value was multiplied by the square root of the standard error (equation 8) which further reduced both false positives (associated with low dispersion) and false negatives (associated with high dispersion, Figure 1).

Finally, a cutoff threshold for *Q* (equation 8) of 50 provided the best balance of sensitivity (98.0%) and specificity (98.2%, Table 1).

**Table 1:** **Performance metrics**

**Dataset**	**TP**	**TN**	**FP**	**FN**	**TPR**	**TNR**
Training	145	1426	26	3	0.980	0.982
Validation	72	878	10	0	1.000	0.989
Combined	217	2304	36	3	0.986	0.985

Summary of calls made using the training and validation datasets.

The score *P* (equation 9) was a useful indicator for the precision of the assigned copy number (see Additional file 5). Assigned copy numbers with *P* above 20 generally differ by less than 20 percent from the real copy number. Assigned copy numbers with *P* greater than 30 roughly show a maximal difference of 10 percent from the true copy number.

### Validation experiment

To validate the final algorithm, eight different samples from the CNVPANEL01 were selected for sequencing with the independent panel NGHS-991Y together with two control samples to a median coverage of 820. This validation set (new samples, different primer pool) was created after all algorithmic steps and parameters for quandico had been fixed. Detailed information on the reference samples and their validated CNV regions can be found in Additional file 1. The performance of the copy number assignments from the training and the validation datasets are summarized in Table 1. See Additional file 6 for a graphical representation. Applying methods and thresholds developed on the training dataset resulted in 72 true positives, 10 false positives, 878 true negatives and no false negatives from the validation dataset. These results corresponded to a sensitivity of 100%, a specificity of 98.9%, and a false-positive rate of 1.8% (Table 1). The performance on the validation set is even better than on the training set, so that over-fitting to a certain dataset can be rejected.

### Comparison

A summary of results comparing quandico and ONCOCNV is given in Table 2. quandico matched ONCOCNV regarding specificity and clearly outperformed it in terms of sensitivity, where ONCOCNV failed to detect 20 percent of all CNVs – even after removal of one sample (NA09888) that ONCOCNV obviously struggled with. The overall higher number of results for ONCOCNV is due to breakpoints which ONCOCNV reported inside the genes sequenced. No target region was actually spanning a breakpoint, meaning that all reported breakpoints are false positive, and were automatically counted as such.

**Table 2:** **Algorithm comparison using a subset of samples with four controls**

**Algorithm**	**TP**	**TN**	**FP**	**FN**	**Total**	**TPR**	**TNR**
quandico	41	491	18	1	551	0.976	0.965
ONCOCNV	35	503	10	9	557	0.795	0.981

The fact that the comparison with ONCOCNV is done on the same data that has been used to develop quandico, is not ideal because algorithmic parameters – especially the cutoff threshold – have been adjusted to achieve maximal performance. To overcome this problem, we assess the discriminative power of the generated scores *Q* (quandico) or *p*-values (ONCOCNV) with variable threshold settings and calculate the respective receiver operating characteristic (ROC) curves using the pROC package [24]. The fraction of true positives versus the fraction of false positives at various threshold settings is shown (Figure 2). To compare algorithms, we have normalized the thresholds resulting in the best performance of each algorithm (smallest sum of false positive rate and false negative rate) to 1 in Figure 3. The plots show that ONCOCNV’s sensitivity is always lower than quandico’s at the same level of specificity.

Our interpretation of the observed performance difference is that the more general approach implemented by quandico is more effective in selecting representative data for each target region. Outliers and/or very low read depth can be caused by a multitude of reasons. ONCOCNV aims to detect and correct for specific causes of bias, namely GC-content, amplicon length and library size. Instead, quandico removes outliers and weighs amplicons without knowing the reason leading to low read depth or outliers. The benefit of the correction steps implemented in ONCOCNV is obvious compared to the naive approach that does not include any corrective methods (Figures 2 and 3). On the other hand, the general reduction to reliable data and the weighting by total read depth as implemented in quandico is more robust and effective than correcting for some selective causes of bias.

## Conclusions

With quandico, we designed a new algorithm based on standard statistical methods, that is able to reliably detect copy number variations in sequencing data generated following PCR-enrichment of target regions with sensitivity and specificity of more than 98% (Table 1). Although the algorithm was originally designed to use data from matched samples and controls, where both DNAs are prepared from the same individual (e.g. tumor and a normal tissue), the performance achieved with non-matched controls is still very good when compared to other published methods using NGS read count data (see Table three in [18]), including the recently published ONCOCNV.

However, there are some limitations. First, the presented approach does not include any sample-purity estimation or breakpoint detection (segmentation) step. If a CNV-generating event occurred inside a cluster, the result will be inaccurate. Secondly, clusters must have at least ten normally distributed primer counts to qualify for copy number calling. Thirdly, the use of identical PCR primer pools (panels) to enrich regions from sample and control is mandatory. We processed samples and controls in parallel to minimize handling bias and also recommend this practice to achieve the best results. Lastly, using sample and control DNA of similar quality and quantity in PCR enrichment is also considered as important to achieve a good result.

## Additional files

Additional file 1
**Sample, panel and sequencing details.** This spreadsheet (xlsx) contains details on the samples selected for sequencing, panel details such as cluster coordinates and primers per individual cluster, as well as sequence read counts obtained for the three sequencing runs.

Additional file 2
**Primer read depth raw data.** This archive (zip) contains one tab-separated text file (tsv) per sequencing run and sample. Each file name is composed of the run id and sample name. The column definitions are: chromosome, position, direction, reference base, cluster name, depth and original gene name.

Additional file 3
**Normal distribution of mixed subclones.** This document (pdf) contains a brief explanation why *l* *o* *g*_2_ ratios follow normal distribution even if the sample consists of subclones with differing copy number.

Additional file 4
**Comparison with ONCOCNV.** This archive (zip) contains all output-files generated for the comparison of quandico and ONCOCNV.

Additional file 5
**Correlation of expected and estimated copy number.** Graph (pdf) of the score *P* (derived from the observed standard error, see equation 9) plotted against the actual precision of the copy number assignment. Deviation is calculated as (*N*_called_−*N*_expected_)/*N*_called_. The dashed lines correspond to a linear increase of precision (1 percentage point per increase in score). Different expected copy numbers are shown in three rows (top: <2, center: =2, bottom >2).

Additional file 6
**Summary of all calls.** Graphical representation (pdf) of all copy number calls done with the final algorithm. Page 1: CNA902Y with 1600 calls (40 clusters, ten samples, two controls, two runs). Each analysis is based on a comparison of a samples (column) with a control (row) of a certain sequencing run (row). Page 2: Set NGHS-991Y with 960 calls (60 clusters, eight samples, two controls). Regions with no expected CNV are shown as crosses (×) while expected differences are shown as circles (∙). The expected copy numbers are depicted by different colors.
