# Supplementary material for: Quantitative analysis of differences in copy numbers using read depth obtained from PCR-enriched samples and controls
Source: BMC Bioinformatics. 2015 Jan 28;16:17. doi: 10.1186/s12859-014-0428-5 (PMC4384318; doi:10.1186/s12859-014-0428-5)
Supplement: Additional file 3 — Normal distribution of mixed subclones. This document (pdf) contains a brief explanation why l o g 2 ratios follow normal distribution even if the sample consists of subclones with differing copy number. [file 12859_2014_428_MOESM3_ESM.pdf]

Additional file 3: Normal distribution of mixed subclones

Are log_2-ratios of read counts (sample/control) obtained from a subclonal mixture normally distributed?

Our algorithm is designed to analyze copy number changes in samples enriched using PCR panels that are mainly developed to cover genes relevant in cancer. Cancer cells are nearly always intermixed with an unknown fraction of normal cells (impurity) and/or the cancer cell population may be heterogeneous, perhaps due to subclonal evolution.

Methods that are able to measure individual cells independently, including Fluorescence-activated cell sorting (FACS), Fluorescence in situ hybridization (FISH) and single-cell PCR or even single-cell sequencing, will generate signals from all distinct distributions (*here:* the subclones present in the sample).

However, the DNA of a sample that is used as template to perform the PCR enrichment in our experiment is isolated from many cells at once. The origin (the cell the DNA was isolated from) will be lost during this process. The DNA template is therefore a mixture from all cells (subclones) present in the sample. All signals obtained from this mixture should follow a uniform normal distribution of the situation (*here*: chromosome counts) in this mixture, and not a mixture of the original distributions (e.g. subclones). Please see **Figure 1** (next page) for a visual example.

The t-statistics that we apply compare the observed distribution with the expected distribution (-0.51 versus $log_2(1) = 0$ in the example presented), and therefore we think this method can readily be applied to detect CNA (e.g. a somatic copy number changes in a cancer biopsy) in mixtures of cells, including subclones.

Additional file 3: Normal distribution of mixed subclones

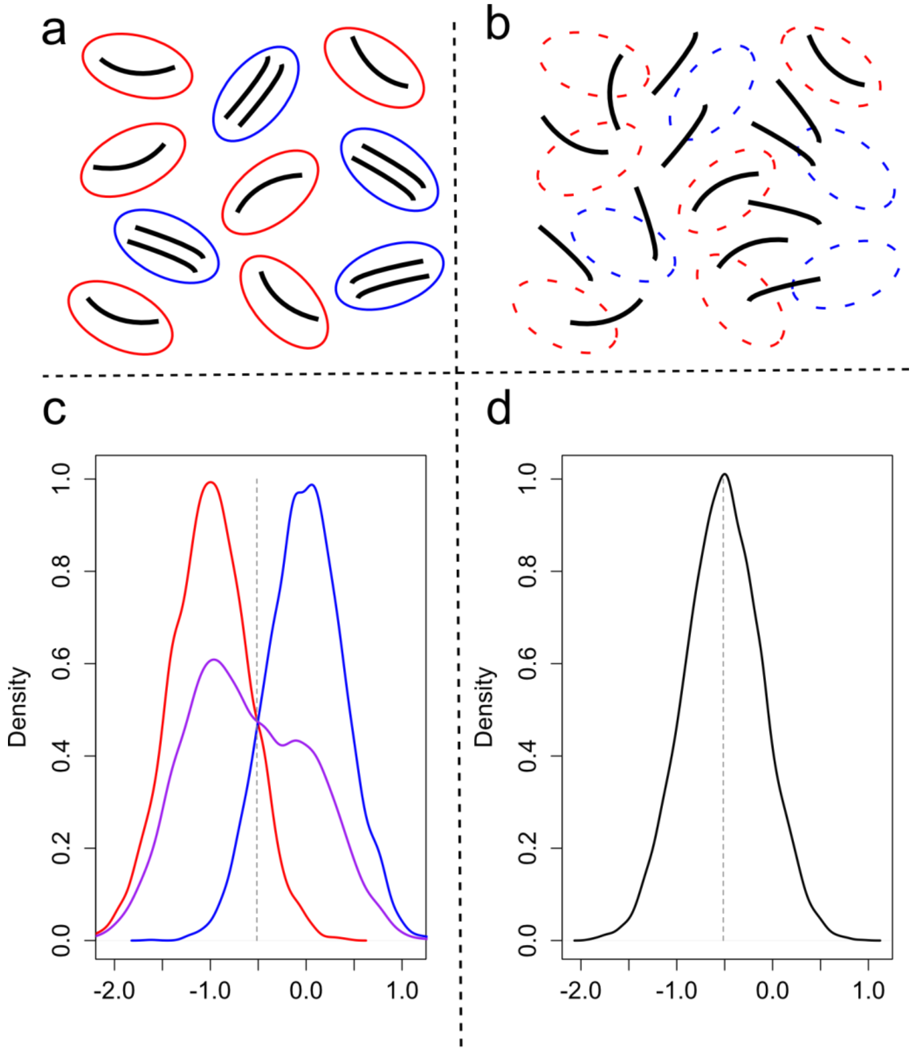

Figure 1: Visualization of the effect of different sample preparation methods on the expected signal distribution. A subset of an imaginary sample containing 60% tumor cells (red circles) with a loss of an autosomal chromosome (depicted as solid black line) and 40% normal cells (blue circles) still containing two copies of the same chromosome is shown (**a**) and compared with a completely normal population of cells with two copies of the respective chromosome in every cell (not drawn). Methods that are able to analyze this mixture using <u>intact</u> cells (**a** and **c**) or <u>isolated DNA</u> from such a mixture (**b** and **d**) lead to different signal distributions. Methods that are able to generate one signal per individual cell (**c**, see text for example methods) will result in a mixture of <u>normal</u> signals (blue, $\log_2(2/2)=0$) and <u>weak</u> signals (red, $\log_2(1/2)=-1$) from the two populations. Thus, the total density of all signals will be bipartite (purple). The process of DNA isolation from the mixed population (**b**) leads to a homogenous mixture of all chromosomes present in both populations (*here*: 14 from 10 cells instead of the expected 20 for normal cells), and the distribution of signals generated from this DNA mixture will follow normal distribution around the ratio of $\log_2(14/20) = -0.51$ (**d**, dashed line).
