# Supplementary material for: Quantitative analysis of differences in copy numbers using read depth obtained from PCR-enriched samples and controls
Source: BMC Bioinformatics. 2015 Jan 28;16:17. doi: 10.1186/s12859-014-0428-5 (PMC4384318; doi:10.1186/s12859-014-0428-5)
Supplement: Additional file 4 — Comparison with ONCOCNV. This archive (zip) contains all output-files generated for the comparison of quandico and ONCOCNV. [file 12859_2014_428_MOESM4_ESM.zip › quandico/M62_S2_NA01416_M62M63_SC.cov.pdf]

QIAGEN GeneRead Copy Number Analysis

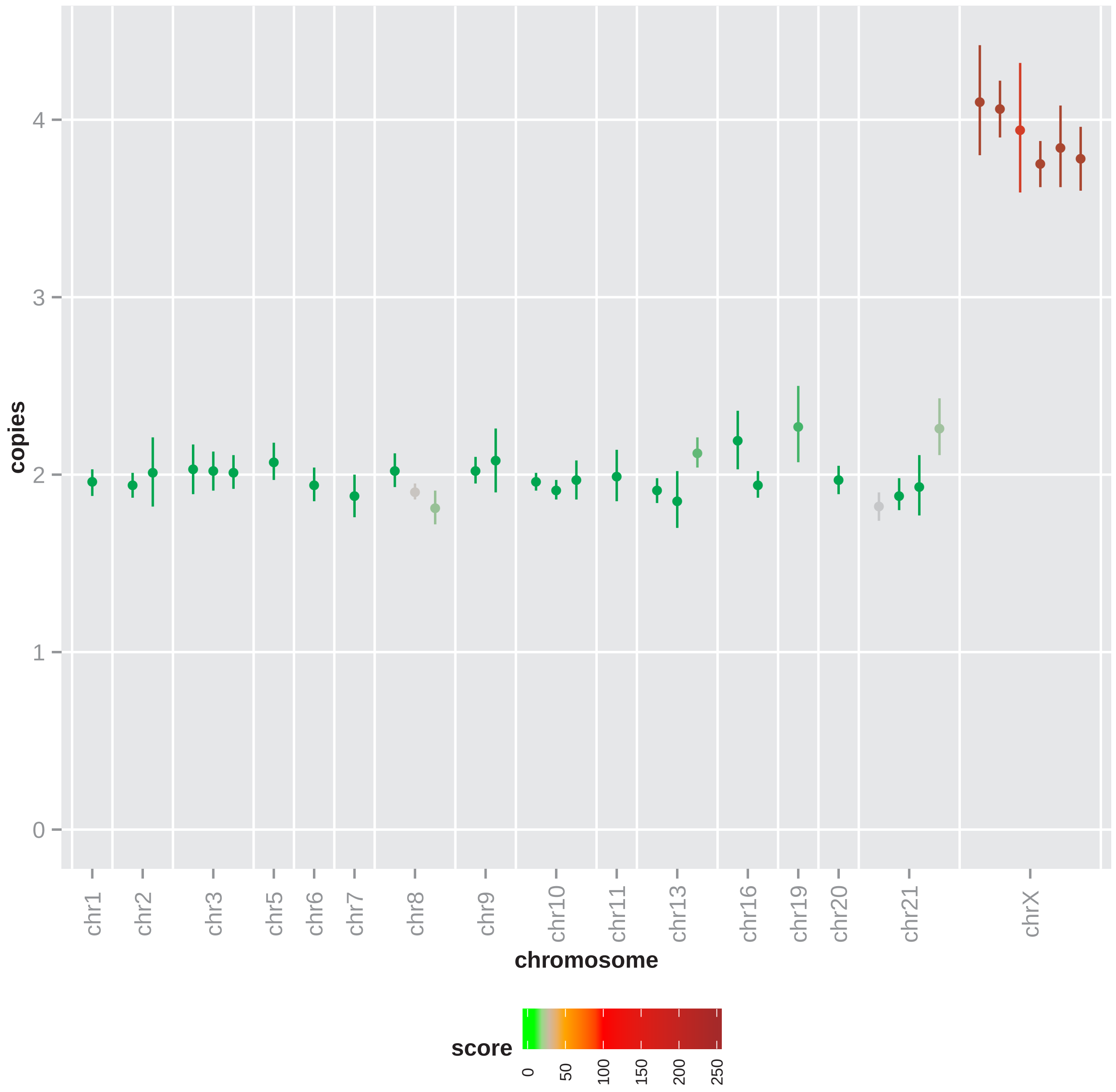

QIAGEN®

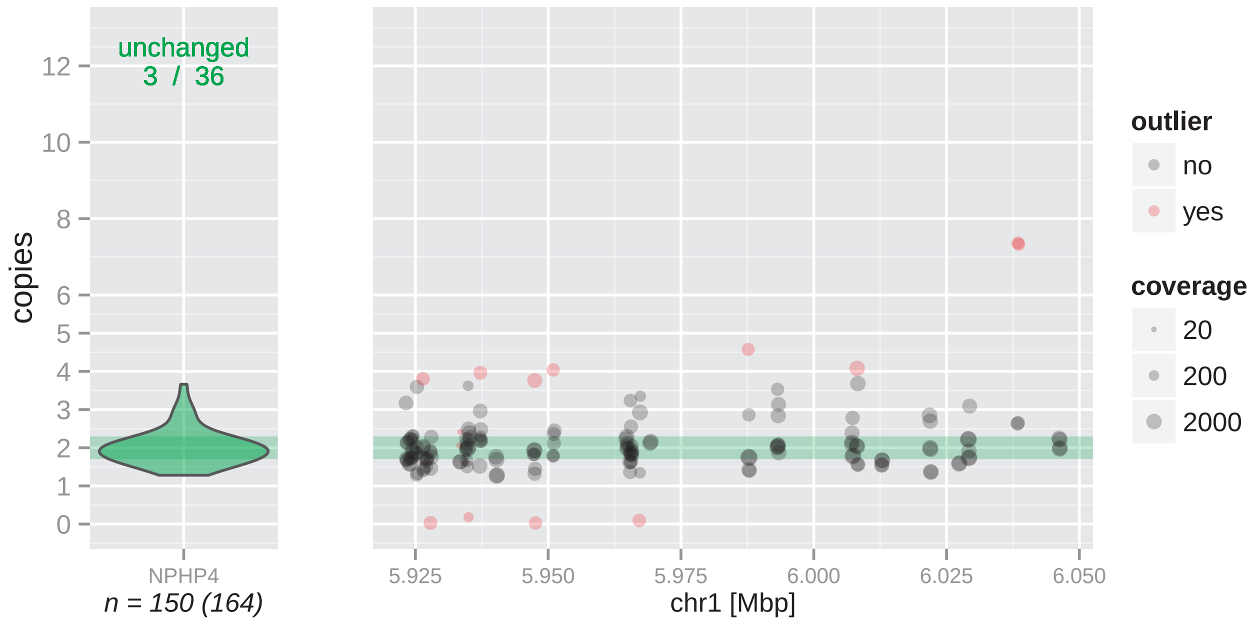
unchanged
3 / 36
copies
12
10
8
6
5
4
3
2
1
0
NPHP4
n = 150 (164)
5.925
5.950
5.975
6.000
6.025
6.050
chr1 [Mbp]
outlier
no
yes
coverage
20
200
2000

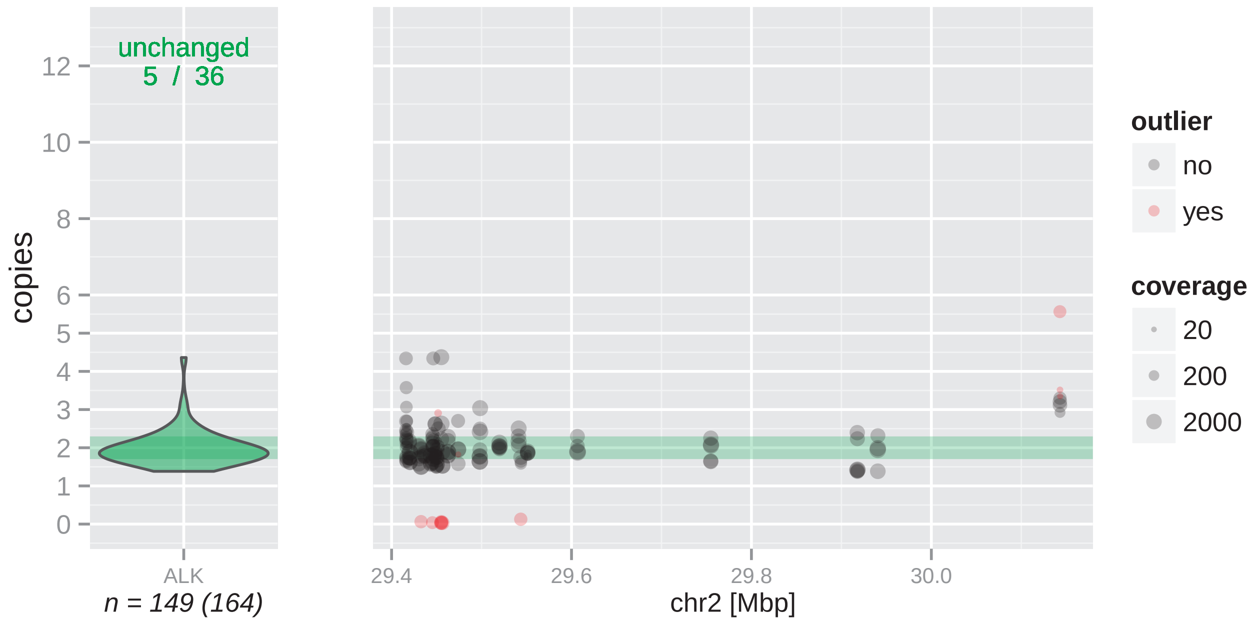
unchanged
5 / 36
copies
12
10
8
6
5
4
3
2
1
0
ALK
n = 149 (164)
29.4
29.6
29.8
30.0
chr2 [Mbp]
outlier
no
yes
coverage
20
200
2000

QIAGEN GeneRead Copy Number Analysis

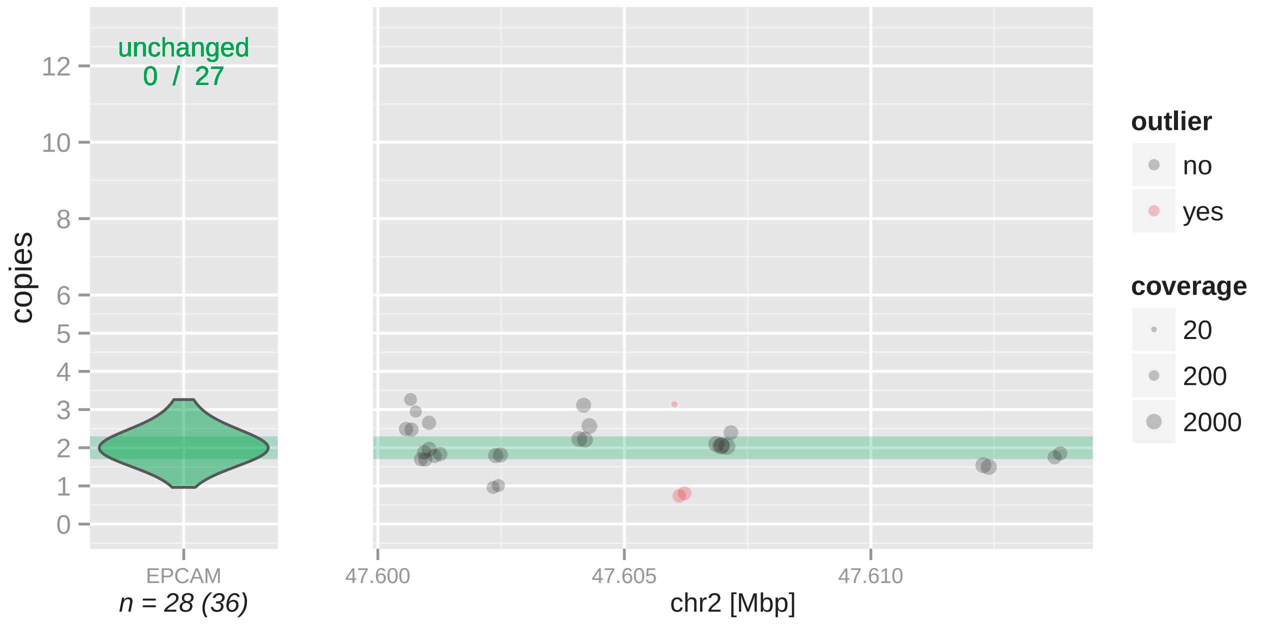

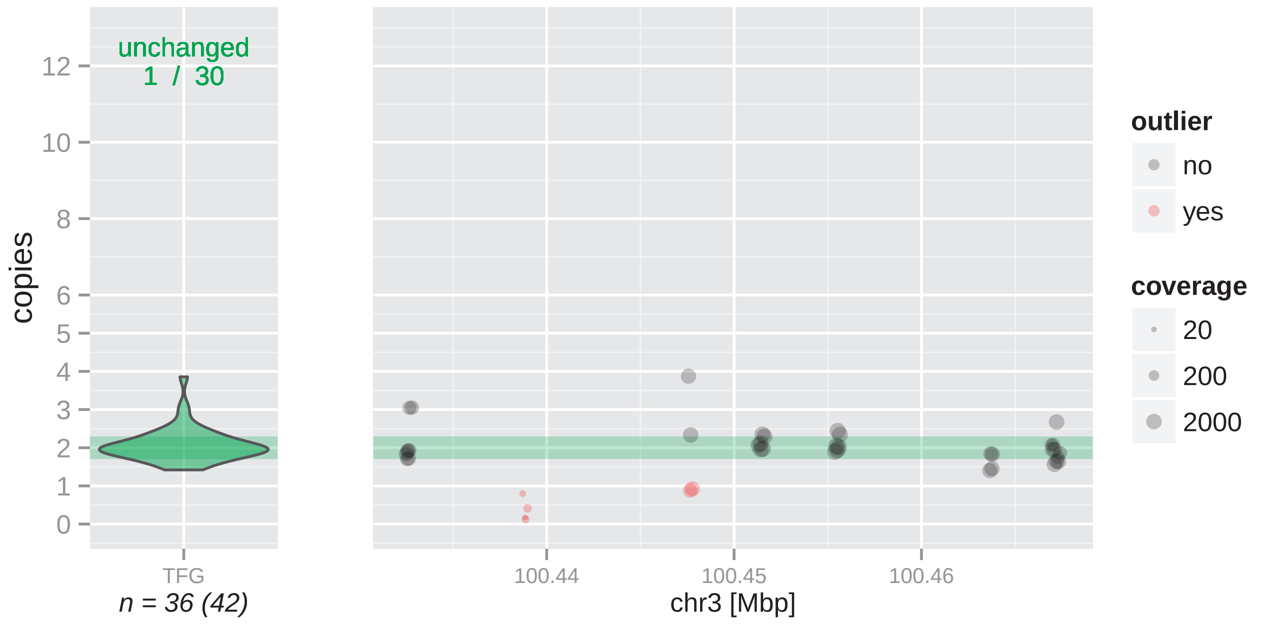

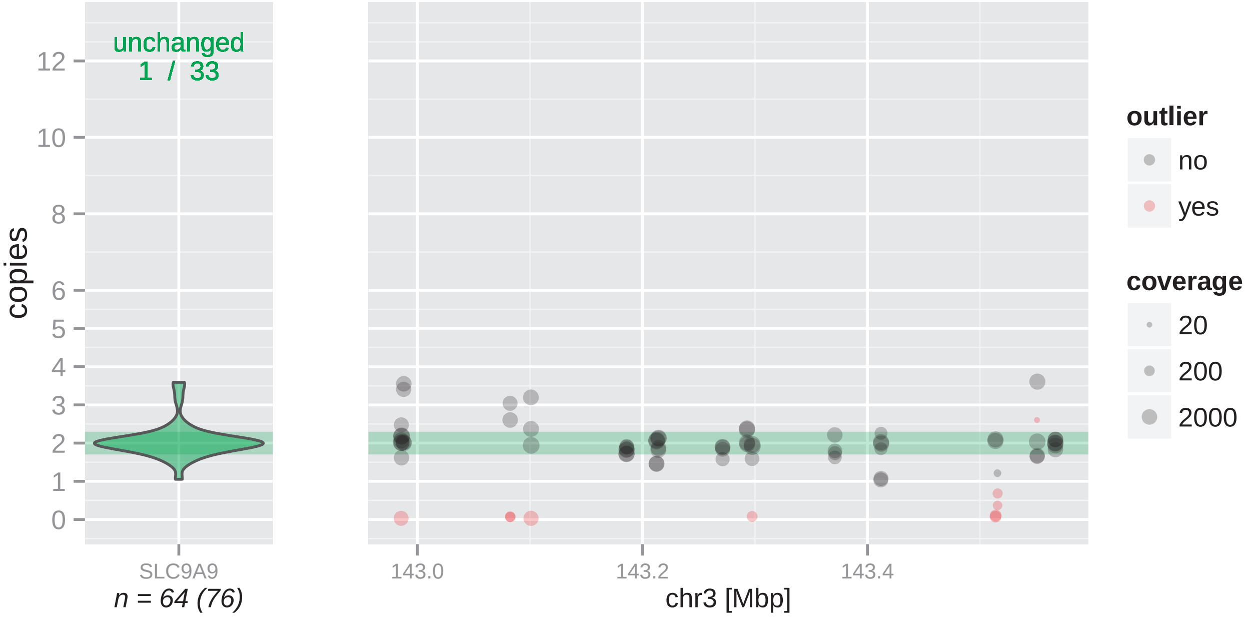
unchanged
1 / 33
copies
12
10
8
6
5
4
3
2
1
0
SLC9A9
n = 64 (76)
143.0
143.2
143.4
chr3 [Mbp]
outlier
no
yes
coverage
20
200
2000

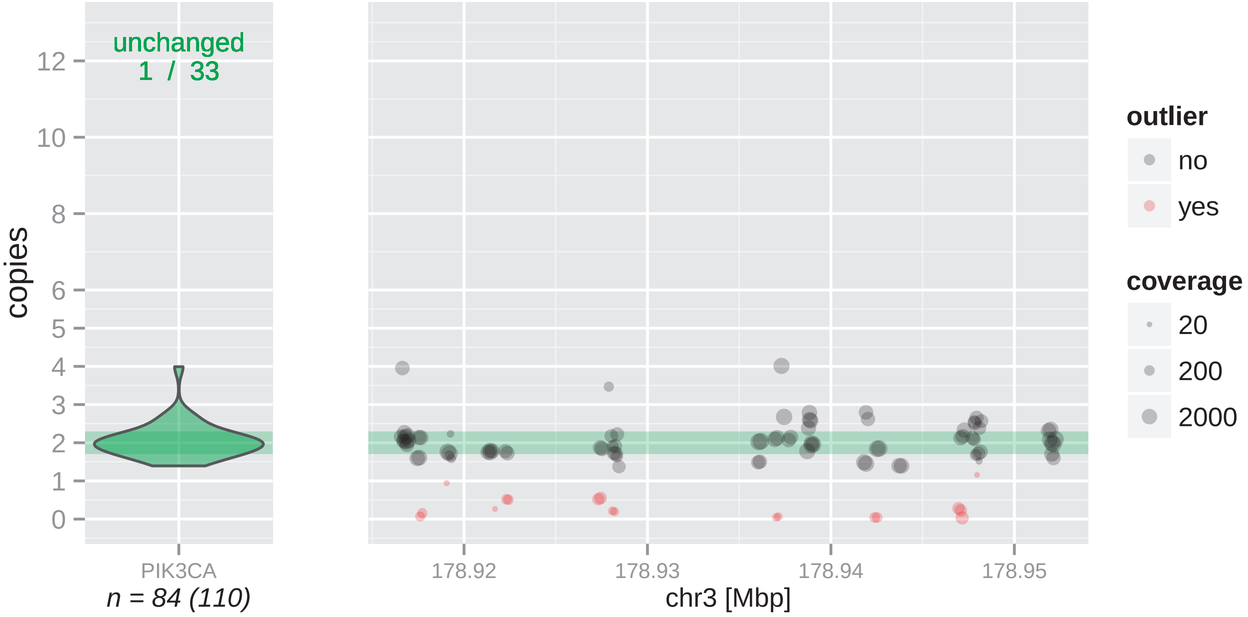
unchanged
1 / 33
copies
12
10
8
6
5
4
3
2
1
0
PIK3CA
n = 84 (110)
178.92
178.93
178.94
178.95
chr3 [Mbp]
outlier
no
yes
coverage
20
200
2000

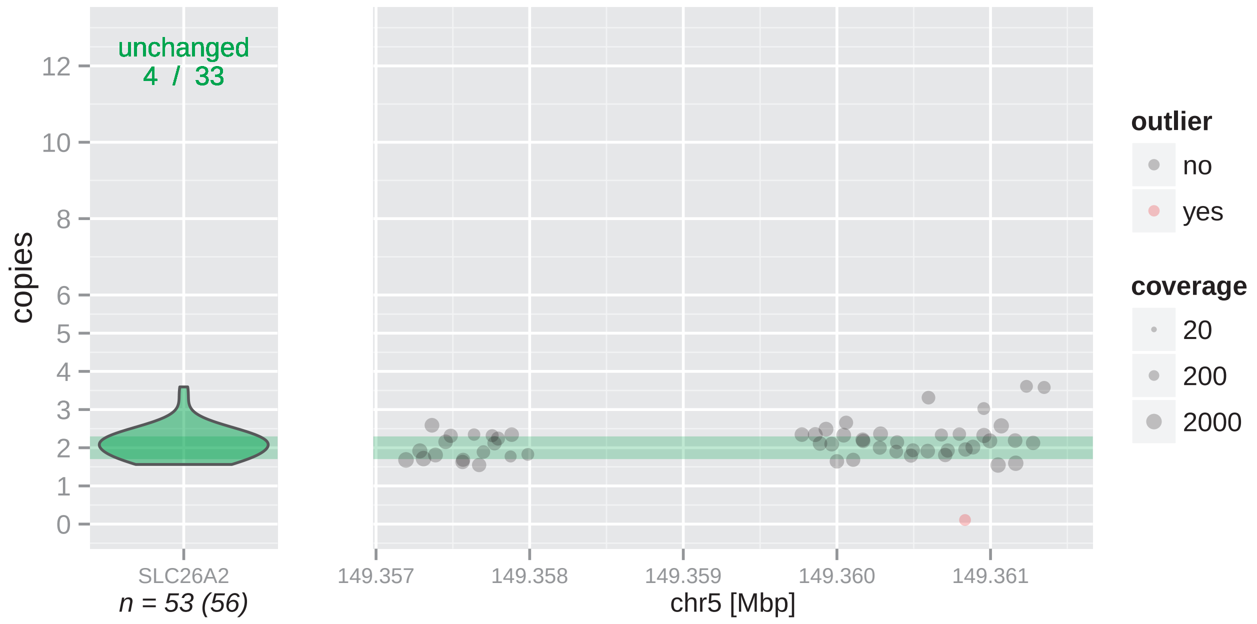
unchanged
4 / 33
copies
12
10
8
6
5
4
3
2
1
0
SLC26A2
n = 53 (56)
149.357
149.358
149.359
149.360
149.361
chr5 [Mbp]
outlier
no
yes
coverage
20
200
2000

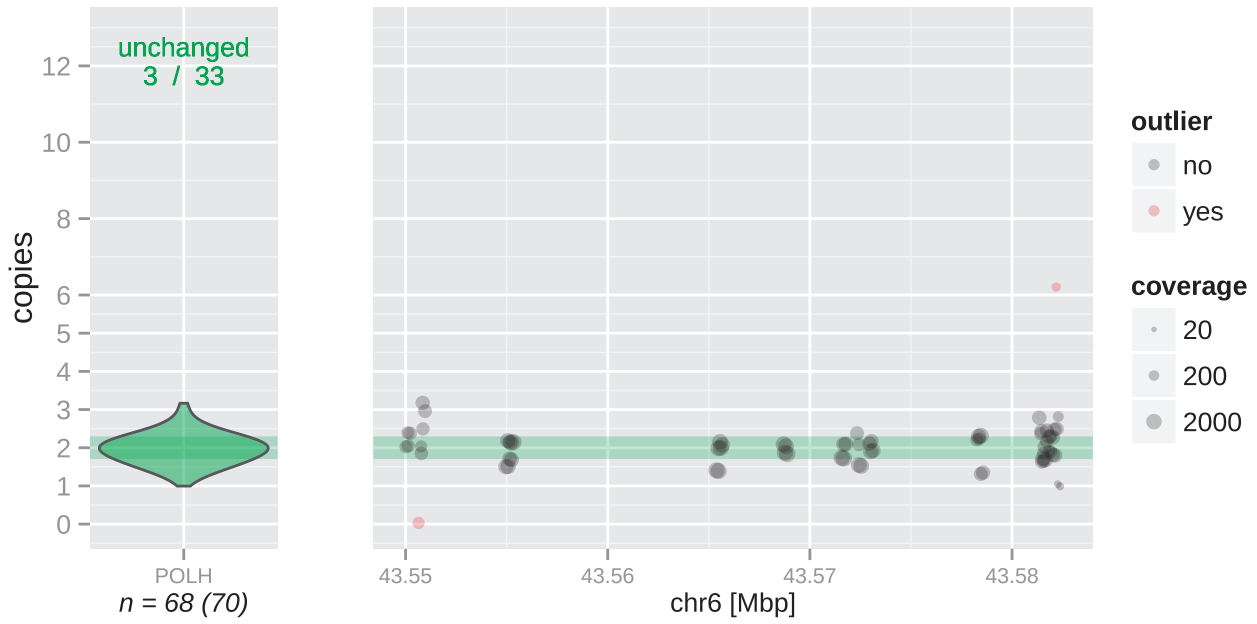
unchanged
3 / 33
copies
12
10
8
6
5
4
3
2
1
0
POLH
n = 68 (70)
43.55
43.56
43.57
43.58
chr6 [Mbp]
outlier
no
yes
coverage
20
200
2000

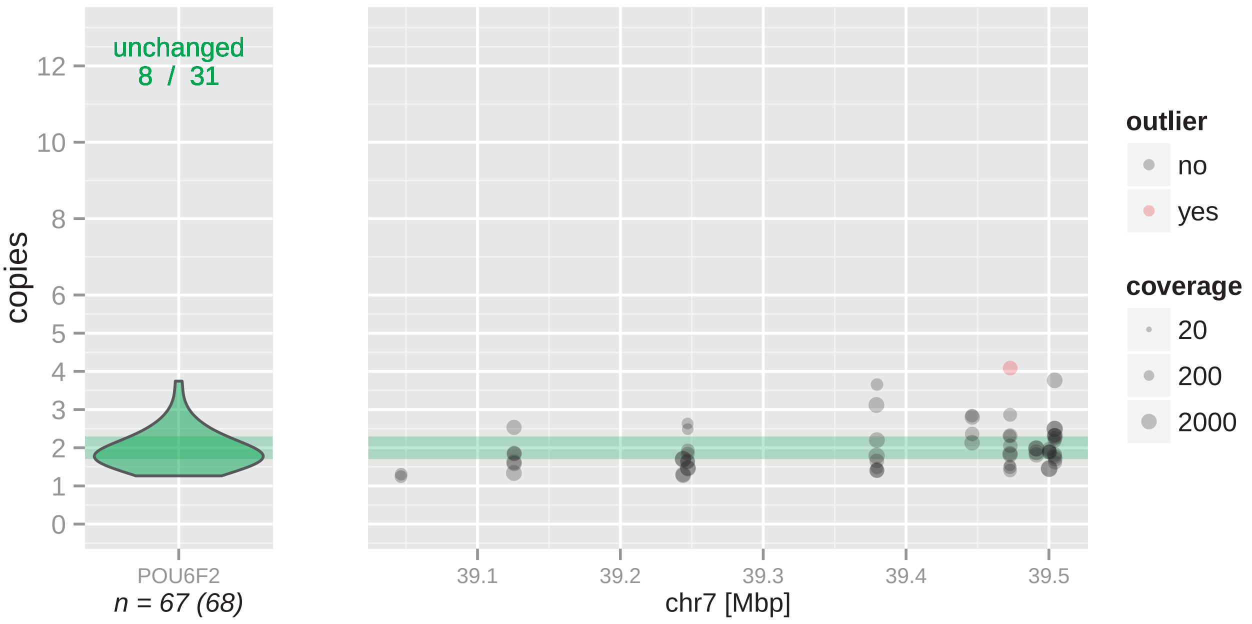
unchanged
8 / 31
copies
12
10
8
6
5
4
3
2
1
0
POU6F2
n = 67 (68)
39.1
39.2
39.3
39.4
39.5
chr7 [Mbp]
outlier
no
yes
coverage
20
200
2000

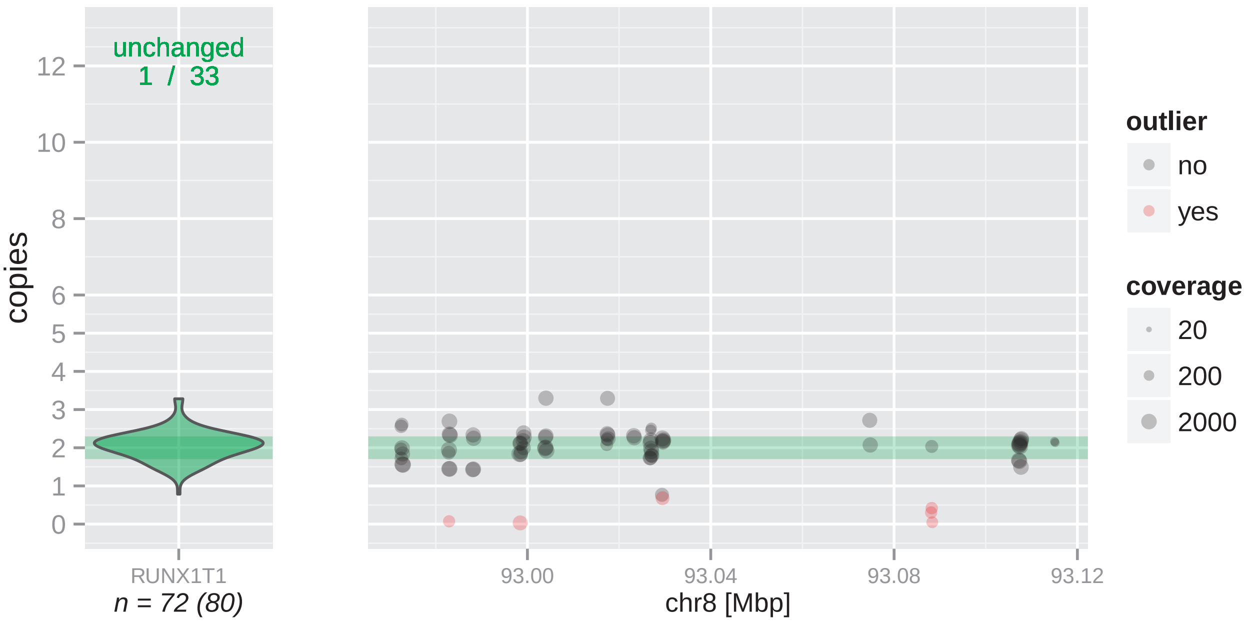
unchanged
1 / 33
copies
12
10
8
6
5
4
3
2
1
0
RUNX1T1
n = 72 (80)
93.00
93.04
93.08
93.12
chr8 [Mbp]
outlier
no
yes
coverage
20
200
2000

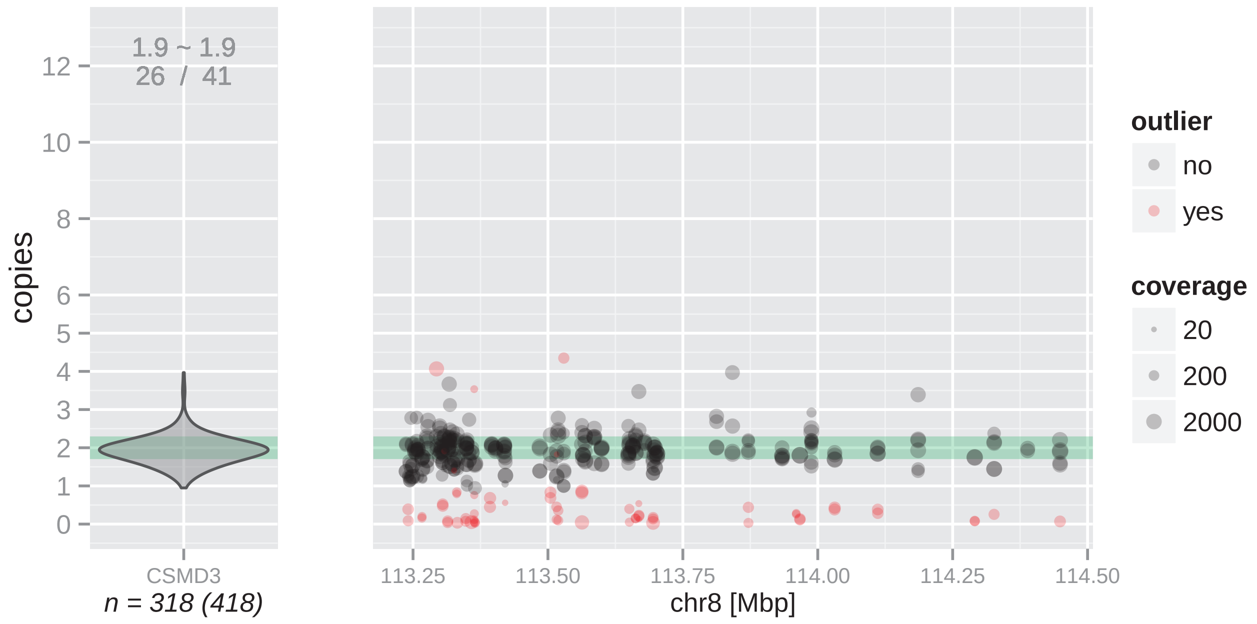

1.9 ~ 1.9
26 / 41
copies
12
10
8
6
5
4
3
2
1
0
CSMD3
n = 318 (418)
113.25
113.50
113.75
114.00
114.25
114.50
chr8 [Mbp]
outlier
no
yes
coverage
20
200
2000

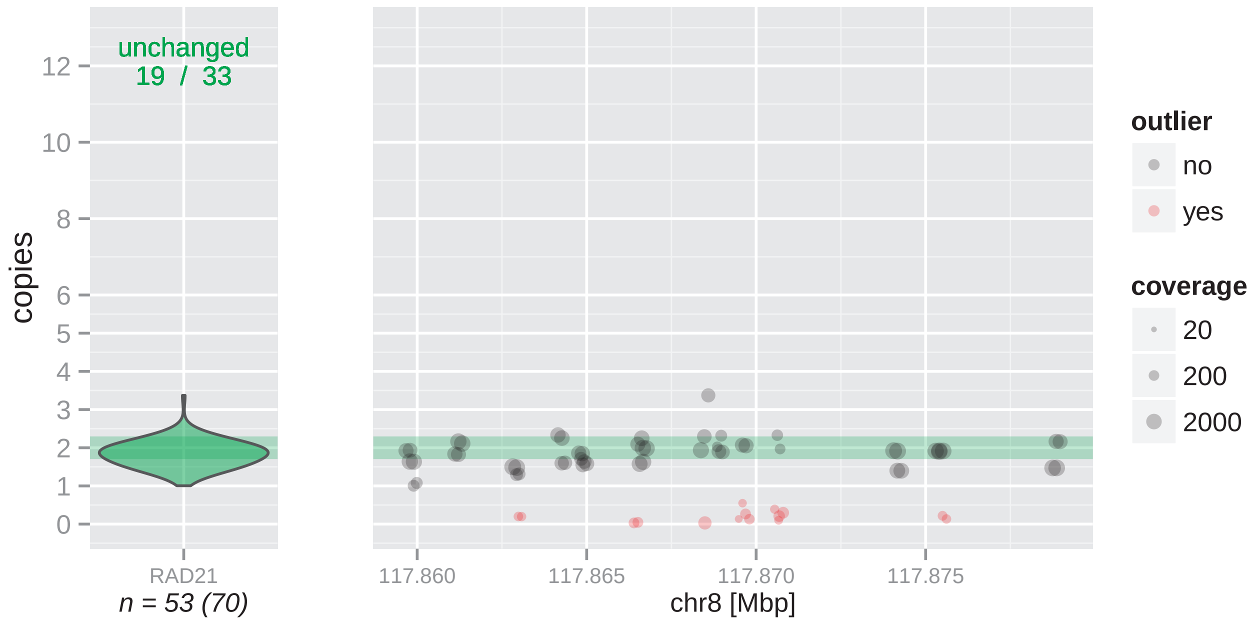

unchanged
19 / 33
copies
12
10
8
6
5
4
3
2
1
0
RAD21
n = 53 (70)
117.860
117.865
117.870
117.875
chr8 [Mbp]
outlier
no
yes
coverage
20
200
2000

QIAGEN GeneRead Copy Number Analysis

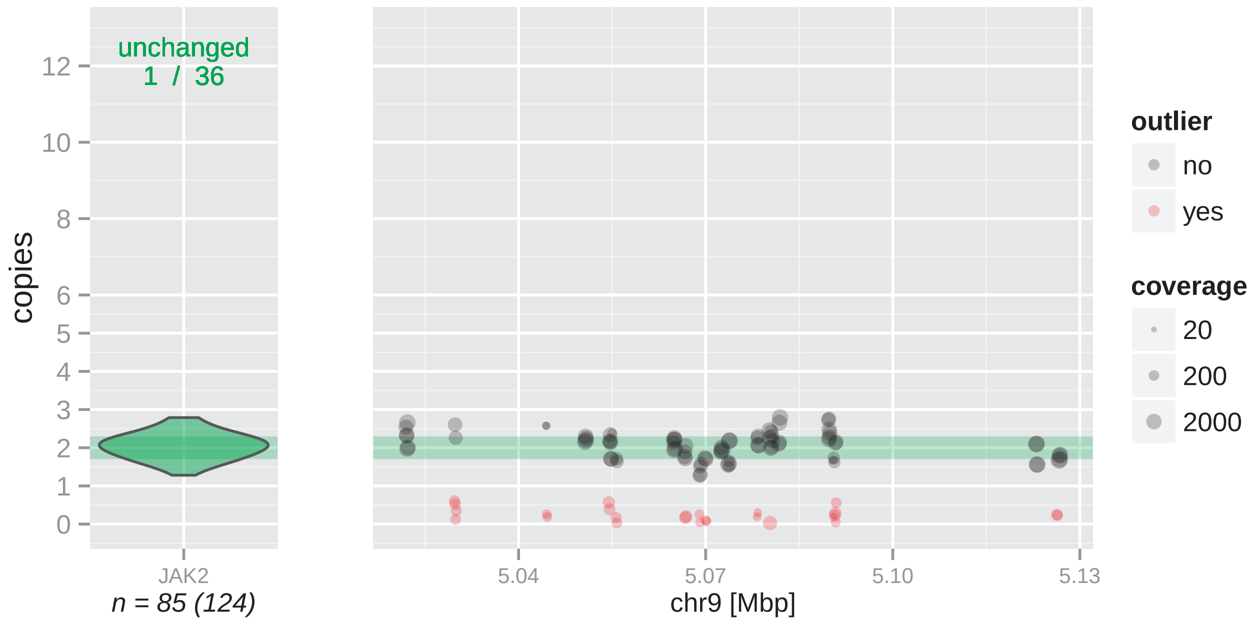

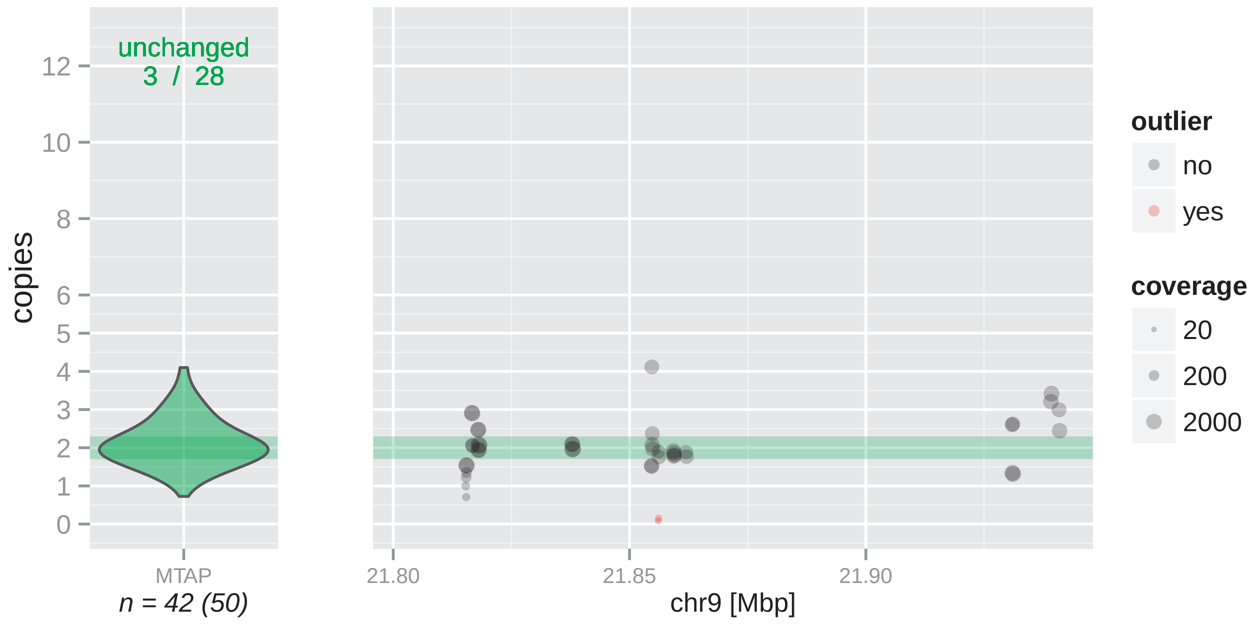

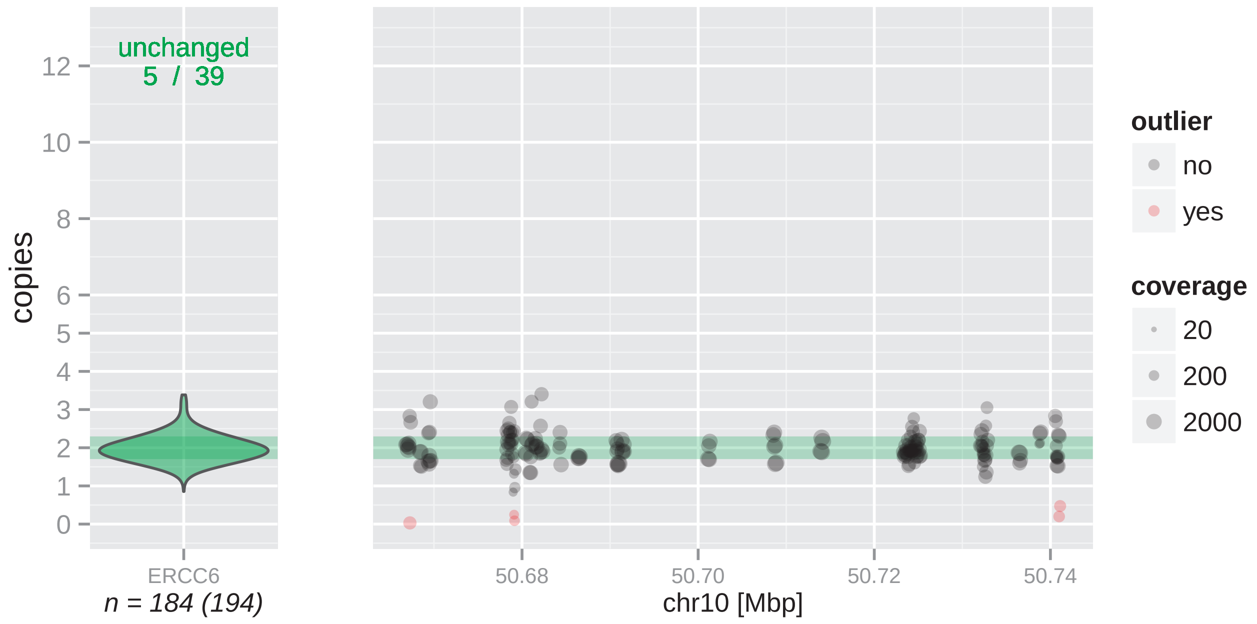
unchanged
5 / 39
copies
12
10
8
6
5
4
3
2
1
0
ERCC6
n = 184 (194)
50.68
50.70
50.72
50.74
chr10 [Mbp]
outlier
no
yes
coverage
20
200
2000

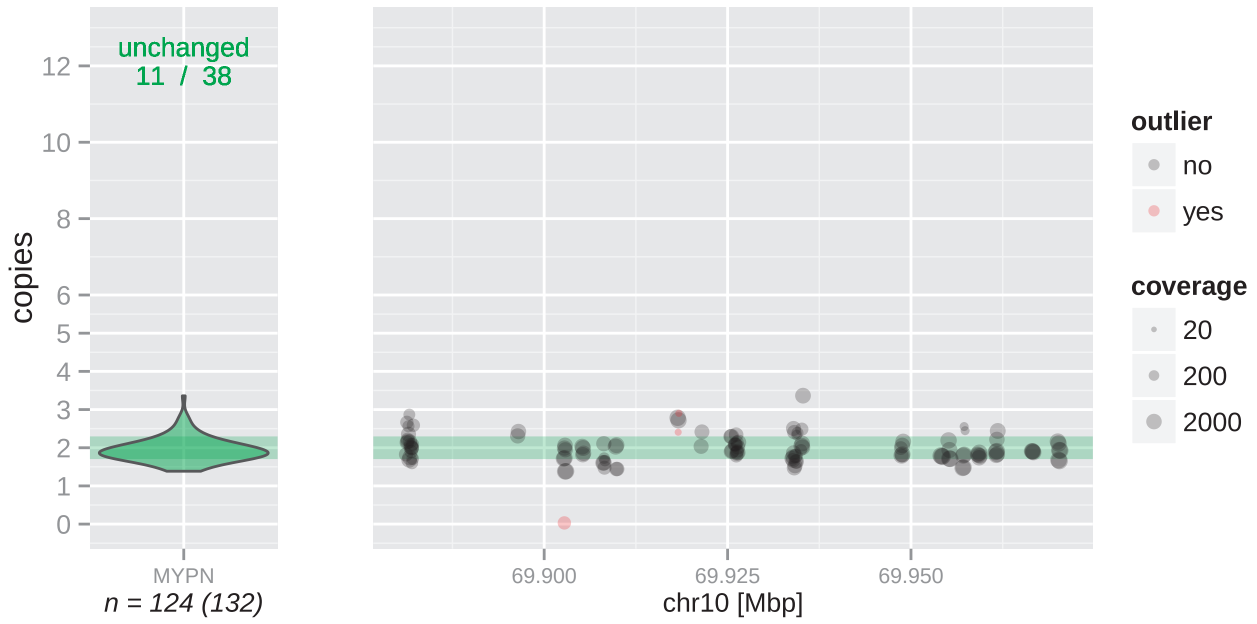
unchanged
11 / 38
copies
12
10
8
6
5
4
3
2
1
0
MYPN
n = 124 (132)
69.900
69.925
69.950
chr10 [Mbp]
outlier
no
yes
coverage
20
200
2000

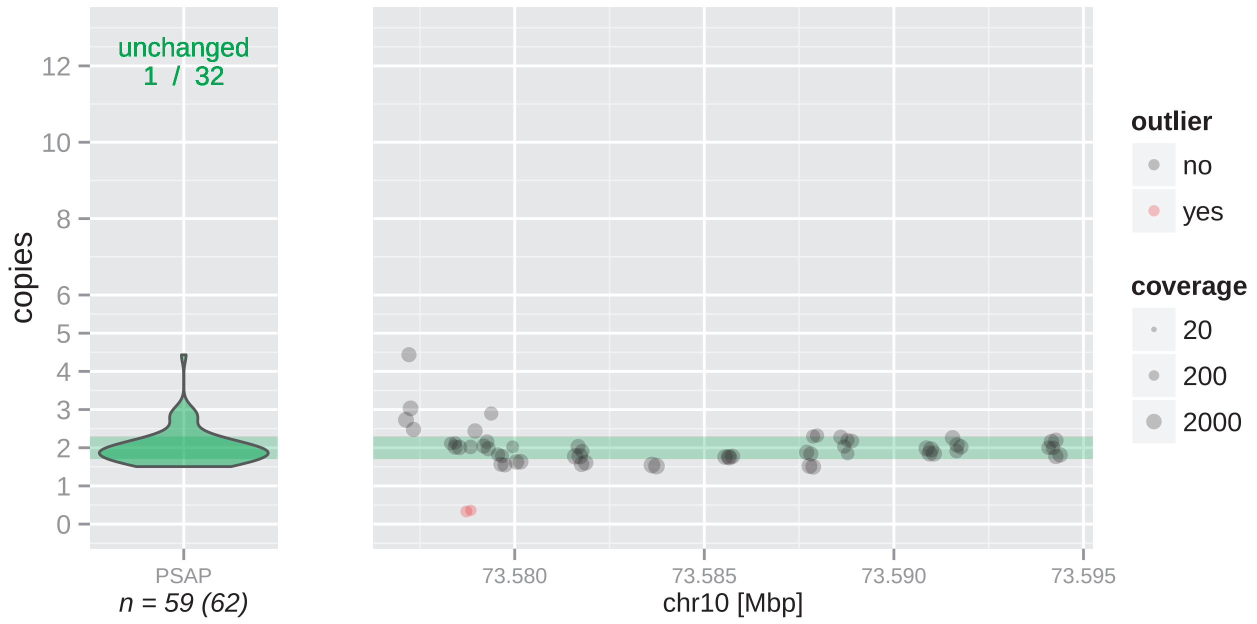
unchanged
1 / 32
copies
12
10
8
6
5
4
3
2
1
0
PSAP
n = 59 (62)
73.580
73.585
73.590
73.595
chr10 [Mbp]
outlier
no
yes
coverage
20
200
2000

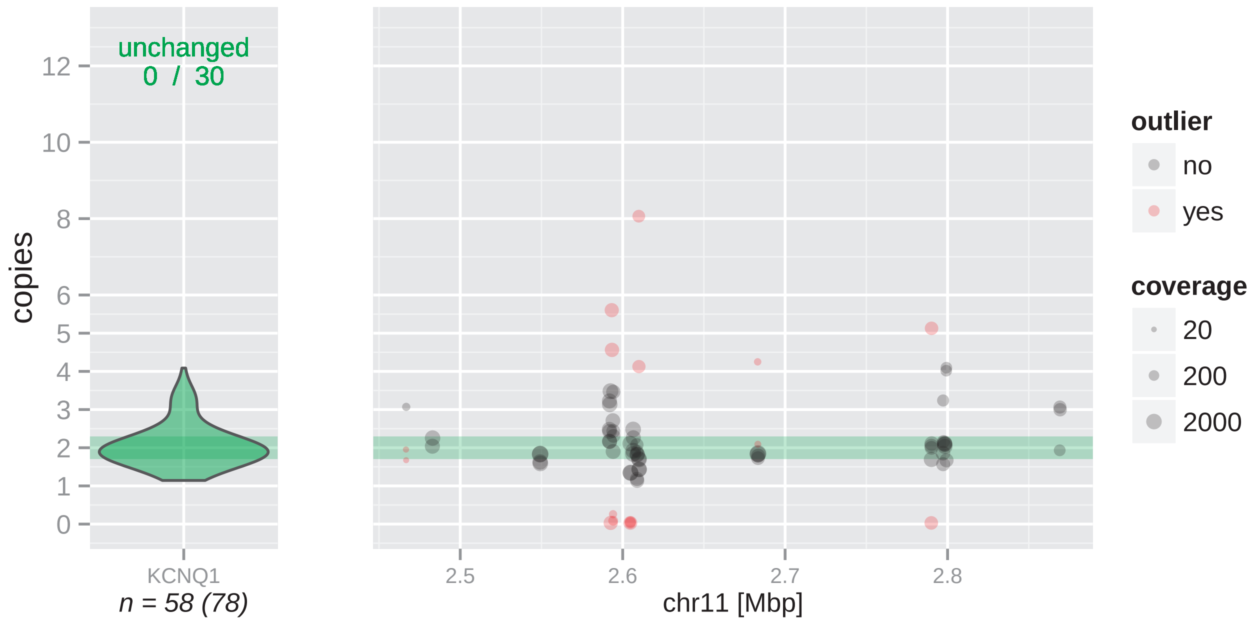
unchanged
0 / 30
copies
12
10
8
6
5
4
3
2
1
0
KCNQ1
n = 58 (78)
2.5
2.6
2.7
2.8
chr11 [Mbp]
outlier
no
yes
coverage
20
200
2000

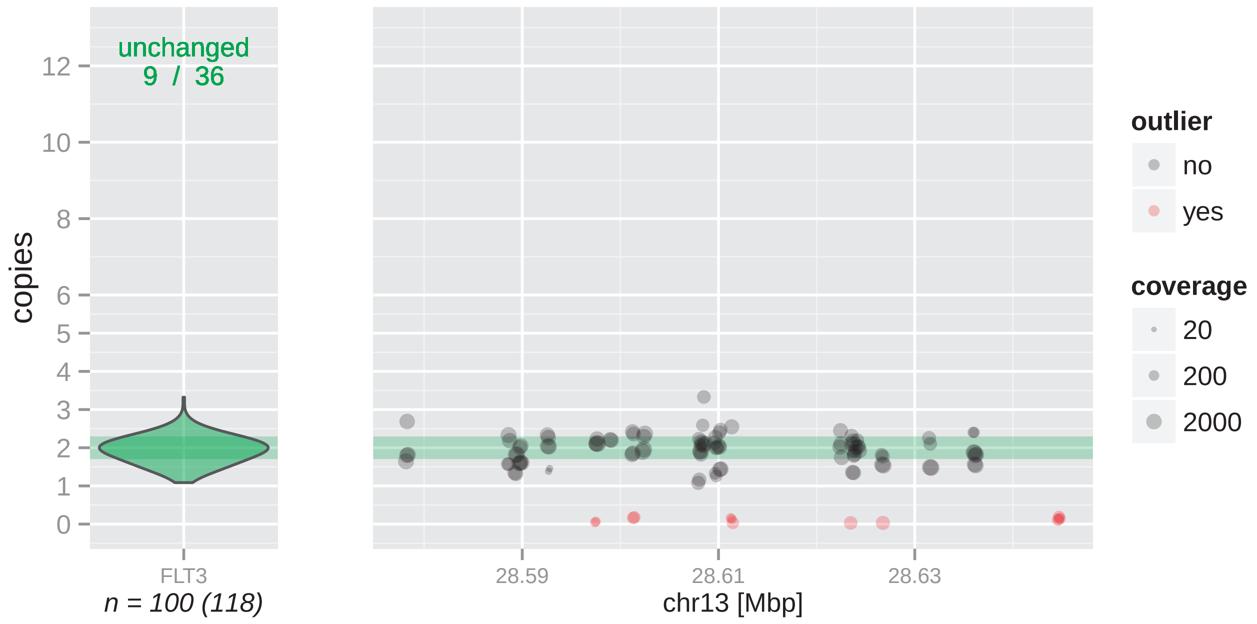
unchanged
9 / 36
copies
12
10
8
6
5
4
3
2
1
0
FLT3
n = 100 (118)
28.59
28.61
28.63
chr13 [Mbp]
outlier
no
yes
coverage
20
200
2000

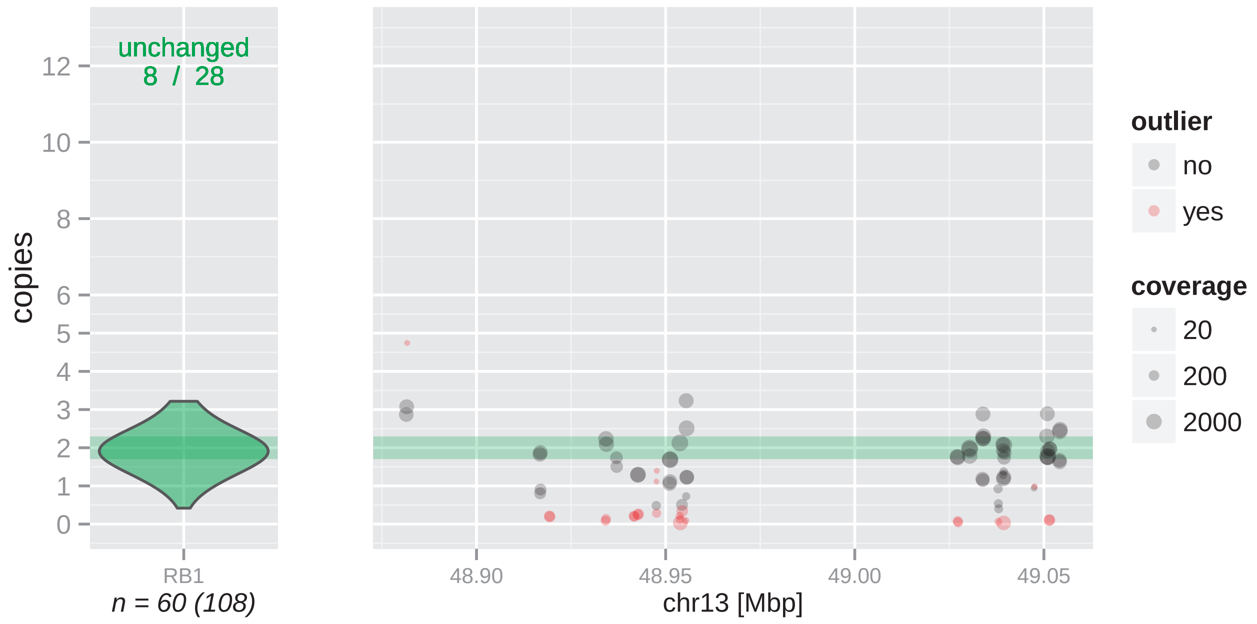
unchanged
8 / 28
copies
12
10
8
6
5
4
3
2
1
0
RB1
n = 60 (108)
48.90
48.95
49.00
49.05
chr13 [Mbp]
outlier
no
yes
coverage
20
200
2000

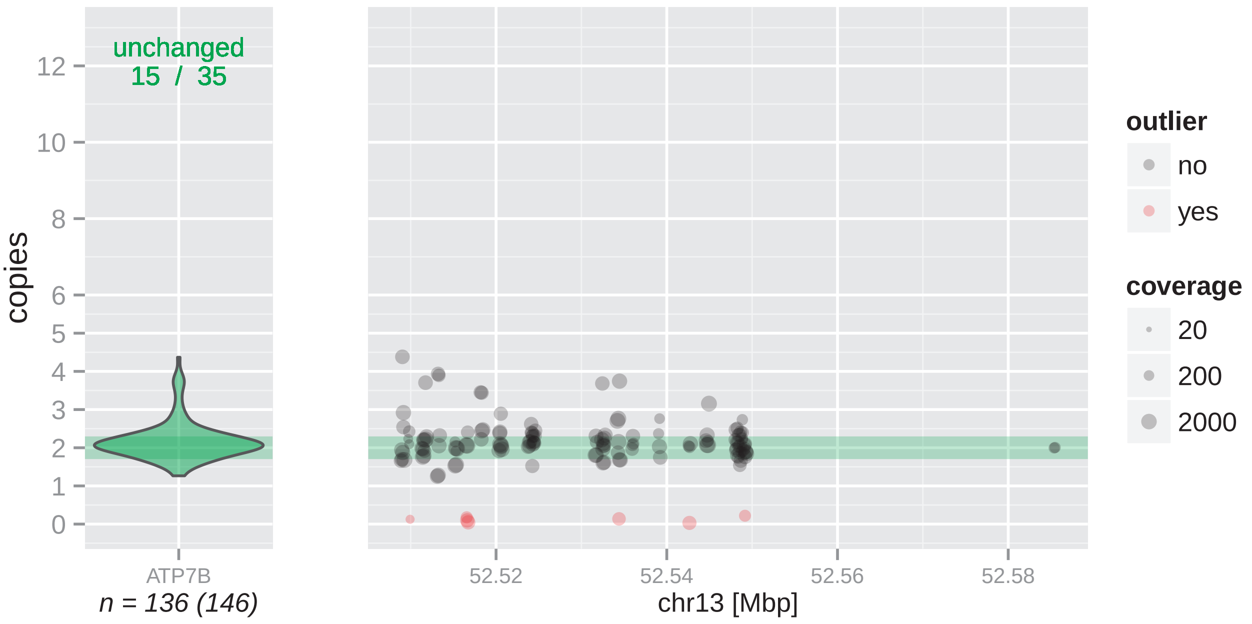
unchanged
15 / 35
copies
12
10
8
6
5
4
3
2
1
0
ATP7B
n = 136 (146)
52.52
52.54
52.56
52.58
chr13 [Mbp]
outlier
no
yes
coverage
20
200
2000

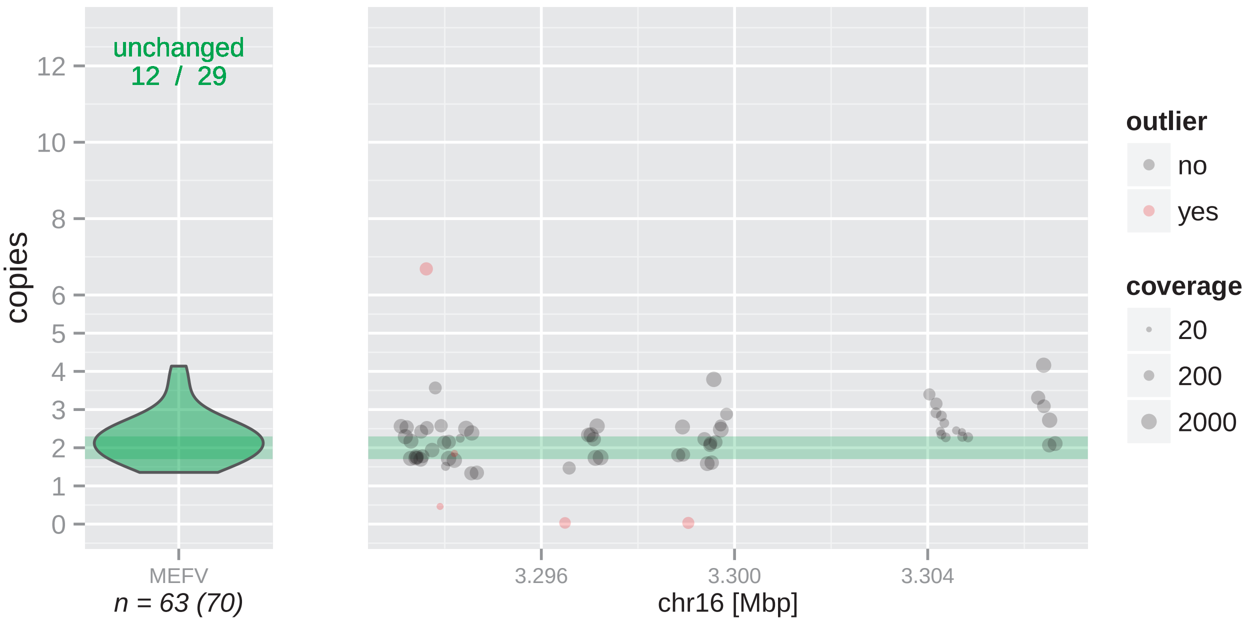
unchanged
12 / 29
copies
12
10
8
6
5
4
3
2
1
0
MEFV
n = 63 (70)
3.296
3.300
3.304
chr16 [Mbp]
outlier
no
yes
coverage
20
200
2000

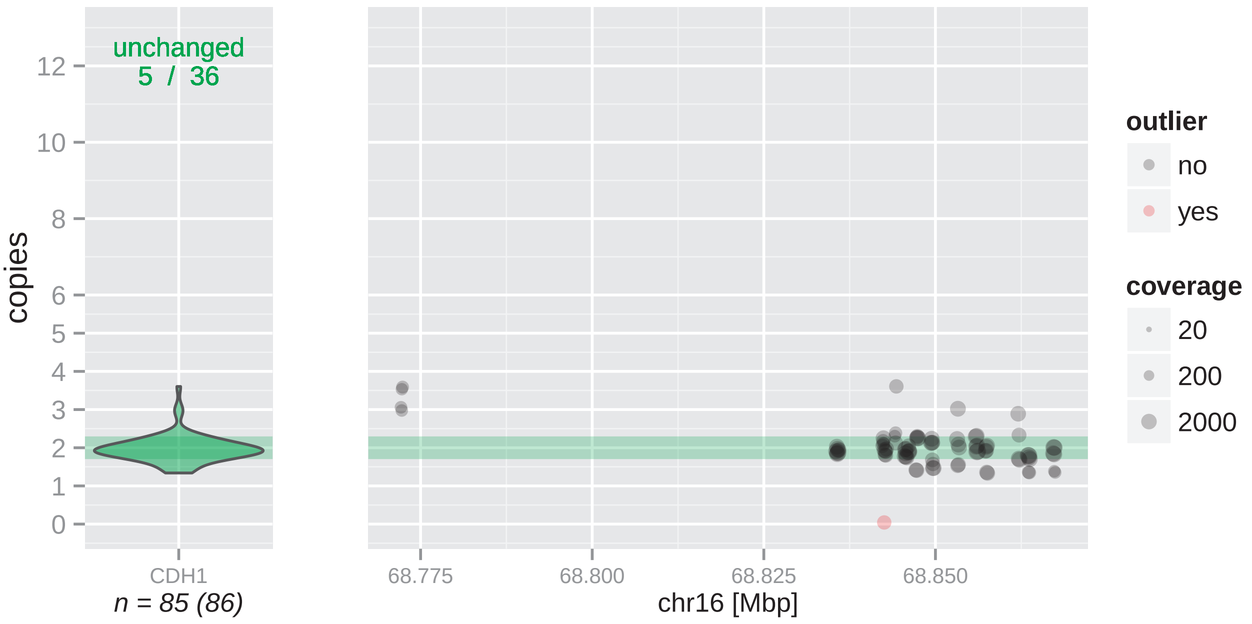
unchanged
5 / 36
copies
CDH1
n = 85 (86)
68.775
68.800
68.825
68.850
chr16 [Mbp]
outlier
no
yes
coverage
20
200
2000

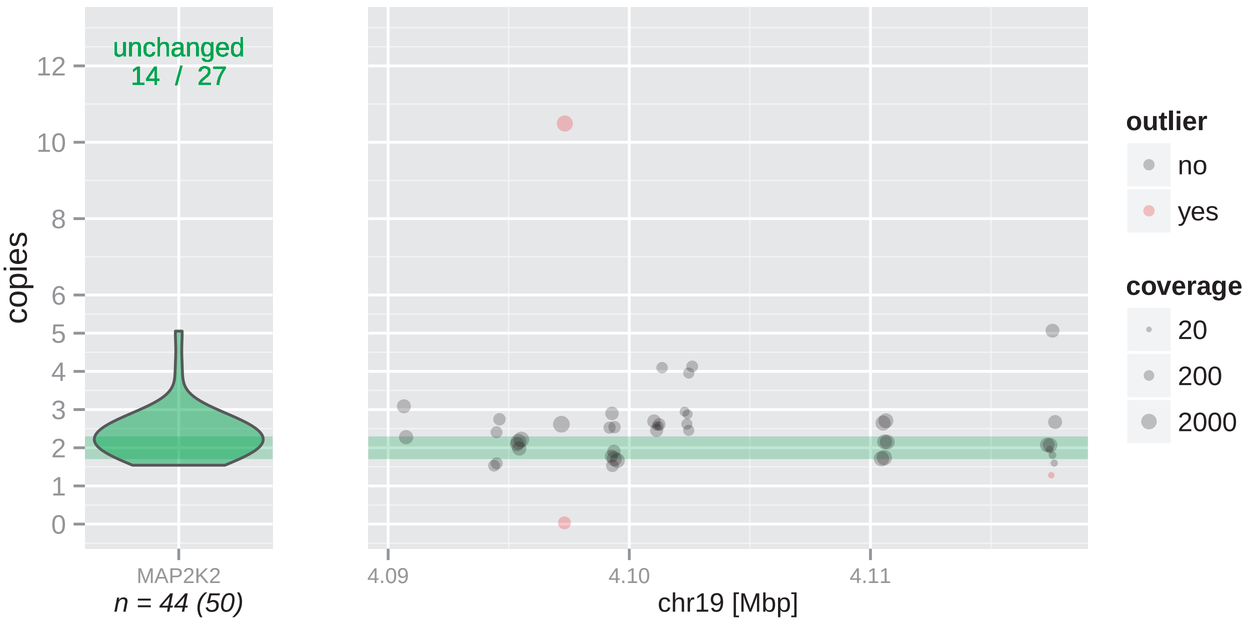
unchanged
14 / 27
copies
MAP2K2
n = 44 (50)
4.09
4.10
4.11
chr19 [Mbp]
outlier
no
yes
coverage
20
200
2000

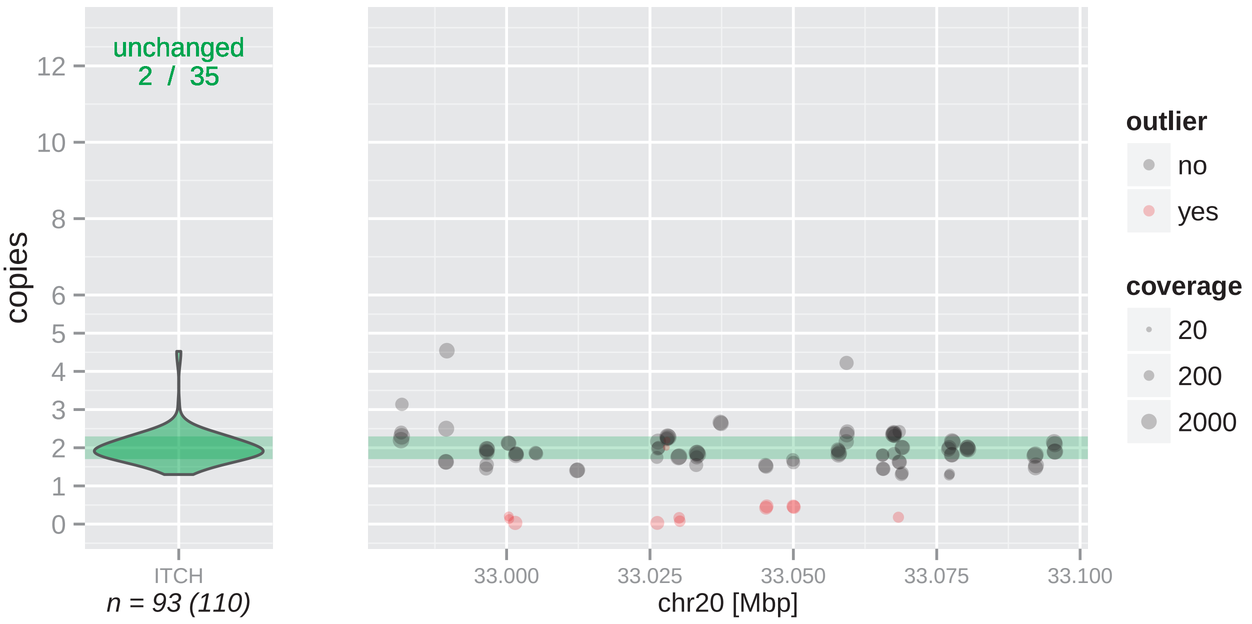
unchanged
2 / 35
copies
12
10
8
6
5
4
3
2
1
0
ITCH
n = 93 (110)
33.000
33.025
33.050
33.075
33.100
chr20 [Mbp]
outlier
no
yes
coverage
20
200
2000

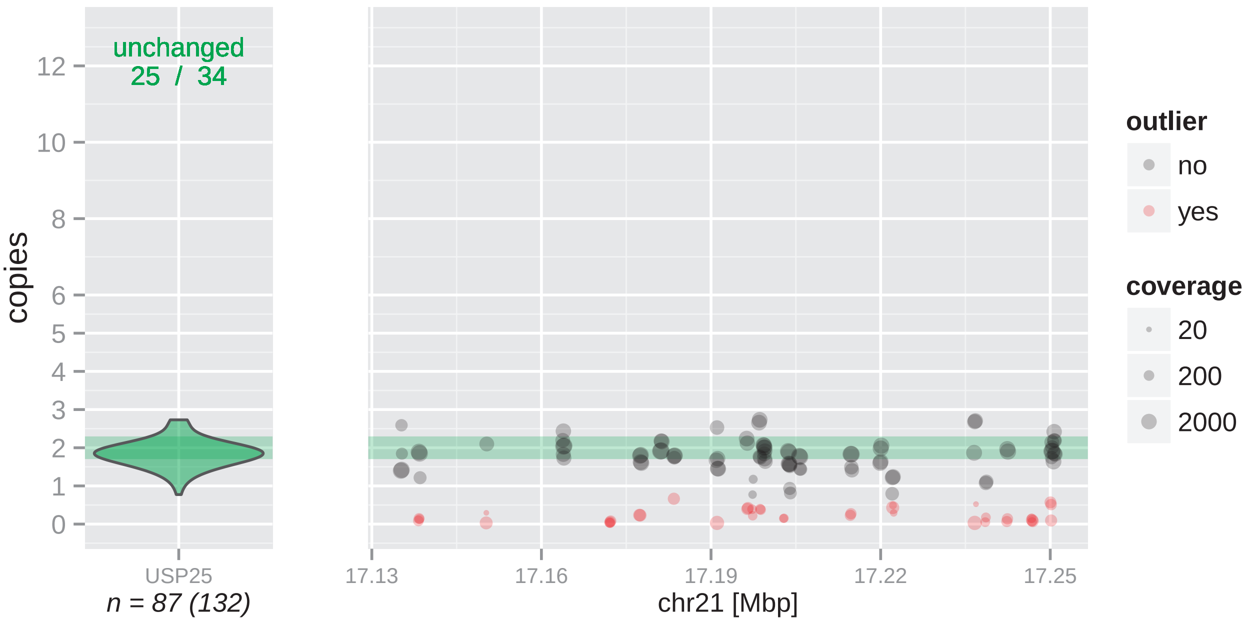
unchanged
25 / 34
copies
12
10
8
6
5
4
3
2
1
0
USP25
n = 87 (132)
17.13
17.16
17.19
17.22
17.25
chr21 [Mbp]
outlier
no
yes
coverage
20
200
2000

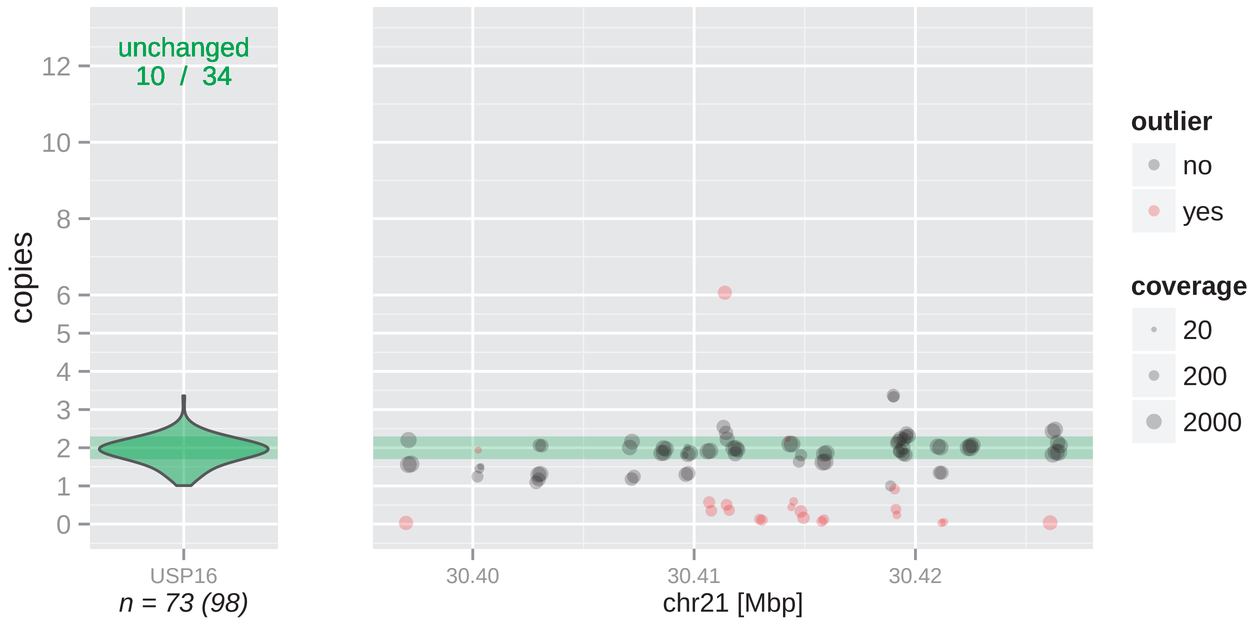
unchanged
10 / 34
copies
12
10
8
6
5
4
3
2
1
0
USP16
n = 73 (98)
30.40
30.41
30.42
chr21 [Mbp]
outlier
no
yes
coverage
20
200
2000

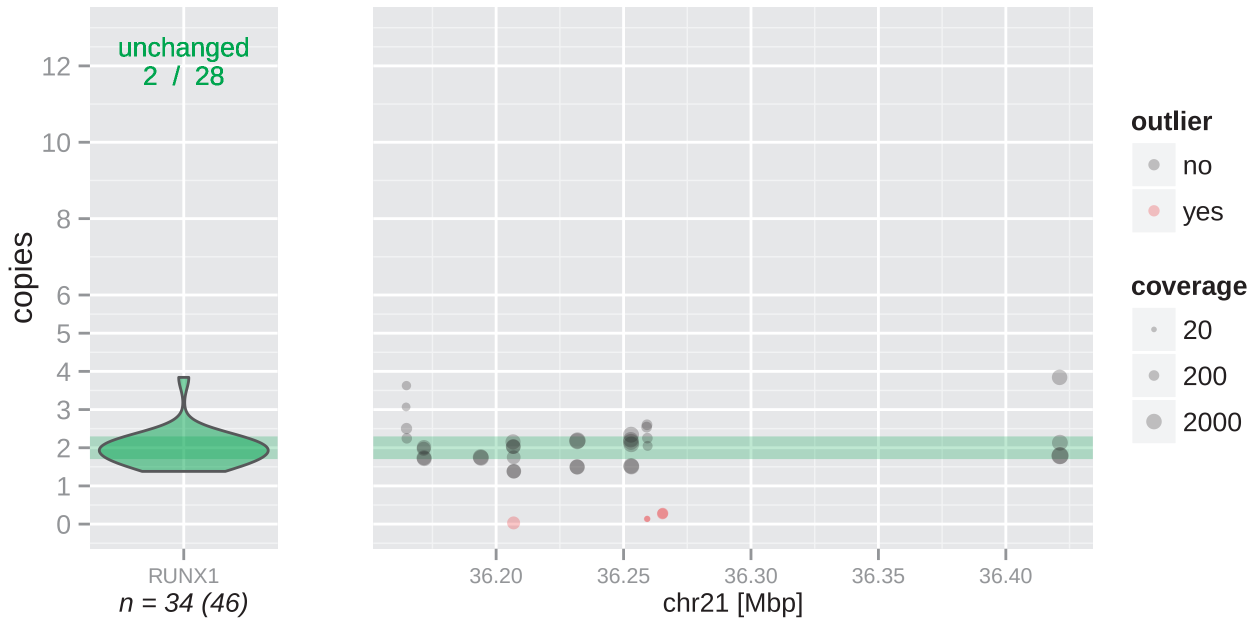
unchanged
2 / 28
copies
12
10
8
6
5
4
3
2
1
0
RUNX1
n = 34 (46)
36.20
36.25
36.30
36.35
36.40
chr21 [Mbp]
outlier
no
yes
coverage
20
200
2000

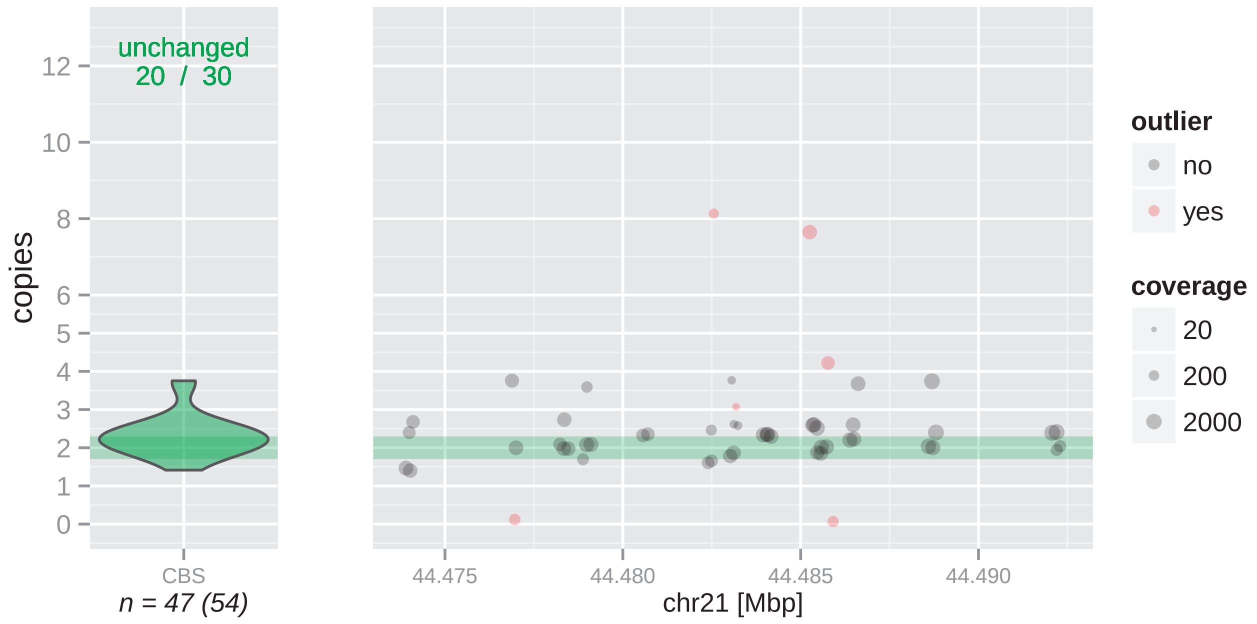

unchanged
20 / 30
copies
CBS
n = 47 (54)
44.475
44.480
44.485
44.490
chr21 [Mbp]
outlier
no
yes
coverage
20
200
2000

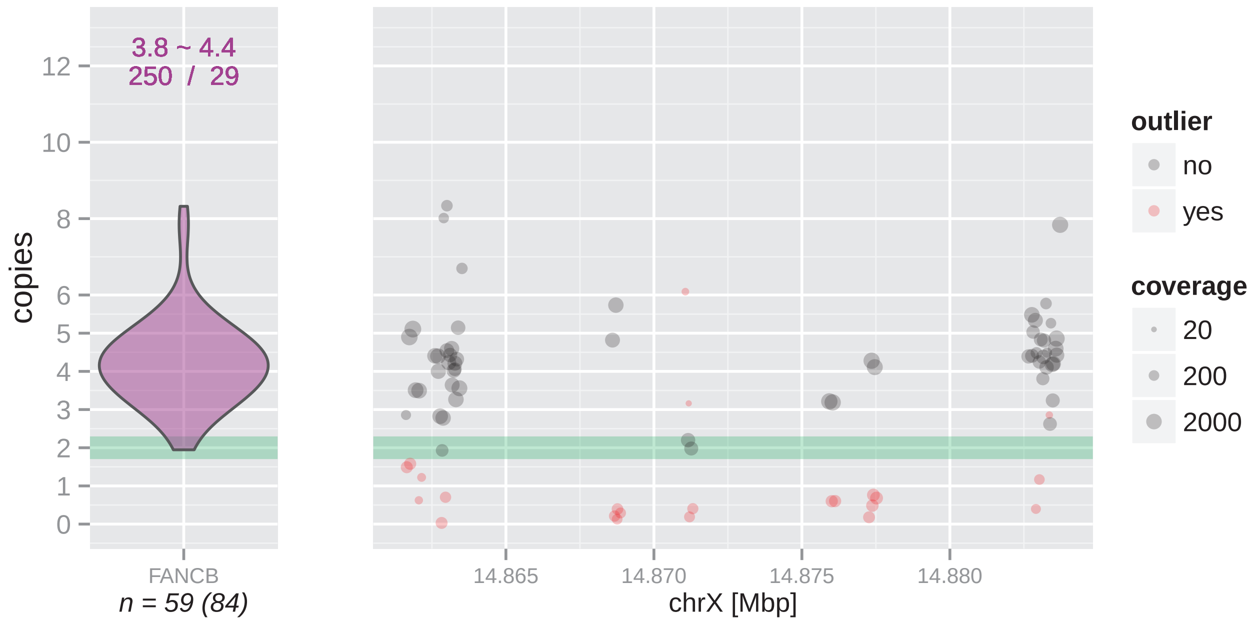

3.8 ~ 4.4
250 / 29
copies
FANCB
n = 59 (84)
14.865
14.870
14.875
14.880
chrX [Mbp]
outlier
no
yes
coverage
20
200
2000

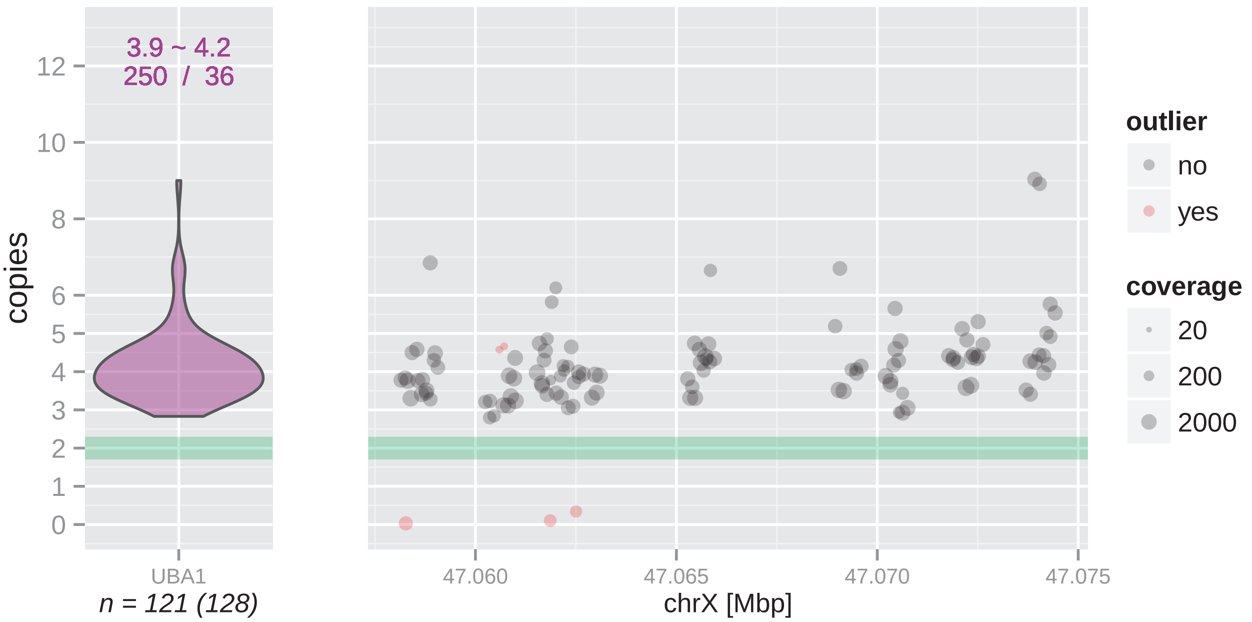
3.9 ~ 4.2
250 / 36
copies
12
10
8
6
5
4
3
2
1
0
UBA1
n = 121 (128)
47.060
47.065
47.070
47.075
chrX [Mbp]
outlier
no
yes
coverage
20
200
2000

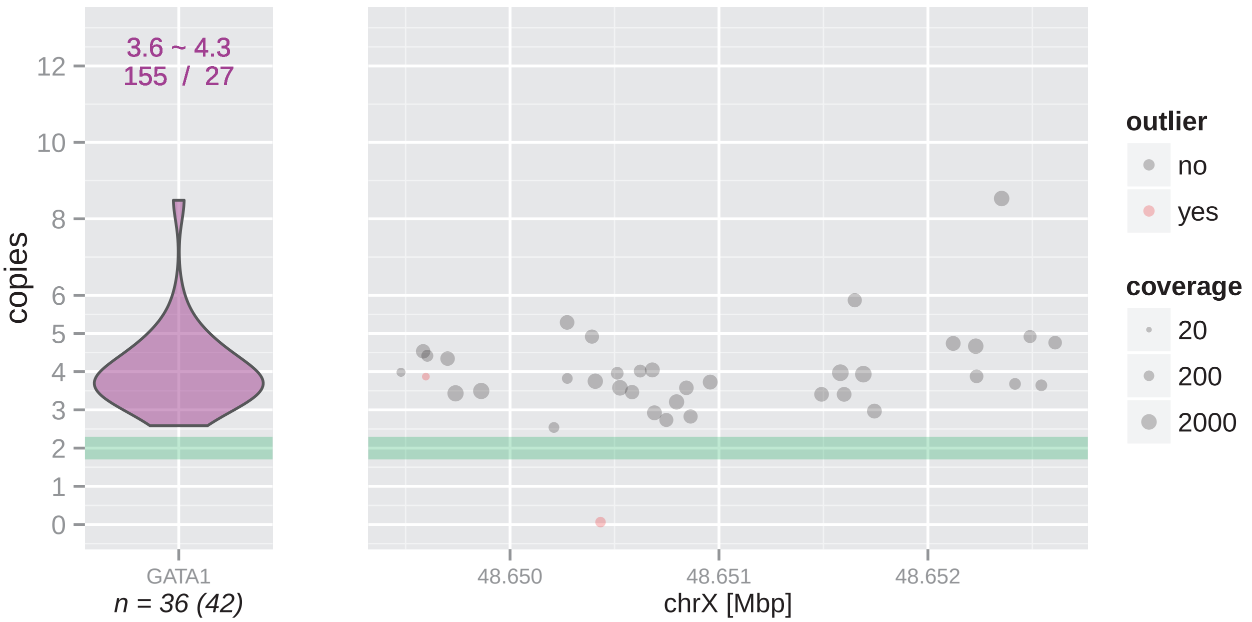
3.6 ~ 4.3
155 / 27
copies
12
10
8
6
5
4
3
2
1
0
GATA1
n = 36 (42)
48.650
48.651
48.652
chrX [Mbp]
outlier
no
yes
coverage
20
200
2000

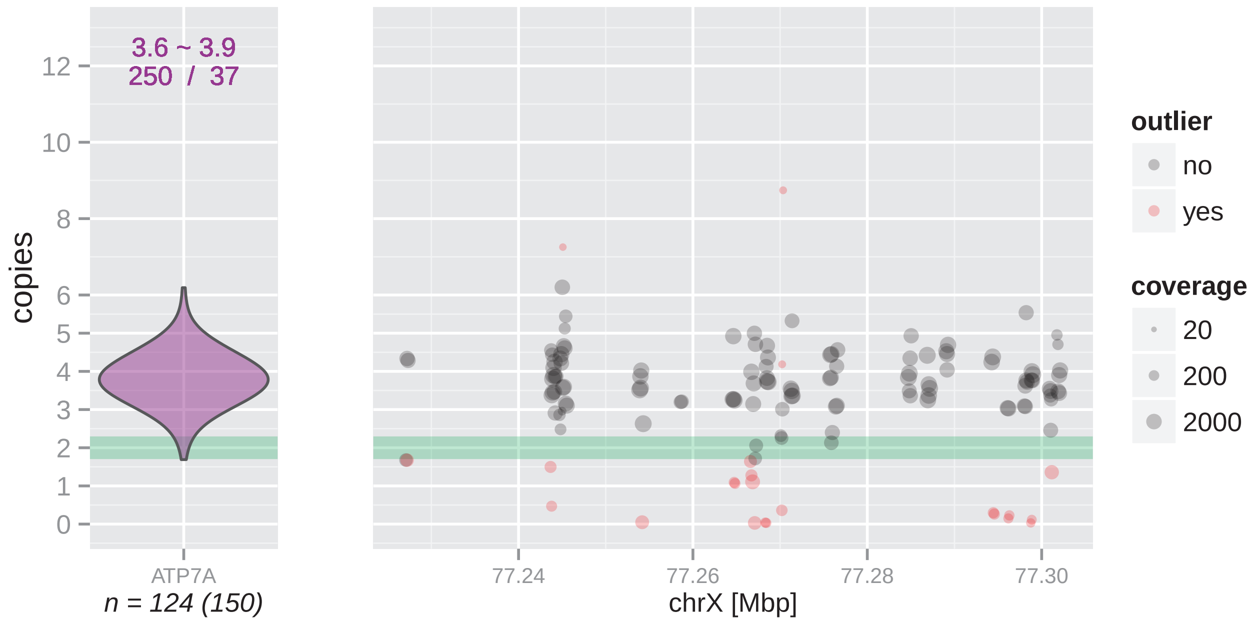
3.6 ~ 3.9
250 / 37
copies
12
10
8
6
5
4
3
2
1
0
ATP7A
n = 124 (150)
77.24
77.26
77.28
77.30
chrX [Mbp]
outlier
no
yes
coverage
20
200
2000

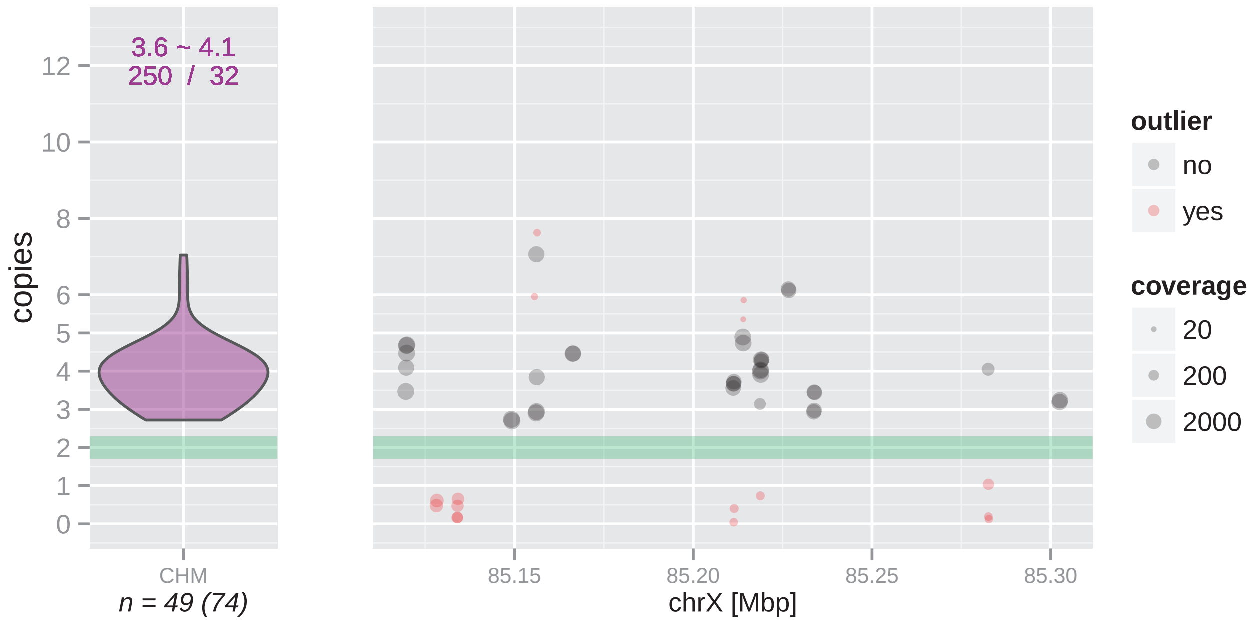
3.6 ~ 4.1
250 / 32
copies
12
10
8
6
5
4
3
2
1
0
CHM
n = 49 (74)
85.15
85.20
85.25
85.30
chrX [Mbp]
outlier
no
yes
coverage
20
200
2000

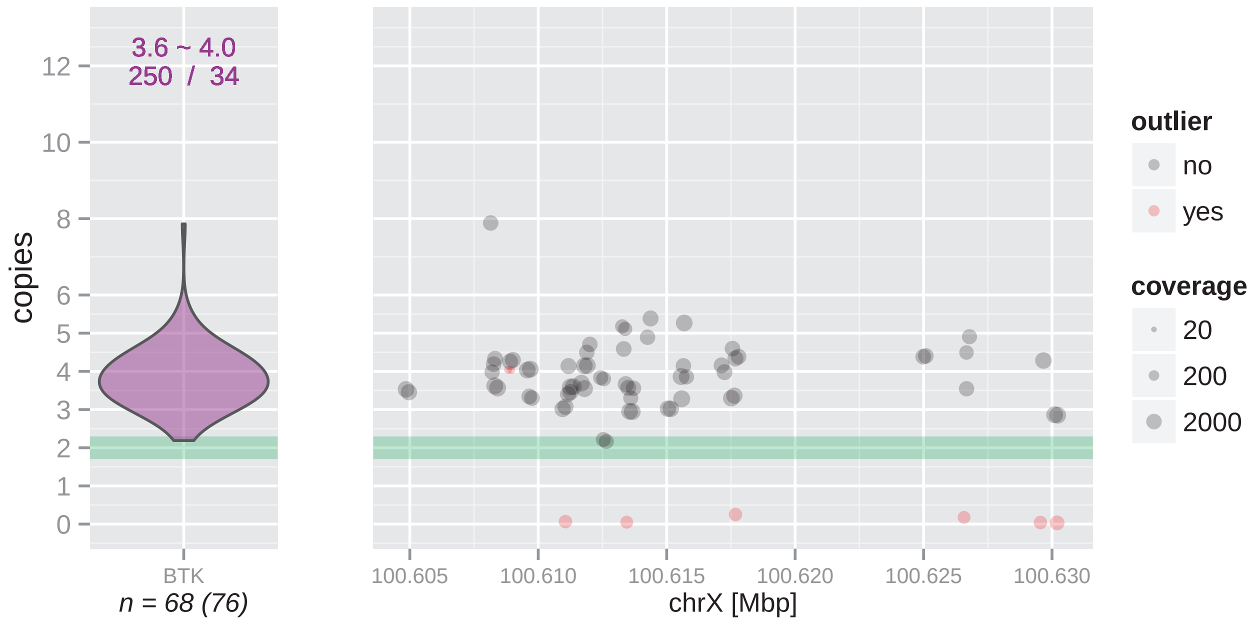
3.6 ~ 4.0
250 / 34
copies
12
10
8
6
5
4
3
2
1
0
BTK
n = 68 (76)
100.605
100.610
100.615
100.620
100.625
100.630
chrX [Mbp]
outlier
no
yes
coverage
20
200
2000
