# Supplementary material for: Quantitative analysis of differences in copy numbers using read depth obtained from PCR-enriched samples and controls
Source: BMC Bioinformatics. 2015 Jan 28;16:17. doi: 10.1186/s12859-014-0428-5 (PMC4384318; doi:10.1186/s12859-014-0428-5)
Supplement: Additional file 4 — Comparison with ONCOCNV. This archive (zip) contains all output-files generated for the comparison of quandico and ONCOCNV. [file 12859_2014_428_MOESM4_ESM.zip › quandico/M63_S2_NA01416_M62M63_SC.cov.pdf]

QIAGEN GeneRead Copy Number Analysis

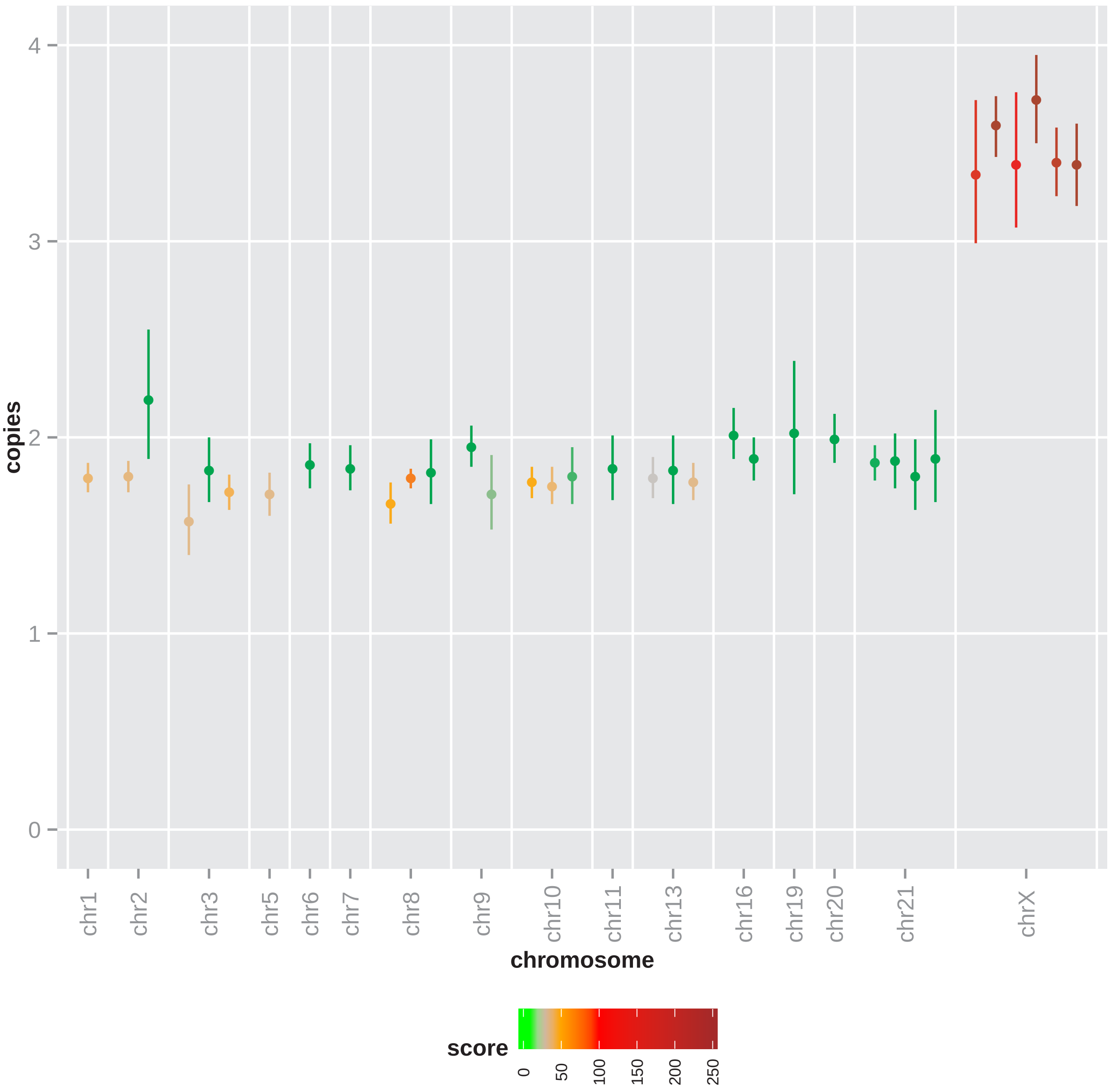

QIAGEN GeneRead Copy Number Analysis

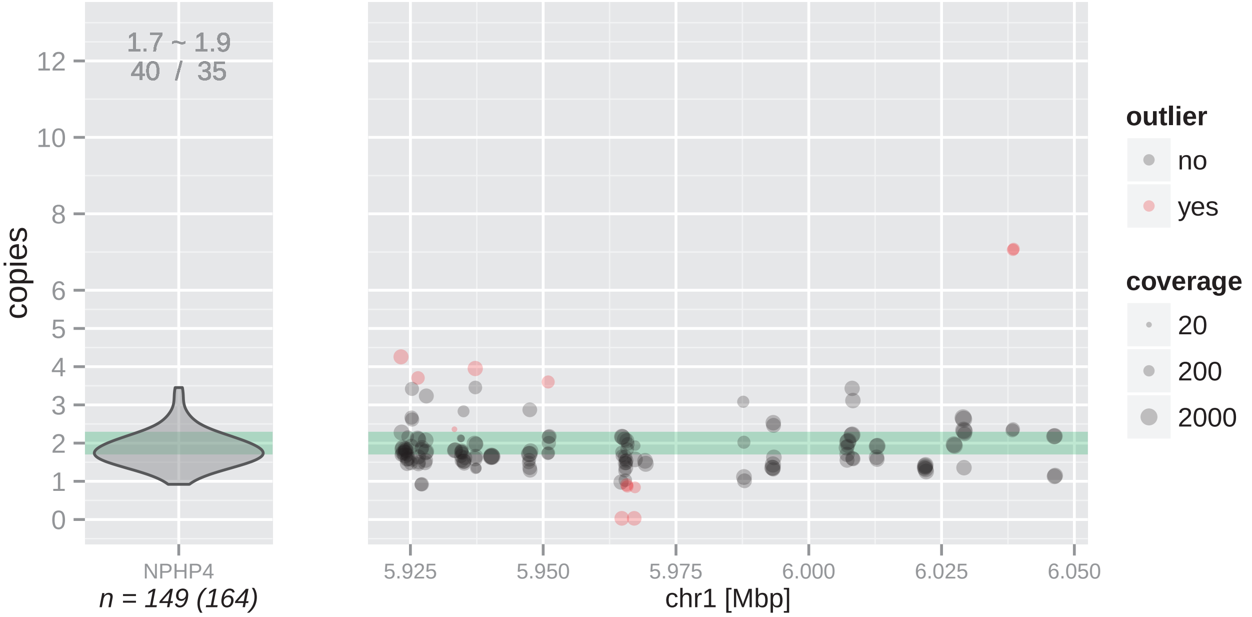

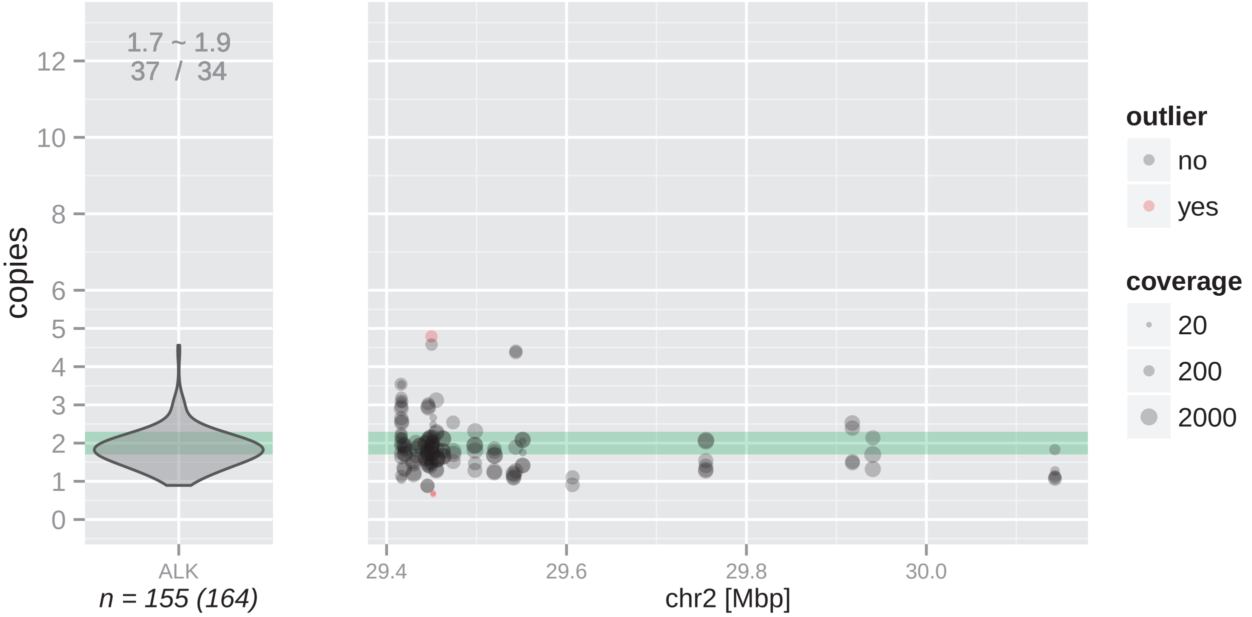

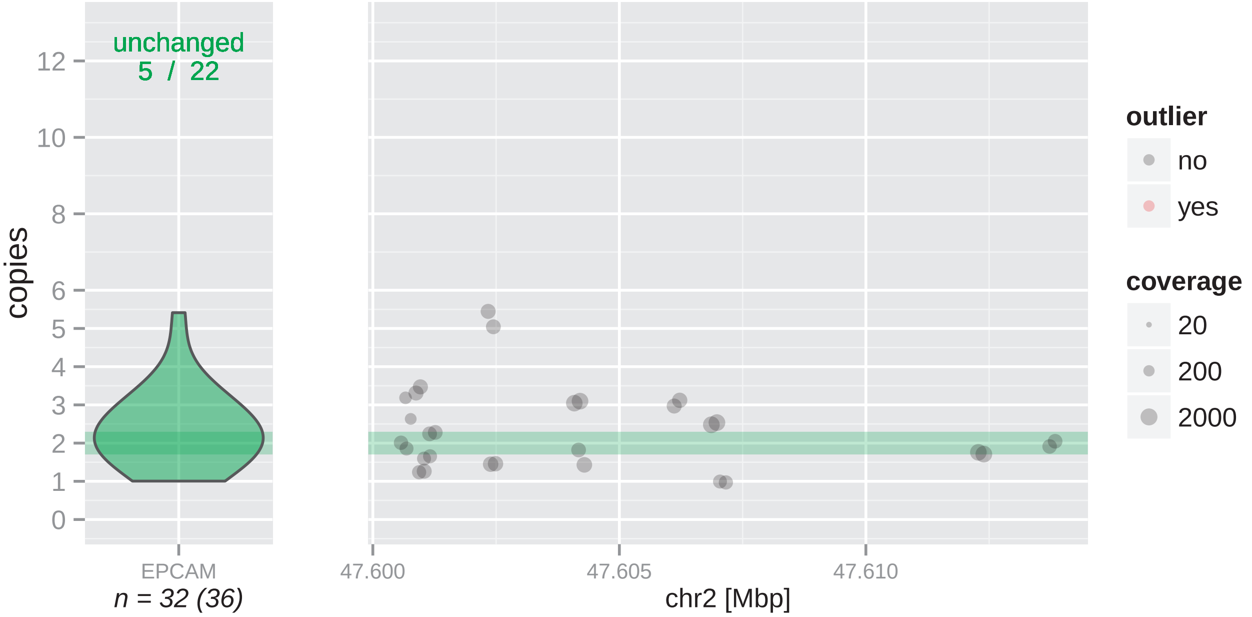
unchanged
5 / 22
copies
12
10
8
6
5
4
3
2
1
0
EPCAM
n = 32 (36)
47.600
47.605
47.610
chr2 [Mbp]
outlier
no
yes
coverage
20
200
2000

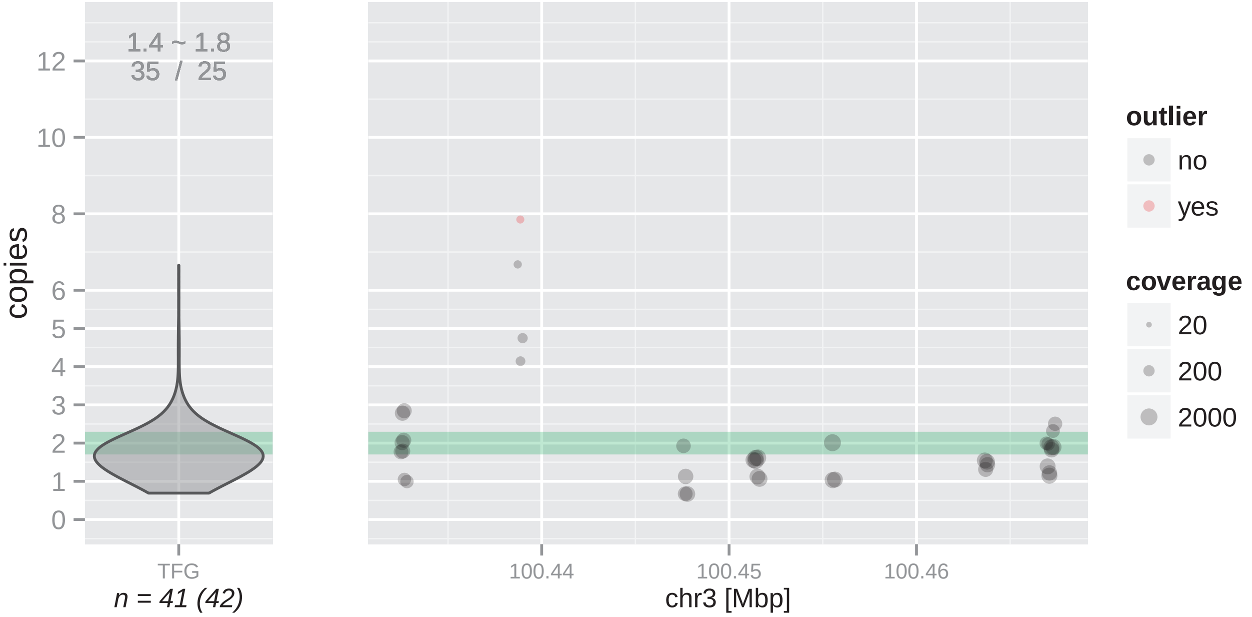
1.4 ~ 1.8
35 / 25
copies
12
10
8
6
5
4
3
2
1
0
TFG
n = 41 (42)
100.44
100.45
100.46
chr3 [Mbp]
outlier
no
yes
coverage
20
200
2000

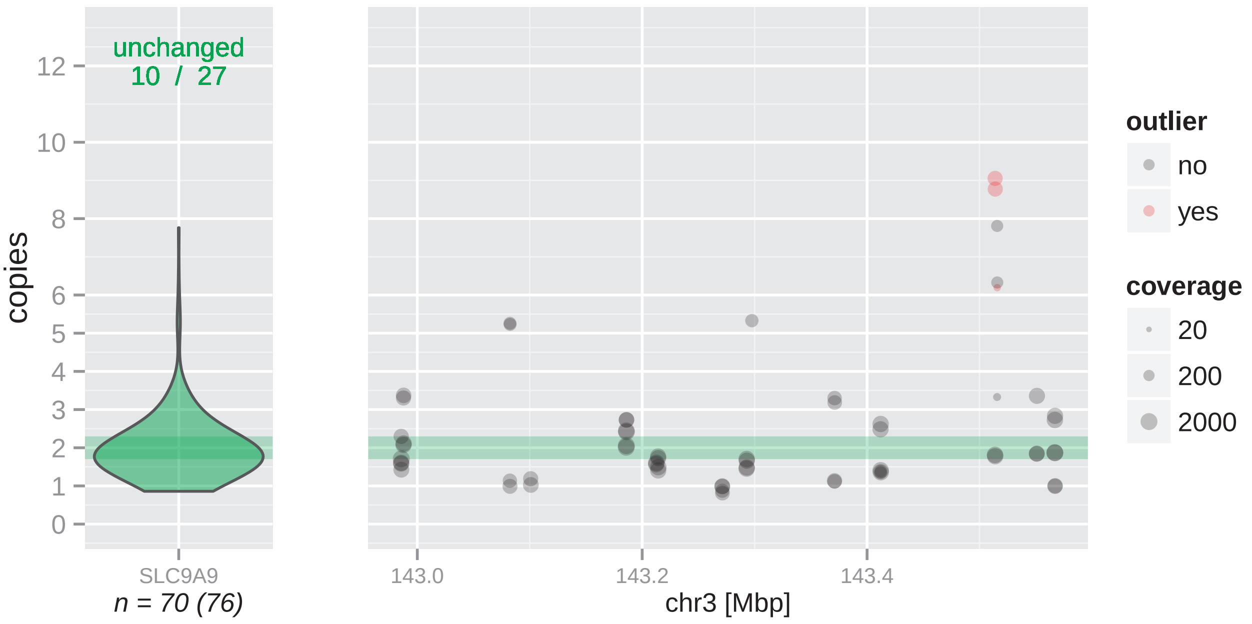
unchanged
10 / 27
copies
12
10
8
6
5
4
3
2
1
0
SLC9A9
n = 70 (76)
143.0
143.2
143.4
chr3 [Mbp]
outlier
no
yes
coverage
20
200
2000

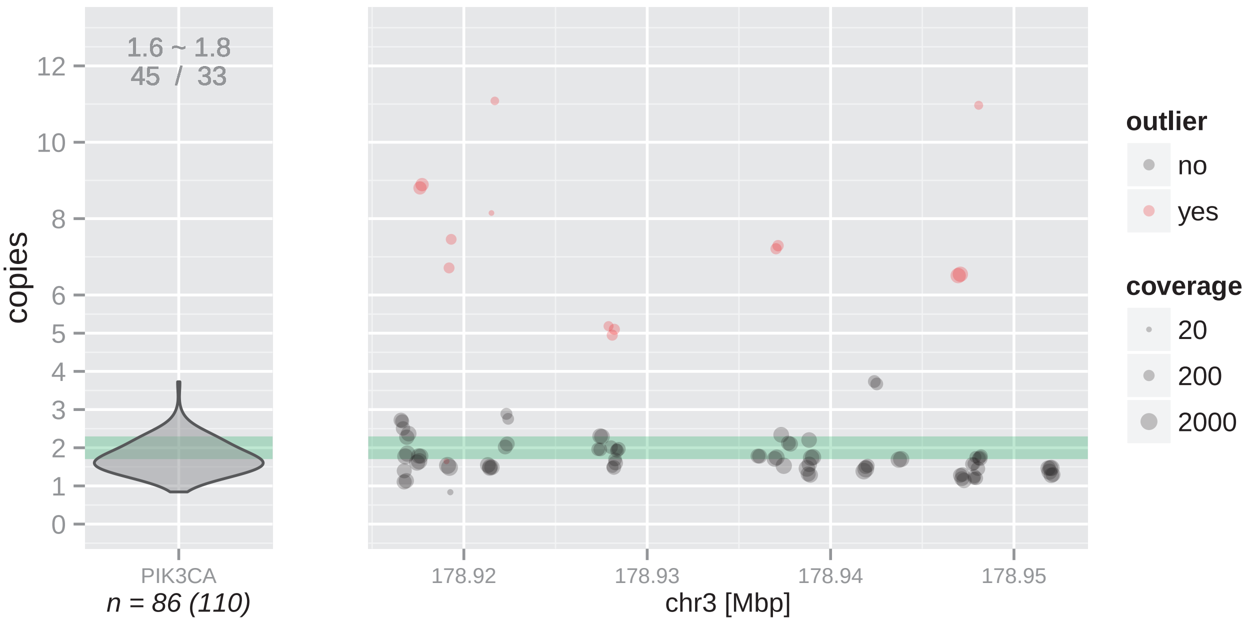
1.6 ~ 1.8
45 / 33
copies
12
10
8
6
5
4
3
2
1
0
PIK3CA
n = 86 (110)
178.92
178.93
178.94
178.95
chr3 [Mbp]
outlier
no
yes
coverage
20
200
2000

QIAGEN®

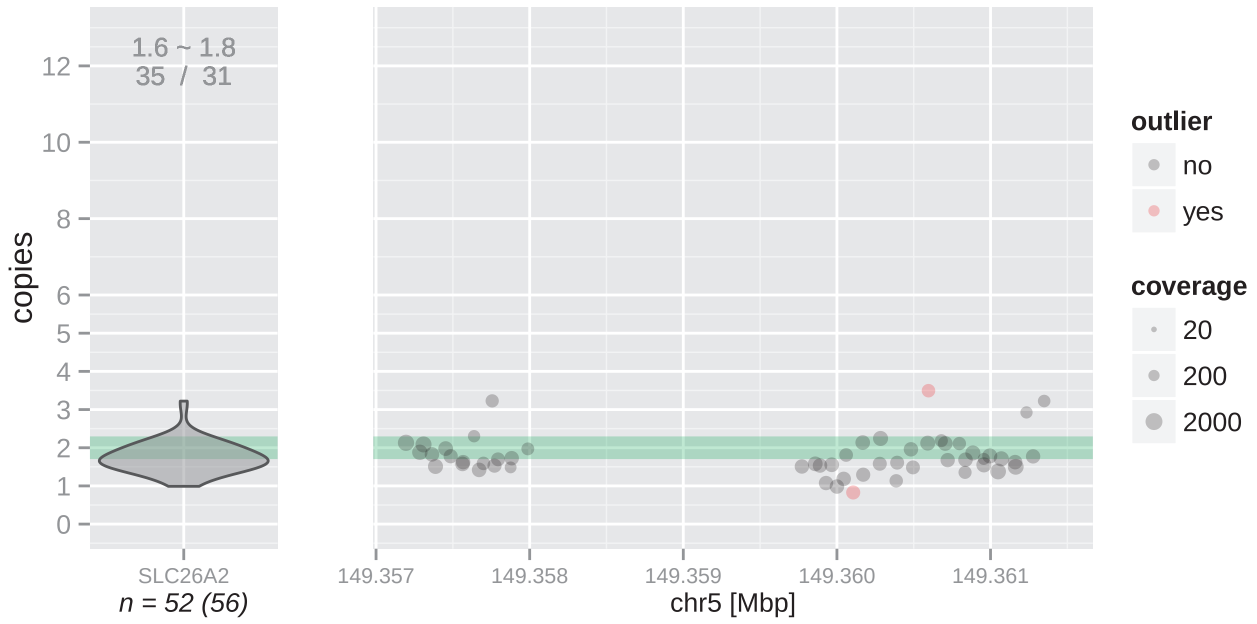

1.6 ~ 1.8
35 / 31
copies
12
10
8
6
5
4
3
2
1
0
SLC26A2
n = 52 (56)
149.357
149.358
149.359
149.360
149.361
chr5 [Mbp]
outlier
no
yes
coverage
20
200
2000

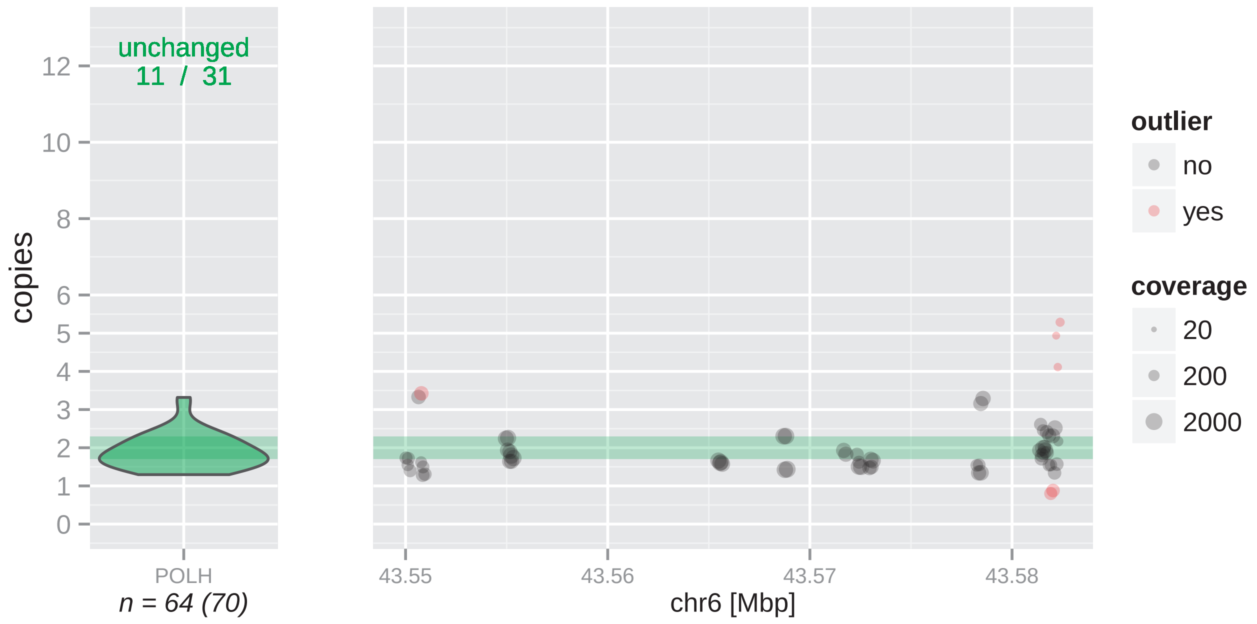

unchanged
11 / 31
copies
12
10
8
6
5
4
3
2
1
0
POLH
n = 64 (70)
43.55
43.56
43.57
43.58
chr6 [Mbp]
outlier
no
yes
coverage
20
200
2000

QIAGEN®

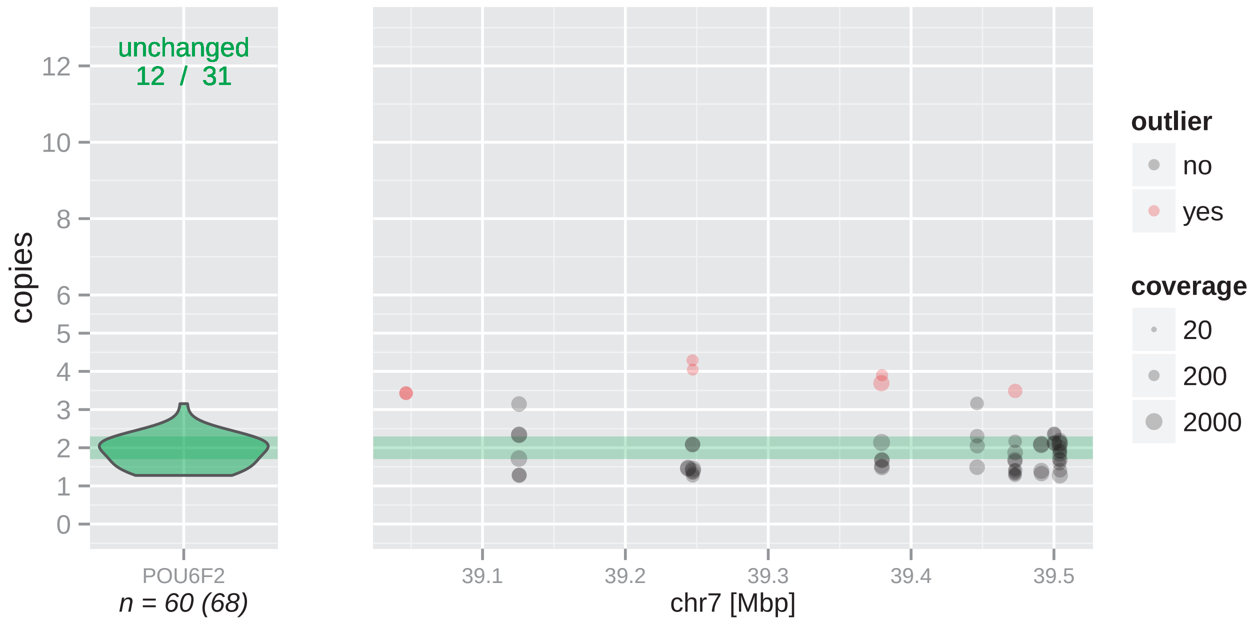
unchanged
12 / 31
copies
12
10
8
6
5
4
3
2
1
0
POU6F2
n = 60 (68)
39.1
39.2
39.3
39.4
39.5
chr7 [Mbp]
outlier
no
yes
coverage
20
200
2000

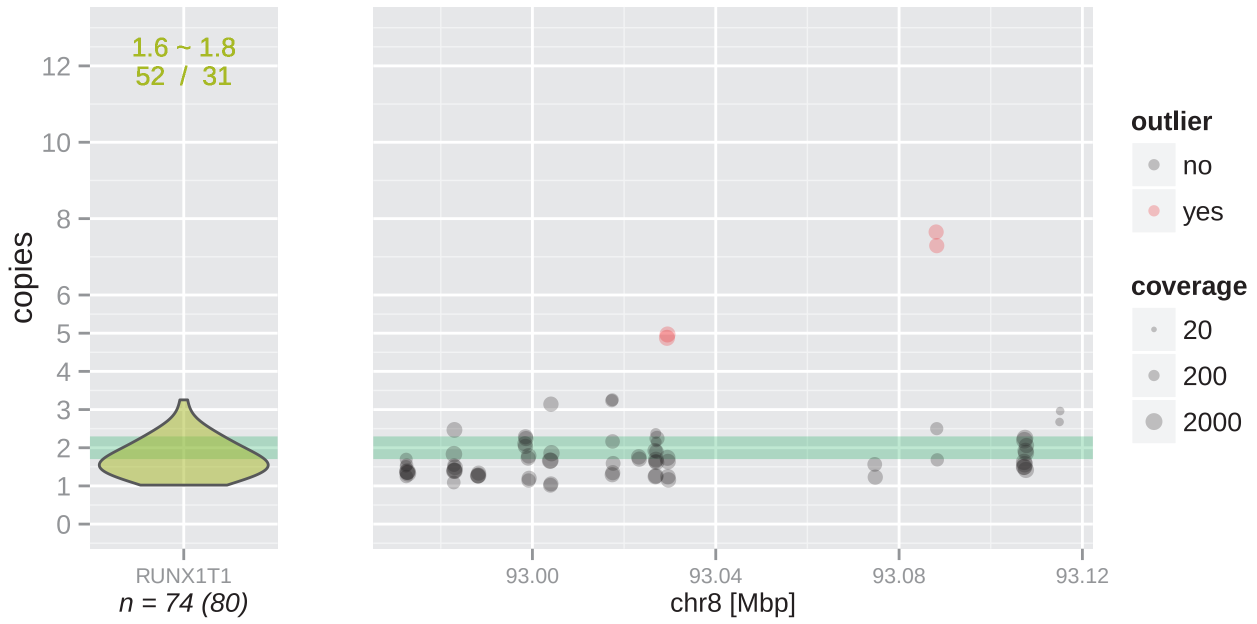
1.6 ~ 1.8
52 / 31
copies
12
10
8
6
5
4
3
2
1
0
RUNX1T1
n = 74 (80)
93.00
93.04
93.08
93.12
chr8 [Mbp]
outlier
no
yes
coverage
20
200
2000

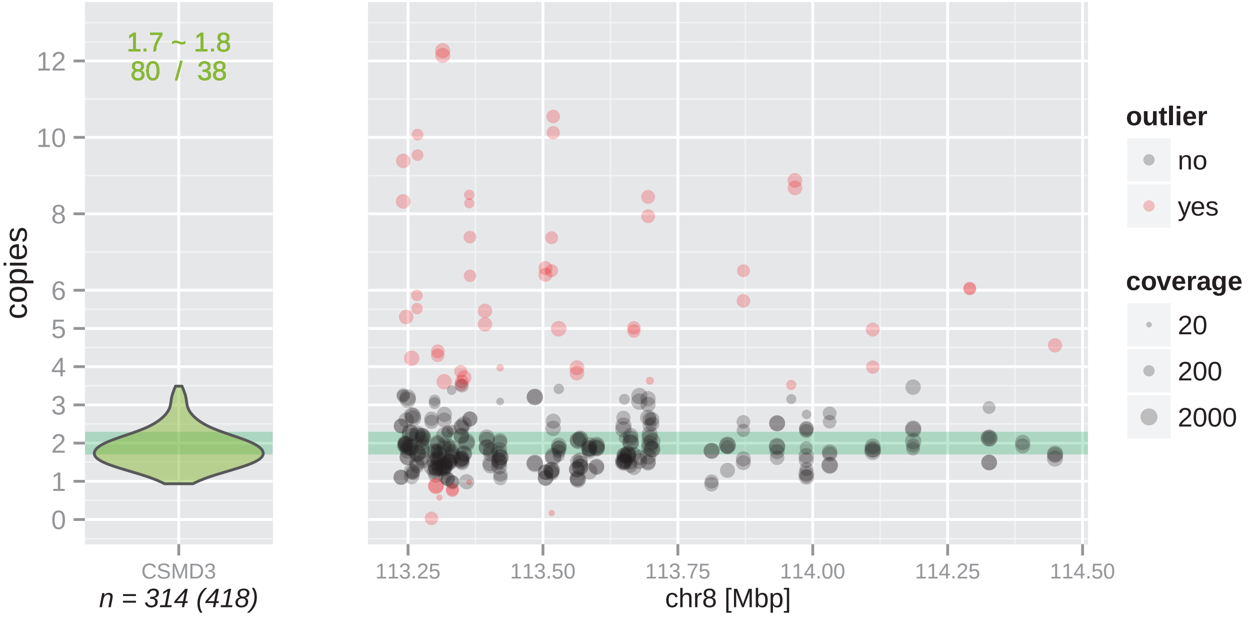
1.7 ~ 1.8
80 / 38
copies
12
10
8
6
5
4
3
2
1
0
CSMD3
n = 314 (418)
113.25
113.50
113.75
114.00
114.25
114.50
chr8 [Mbp]
outlier
no
yes
coverage
20
200
2000

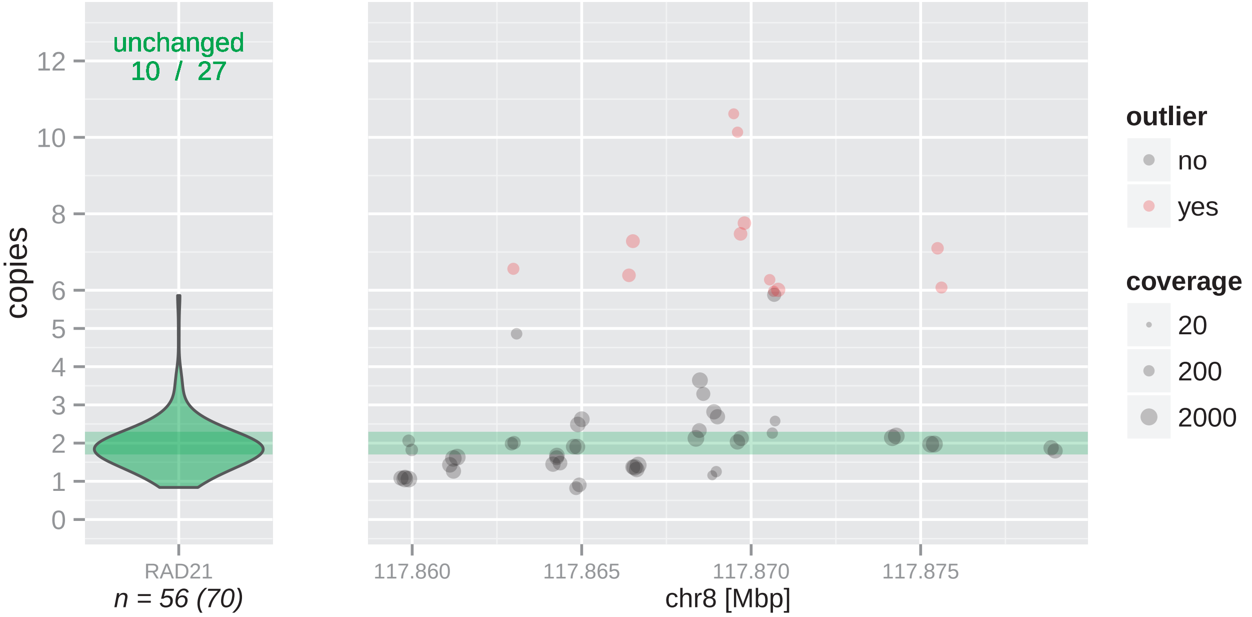
unchanged
10 / 27
copies
12
10
8
6
5
4
3
2
1
0
RAD21
n = 56 (70)
117.860
117.865
117.870
117.875
chr8 [Mbp]
outlier
no
yes
coverage
20
200
2000

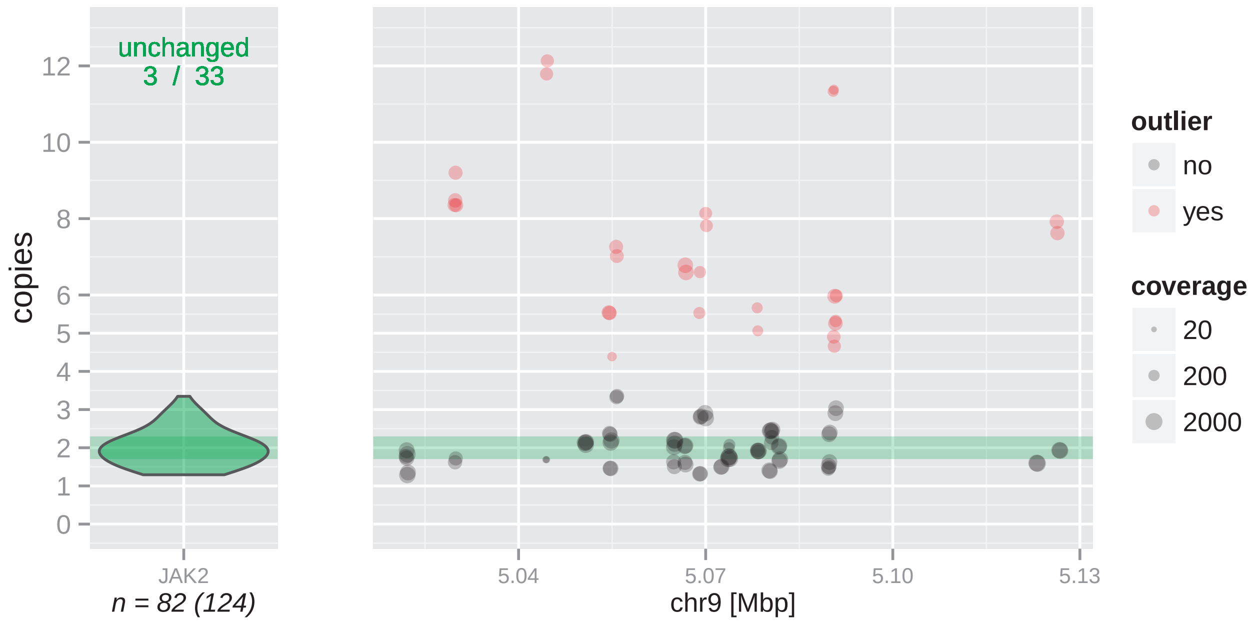
unchanged
3 / 33
copies
12
10
8
6
5
4
3
2
1
0
JAK2
n = 82 (124)
5.04
5.07
5.10
5.13
chr9 [Mbp]
outlier
no
yes
coverage
20
200
2000

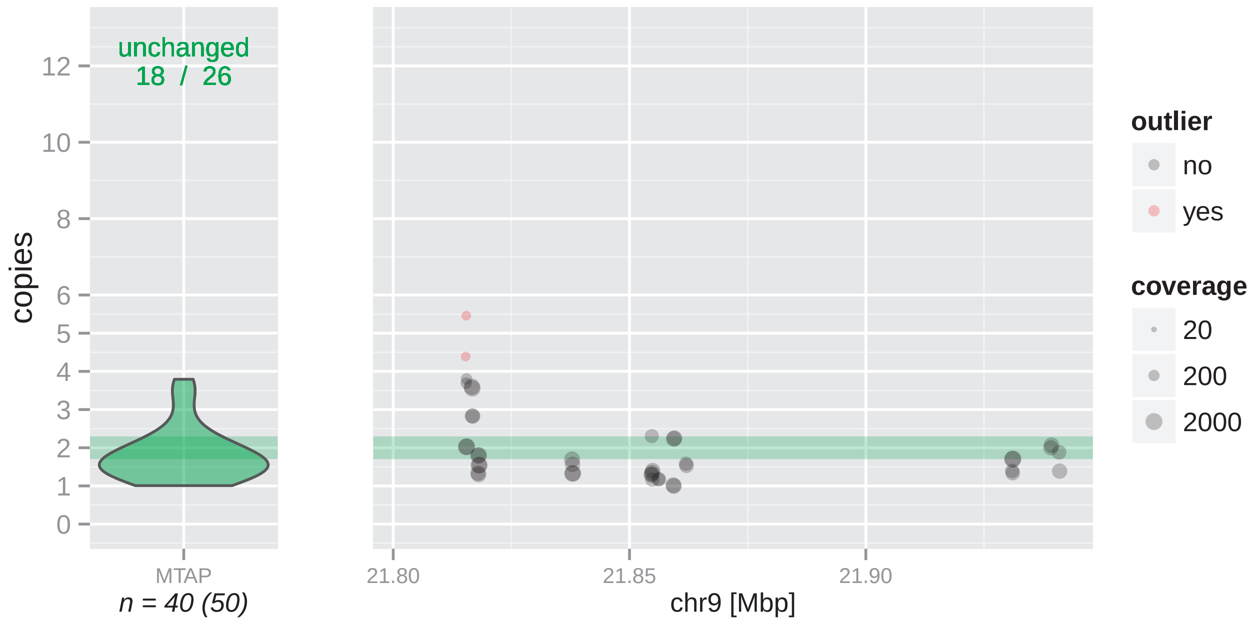
unchanged
18 / 26
copies
12
10
8
6
5
4
3
2
1
0
MTAP
n = 40 (50)
21.80
21.85
21.90
chr9 [Mbp]
outlier
no
yes
coverage
20
200
2000

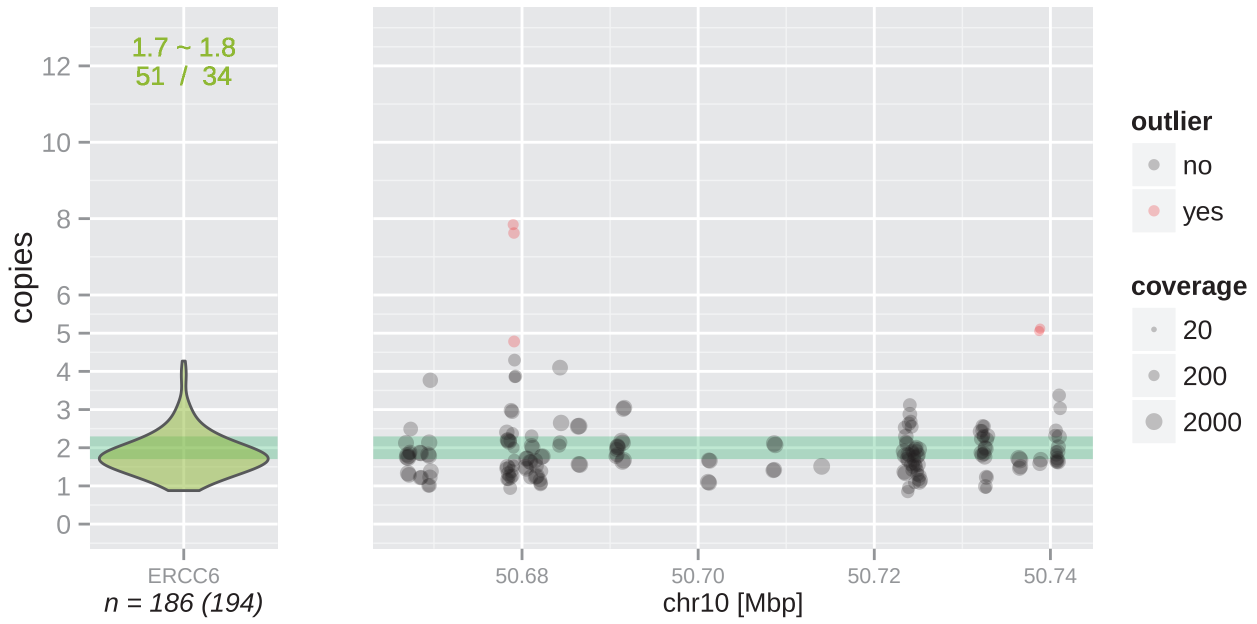
1.7 ~ 1.8
51 / 34
copies
12
10
8
6
5
4
3
2
1
0
ERCC6
n = 186 (194)
50.68
50.70
50.72
50.74
chr10 [Mbp]
outlier
no
yes
coverage
20
200
2000

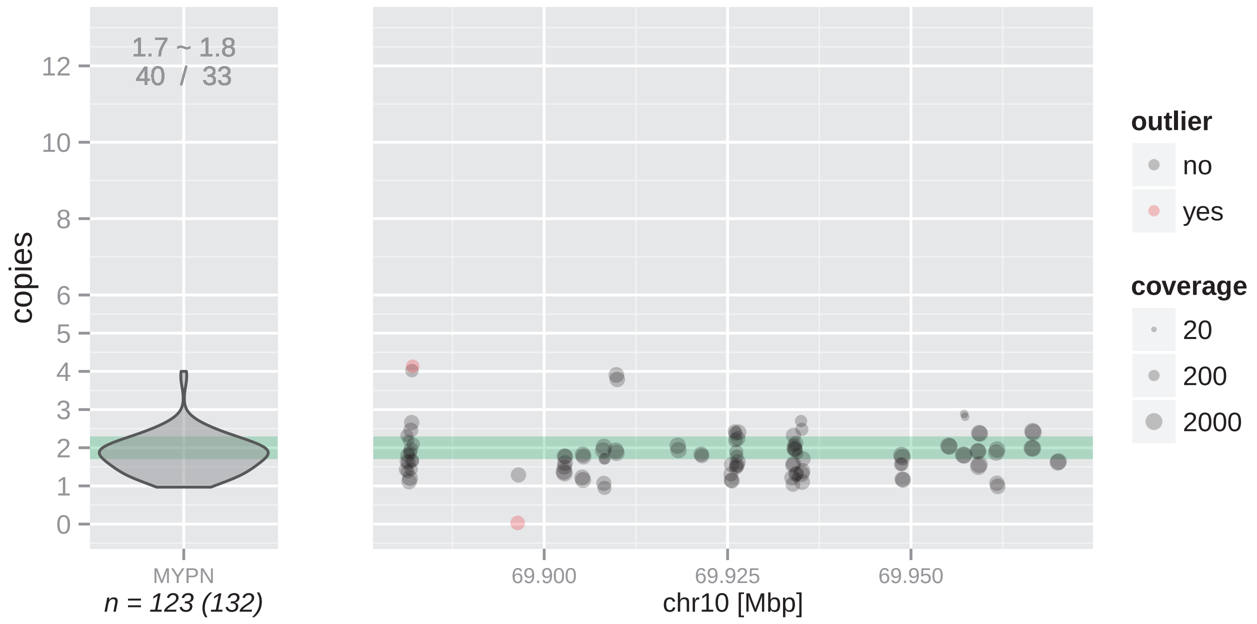
1.7 ~ 1.8
40 / 33
copies
12
10
8
6
5
4
3
2
1
0
MYPN
n = 123 (132)
69.900
69.925
69.950
chr10 [Mbp]
outlier
no
yes
coverage
20
200
2000

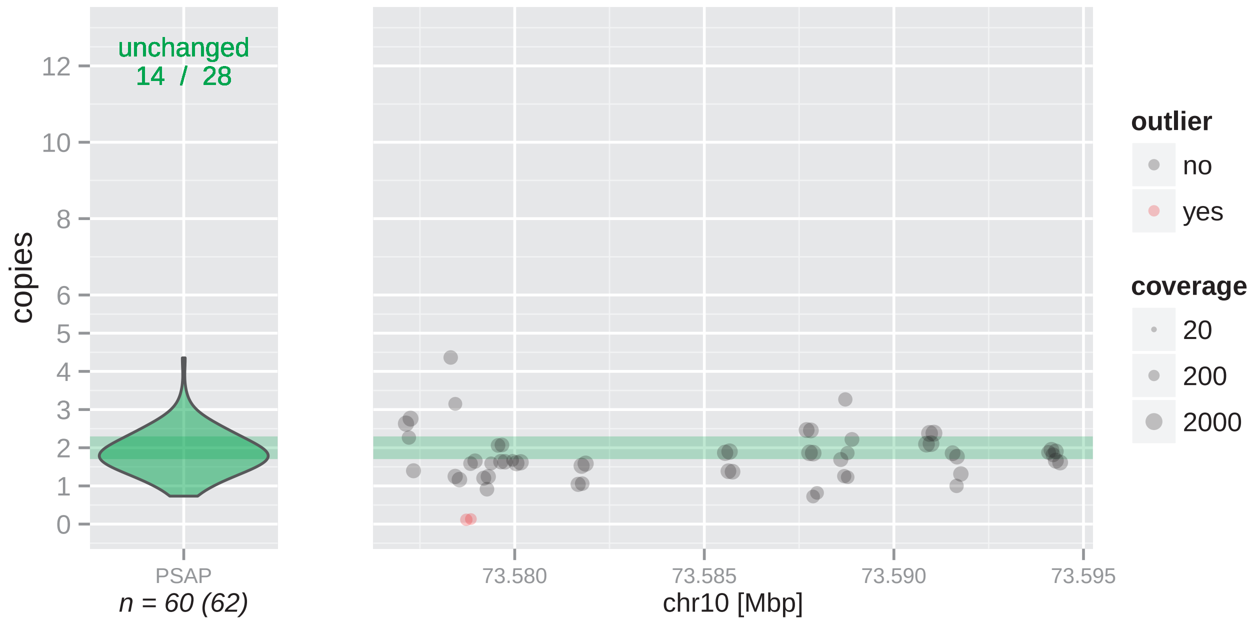
unchanged
14 / 28
copies
12
10
8
6
5
4
3
2
1
0
PSAP
n = 60 (62)
73.580
73.585
73.590
73.595
chr10 [Mbp]
outlier
no
yes
coverage
20
200
2000

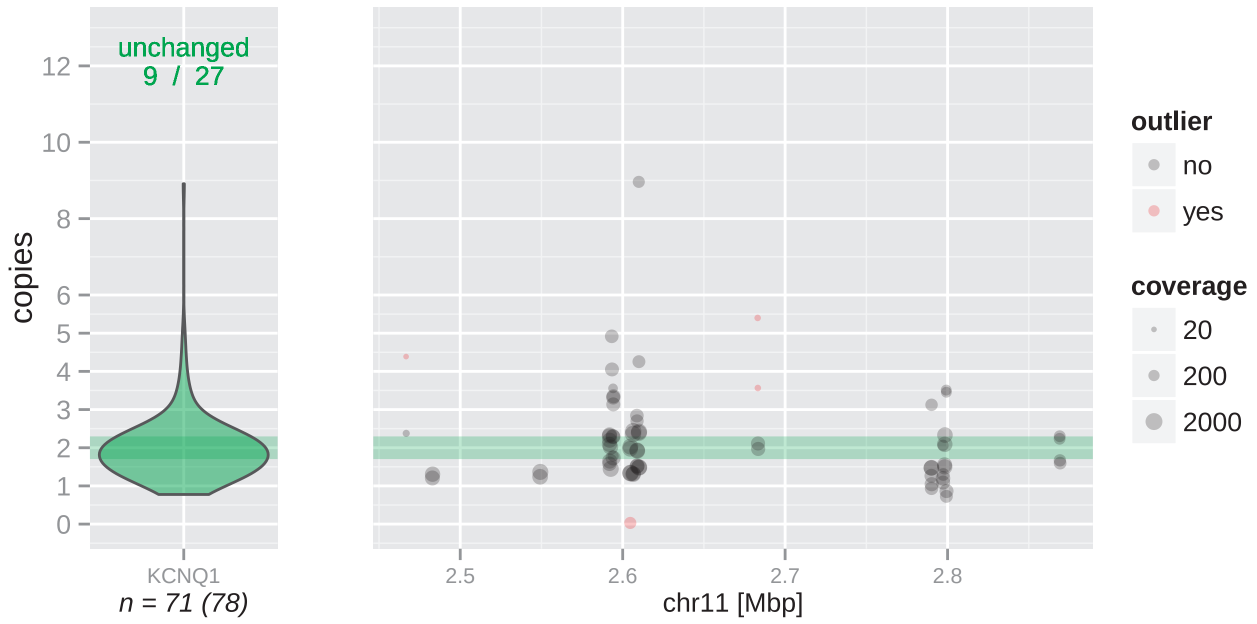
unchanged
9 / 27
copies
12
10
8
6
5
4
3
2
1
0
KCNQ1
n = 71 (78)
2.5
2.6
2.7
2.8
chr11 [Mbp]
outlier
no
yes
coverage
20
200
2000

QIAGEN GeneRead Copy Number Analysis

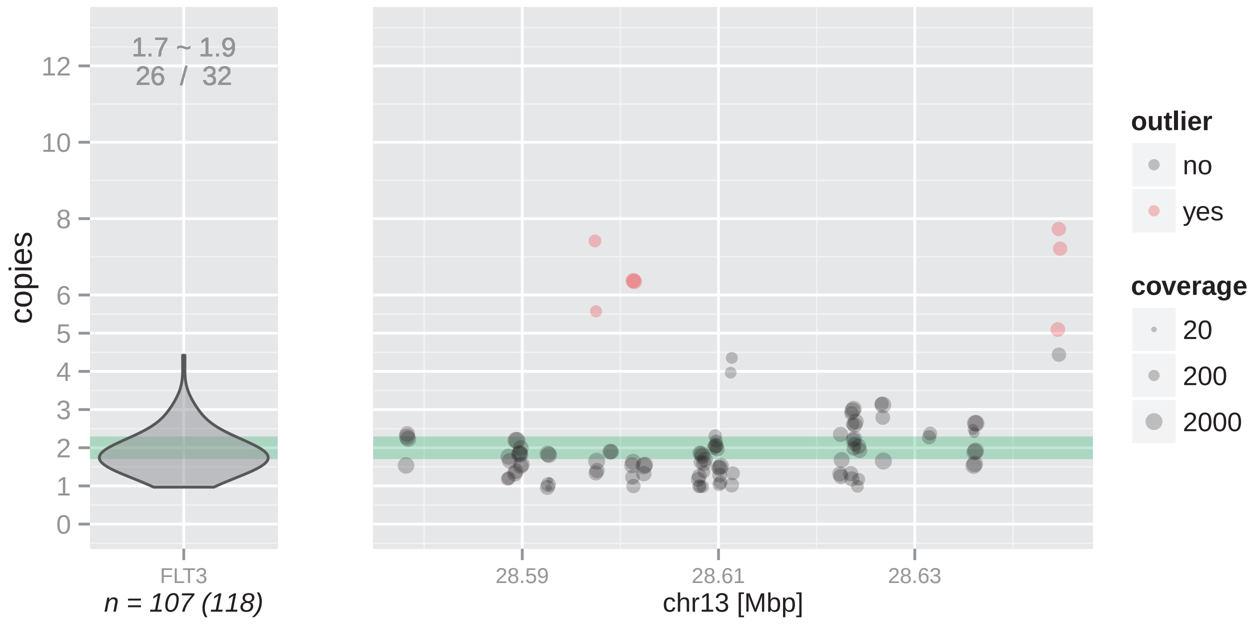

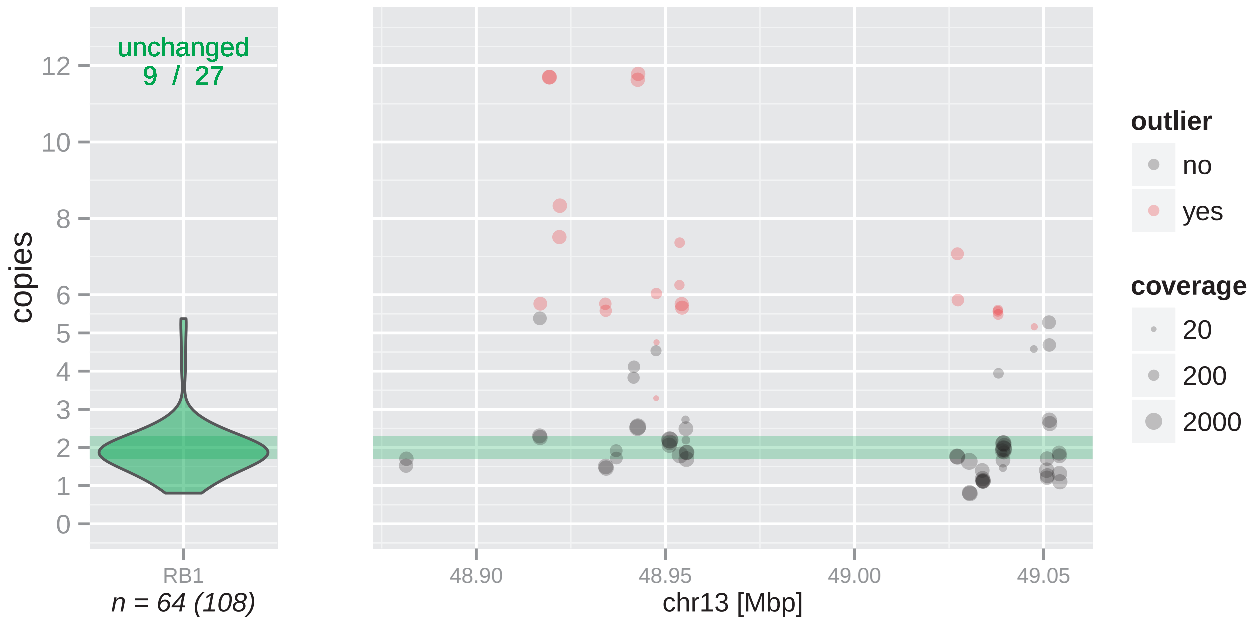

QIAGEN®

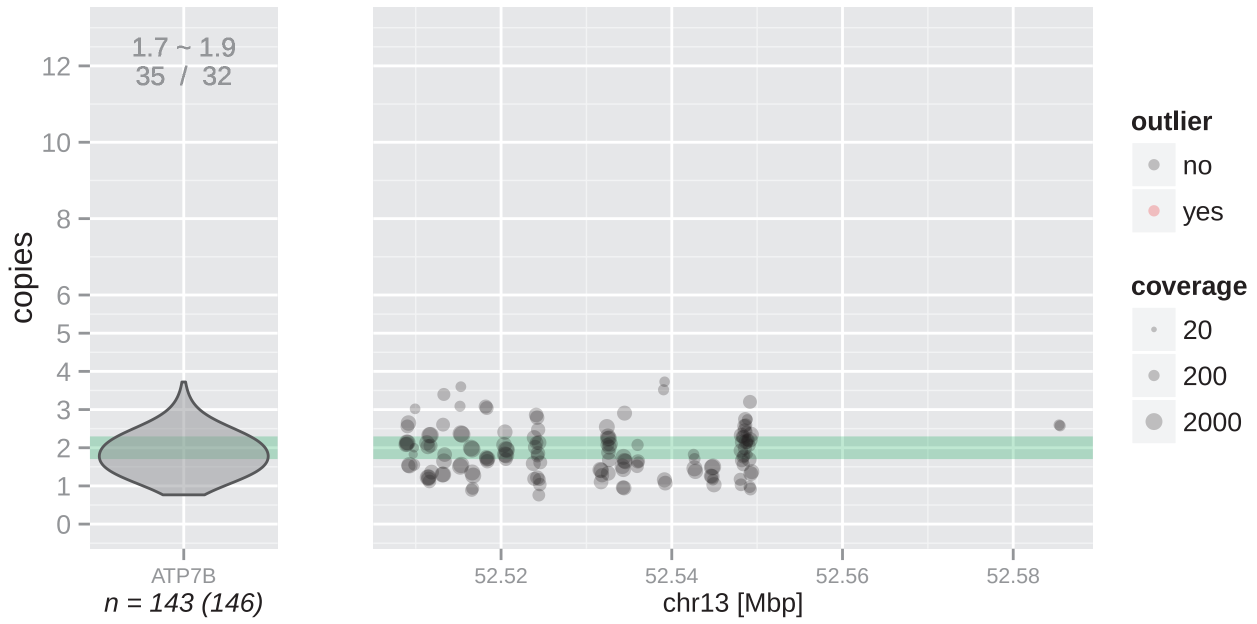
1.7 ~ 1.9
35 / 32
copies
12
10
8
6
5
4
3
2
1
0
ATP7B
n = 143 (146)
52.52
52.54
52.56
52.58
chr13 [Mbp]
outlier
no
yes
coverage
20
200
2000

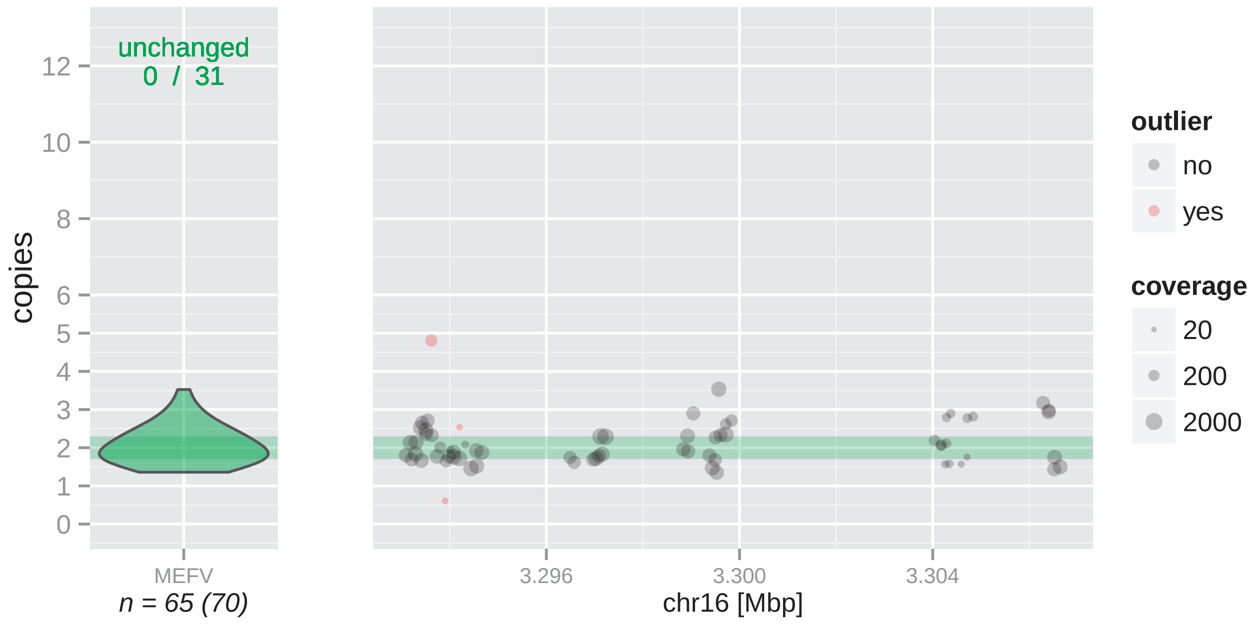
unchanged
0 / 31
copies
12
10
8
6
5
4
3
2
1
0
MEFV
n = 65 (70)
3.296
3.300
3.304
chr16 [Mbp]
outlier
no
yes
coverage
20
200
2000

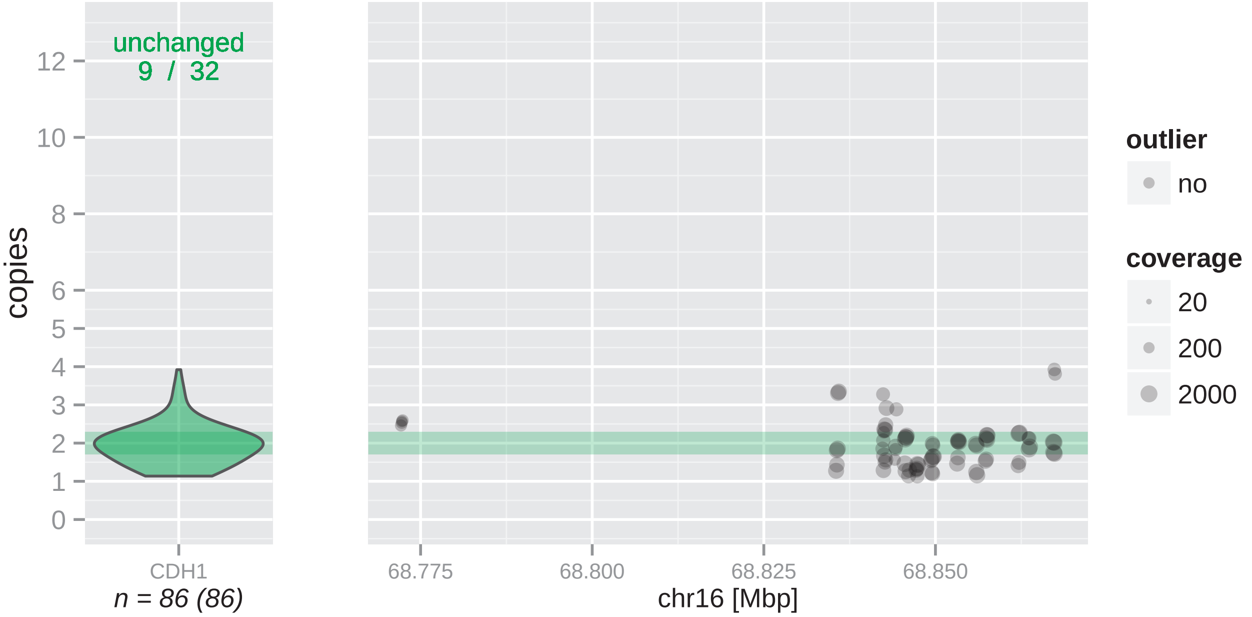
unchanged
9 / 32
copies
12
10
8
6
5
4
3
2
1
0
CDH1
n = 86 (86)
68.775
68.800
68.825
68.850
chr16 [Mbp]
outlier
no
coverage
20
200
2000

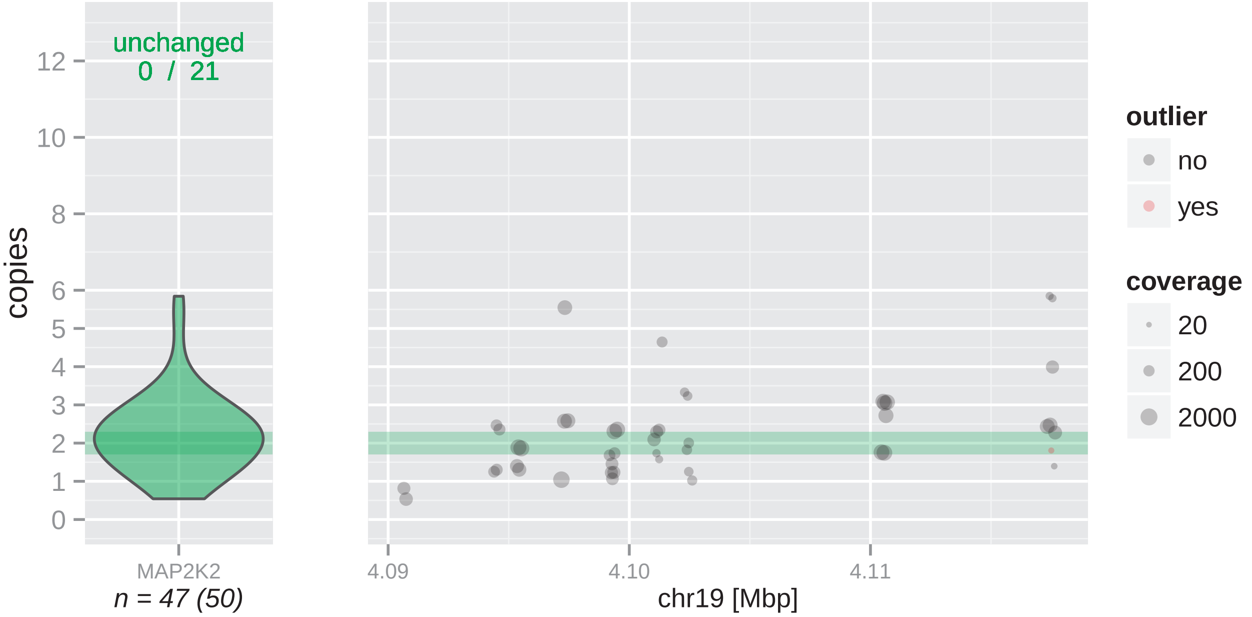
unchanged
0 / 21
copies
12
10
8
6
5
4
3
2
1
0
MAP2K2
n = 47 (50)
4.09
4.10
4.11
chr19 [Mbp]
outlier
no
yes
coverage
20
200
2000

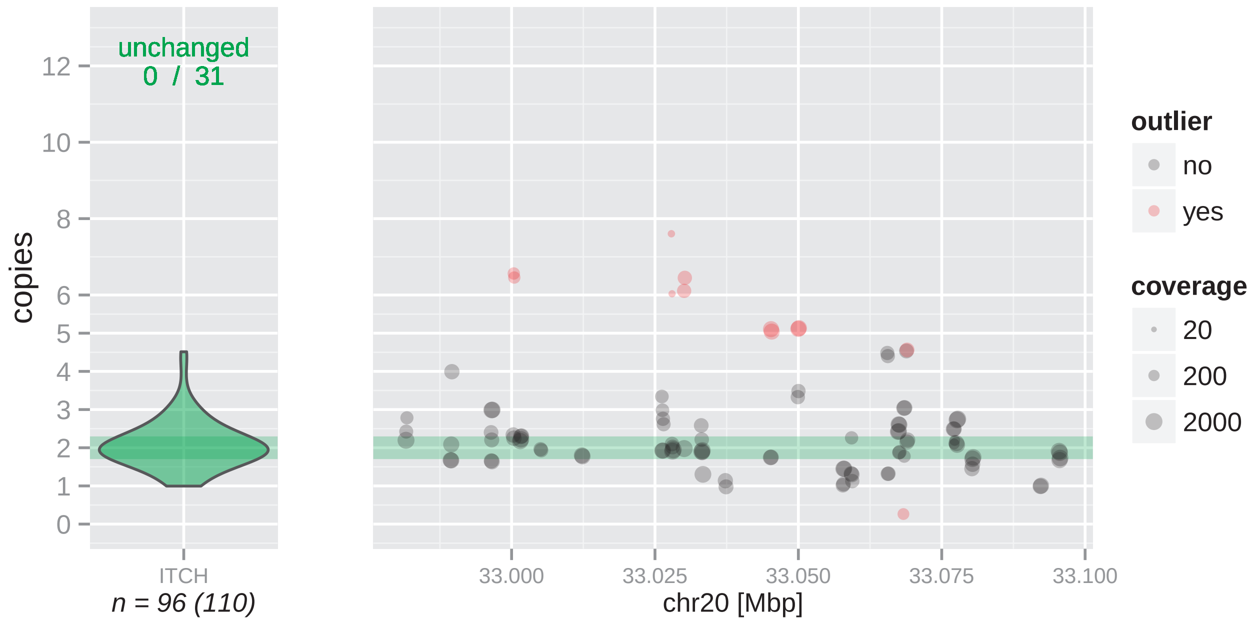

unchanged
0 / 31
copies
12
10
8
6
5
4
3
2
1
0
ITCH
n = 96 (110)
33.000
33.025
33.050
33.075
33.100
chr20 [Mbp]
outlier
no
yes
coverage
20
200
2000

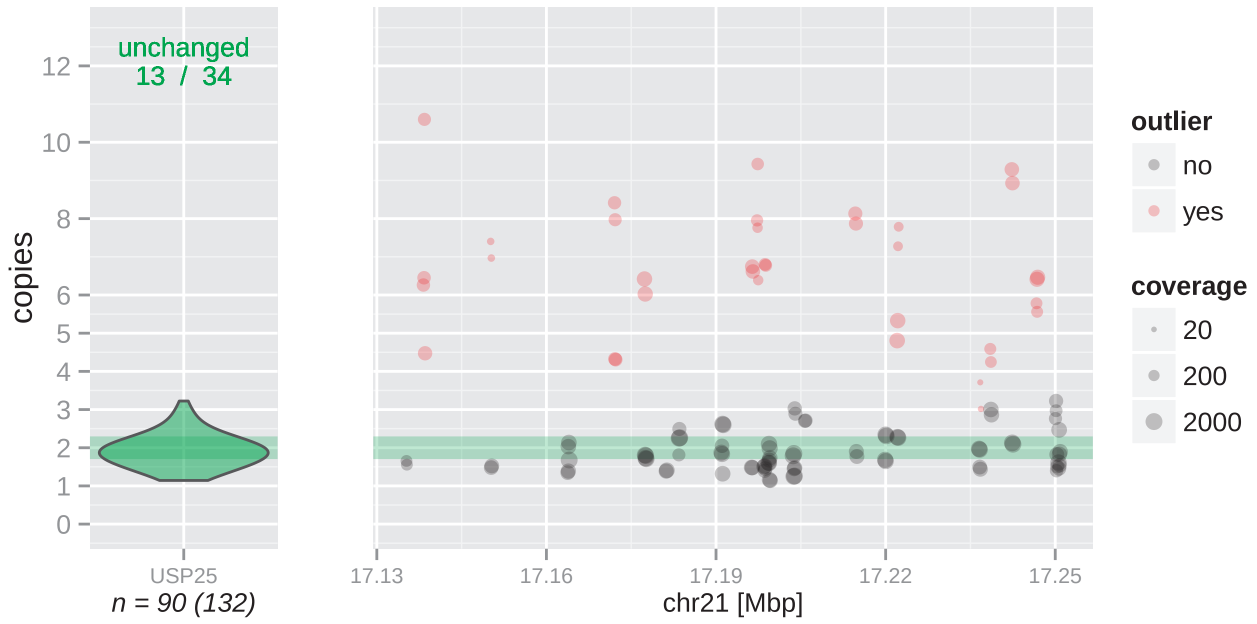

unchanged
13 / 34
copies
12
10
8
6
5
4
3
2
1
0
USP25
n = 90 (132)
17.13
17.16
17.19
17.22
17.25
chr21 [Mbp]
outlier
no
yes
coverage
20
200
2000

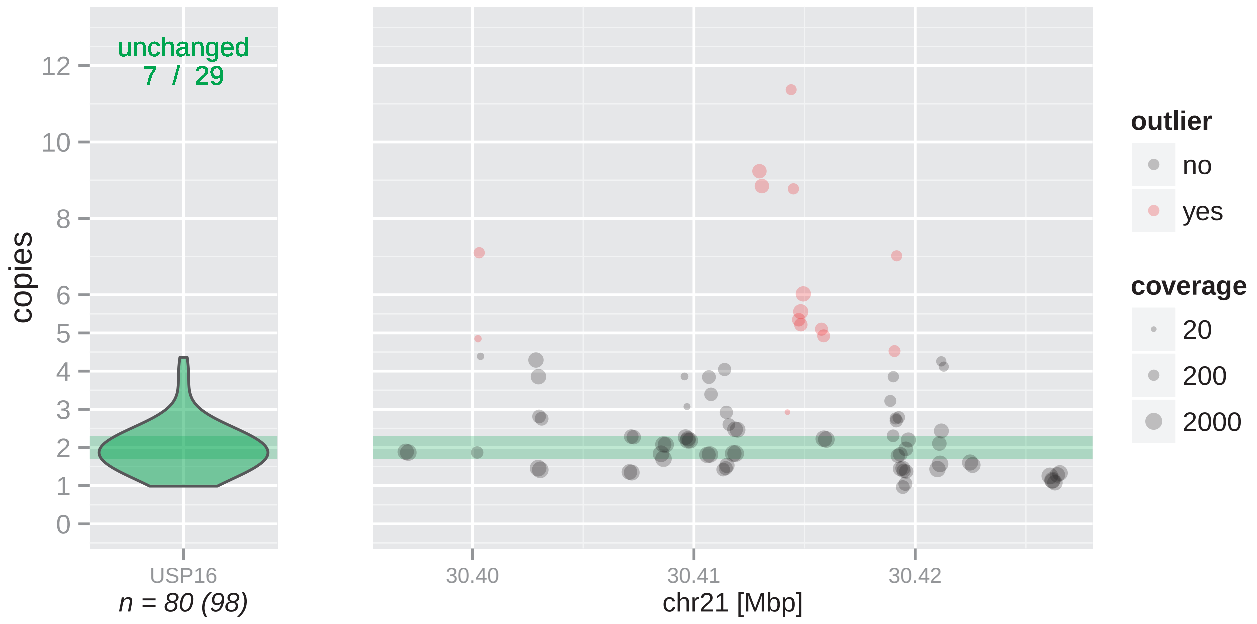
unchanged
7 / 29
copies
12
10
8
6
5
4
3
2
1
0
USP16
n = 80 (98)
30.40
30.41
30.42
chr21 [Mbp]
outlier
no
yes
coverage
20
200
2000

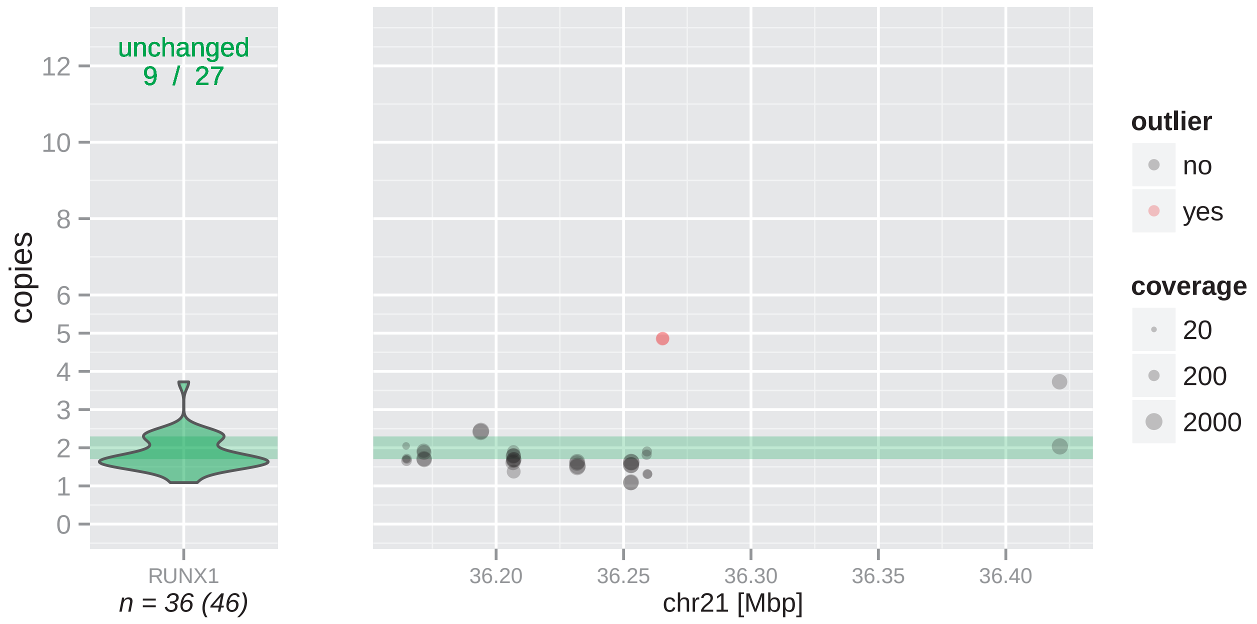
unchanged
9 / 27
copies
12
10
8
6
5
4
3
2
1
0
RUNX1
n = 36 (46)
36.20
36.25
36.30
36.35
36.40
chr21 [Mbp]
outlier
no
yes
coverage
20
200
2000

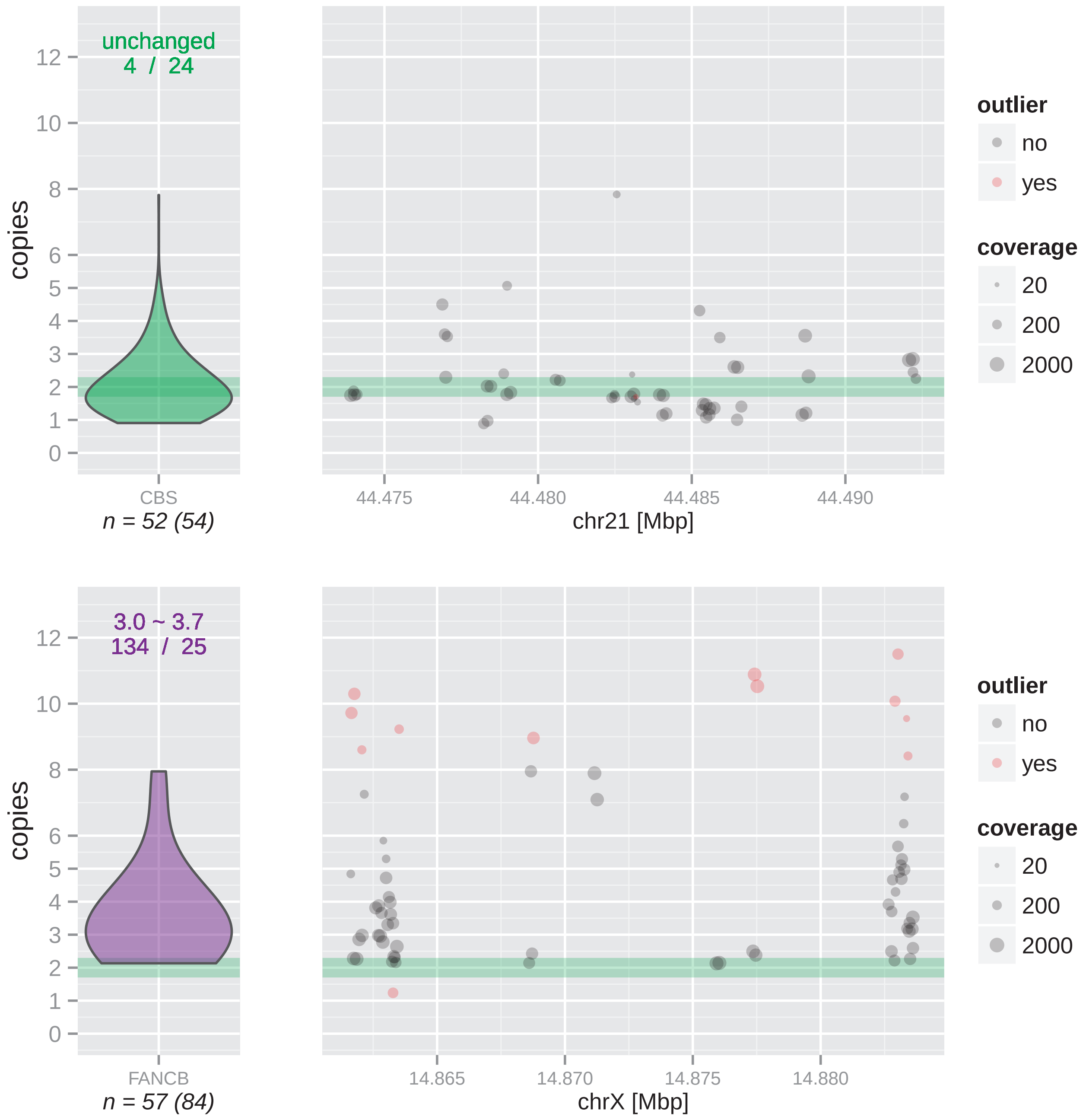
unchanged
4 / 24
copies
12
10
8
6
5
4
3
2
1
0
CBS
n = 52 (54)
44.475
44.480
44.485
44.490
chr21 [Mbp]
outlier
no
yes
coverage
20
200
2000
3.0 ~ 3.7
134 / 25
copies
FANCB
n = 57 (84)
14.865
14.870
14.875
14.880
chrX [Mbp]

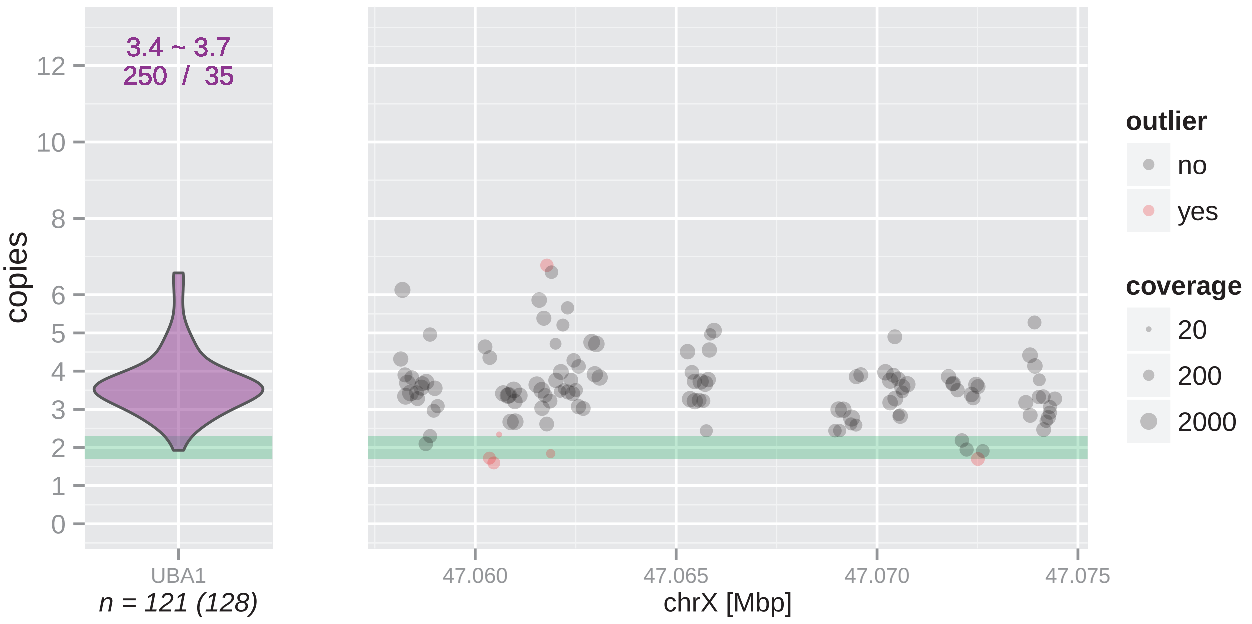
3.4 ~ 3.7
250 / 35
copies
UBA1
n = 121 (128)
47.060
47.065
47.070
47.075
chrX [Mbp]
outlier
no
yes
coverage
20
200
2000

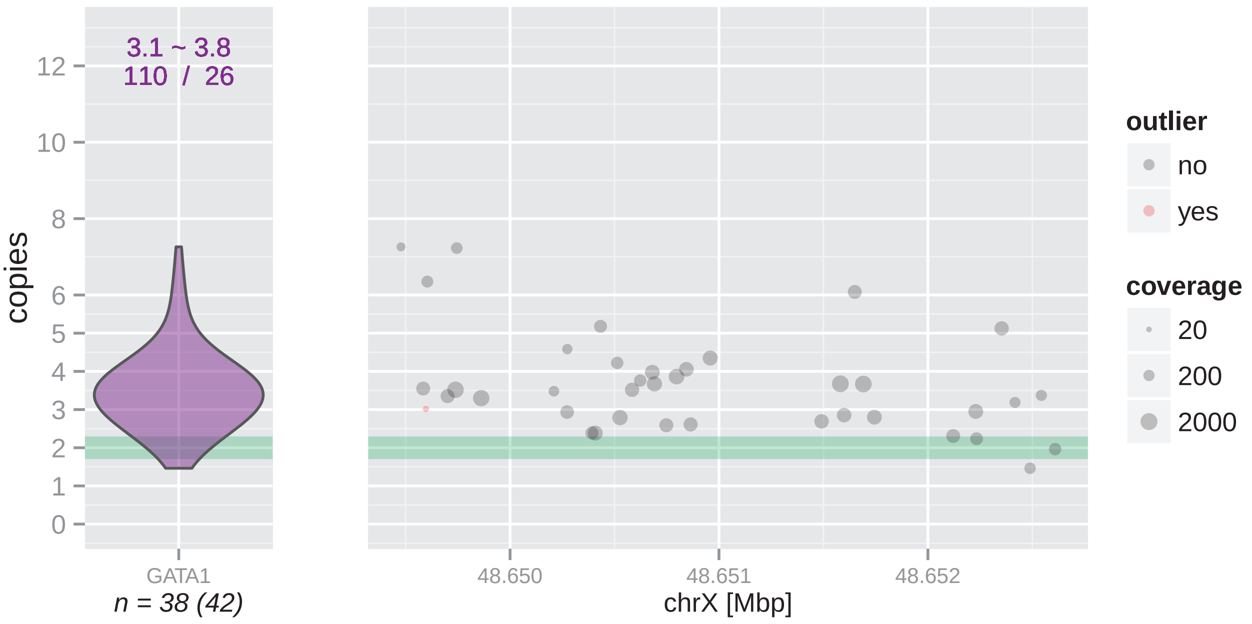
3.1 ~ 3.8
110 / 26
copies
GATA1
n = 38 (42)
48.650
48.651
48.652
chrX [Mbp]
outlier
no
yes
coverage
20
200
2000

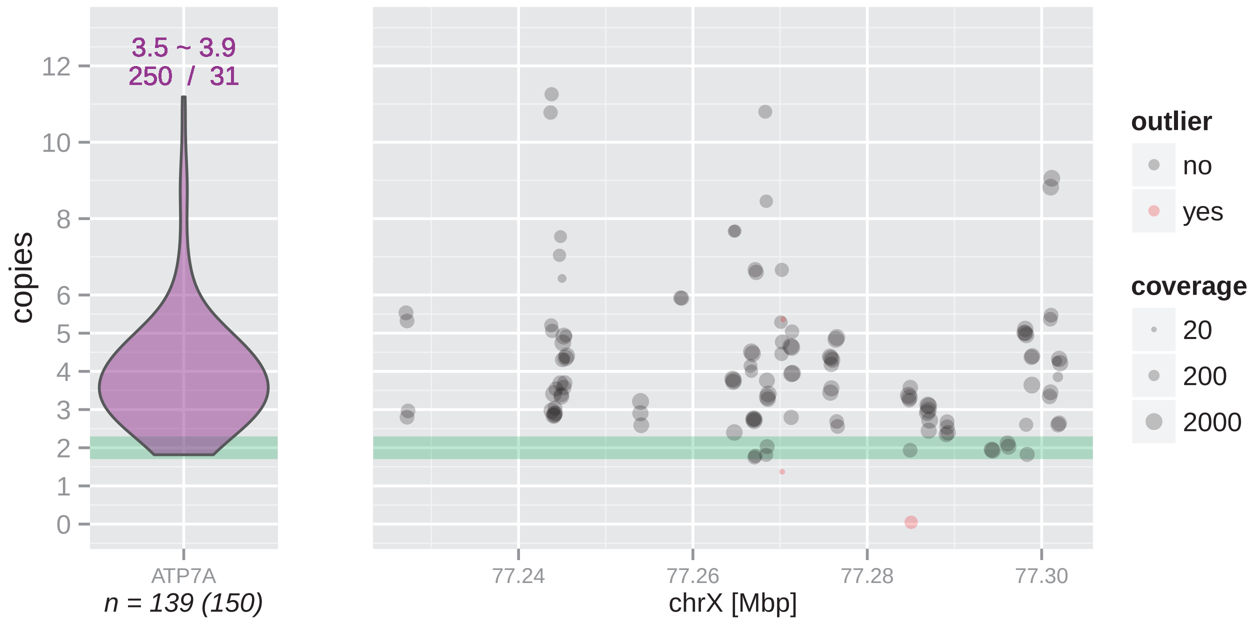
3.5 ~ 3.9
250 / 31
copies
12
10
8
6
5
4
3
2
1
0
ATP7A
n = 139 (150)
77.24
77.26
77.28
77.30
chrX [Mbp]
outlier
no
yes
coverage
20
200
2000

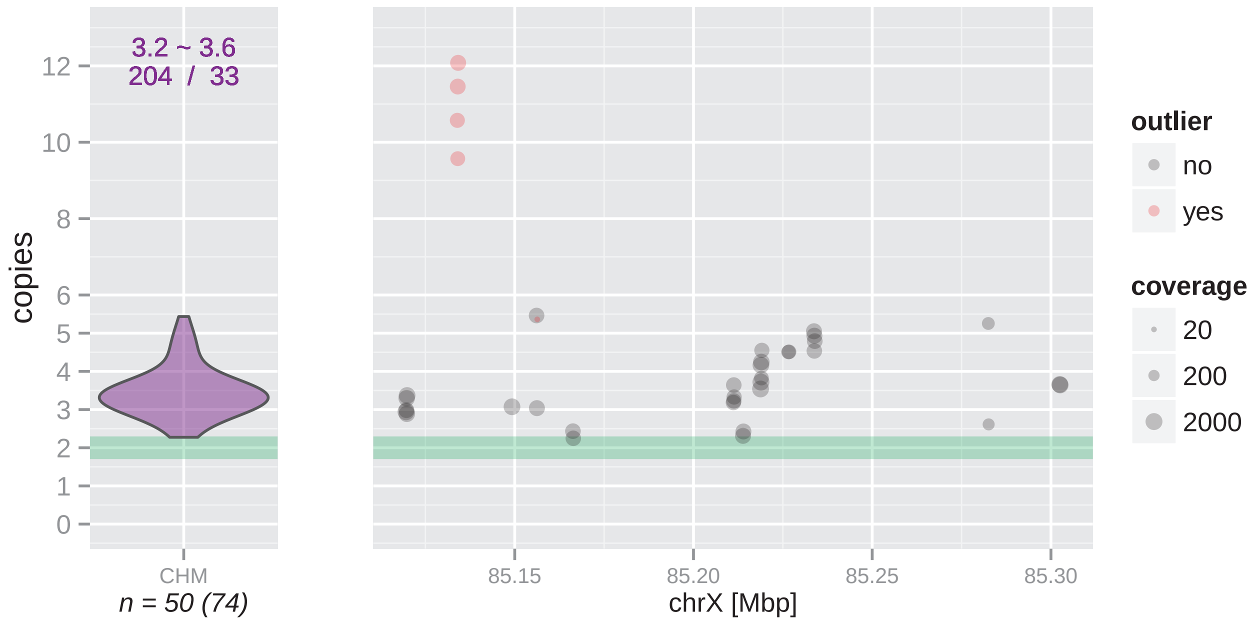
3.2 ~ 3.6
204 / 33
copies
12
10
8
6
5
4
3
2
1
0
CHM
n = 50 (74)
85.15
85.20
85.25
85.30
chrX [Mbp]
outlier
no
yes
coverage
20
200
2000

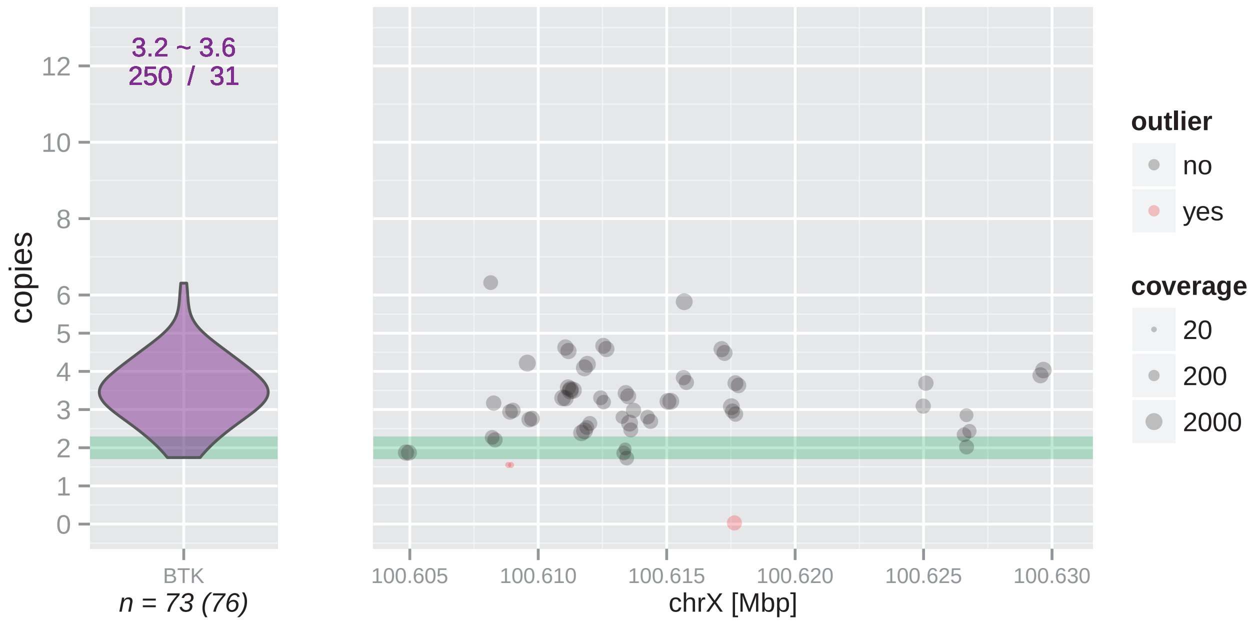
3.2 ~ 3.6
250 / 31
copies
12
10
8
6
5
4
3
2
1
0
BTK
n = 73 (76)
100.605
100.610
100.615
100.620
100.625
100.630
chrX [Mbp]
outlier
no
yes
coverage
20
200
2000
