# Supplementary figures and images for: Quantitative analysis of differences in copy numbers using read depth obtained from PCR-enriched samples and controls
Source: BMC Bioinformatics. 2015 Jan 28;16:17. doi: 10.1186/s12859-014-0428-5 (PMC4384318; doi:10.1186/s12859-014-0428-5)

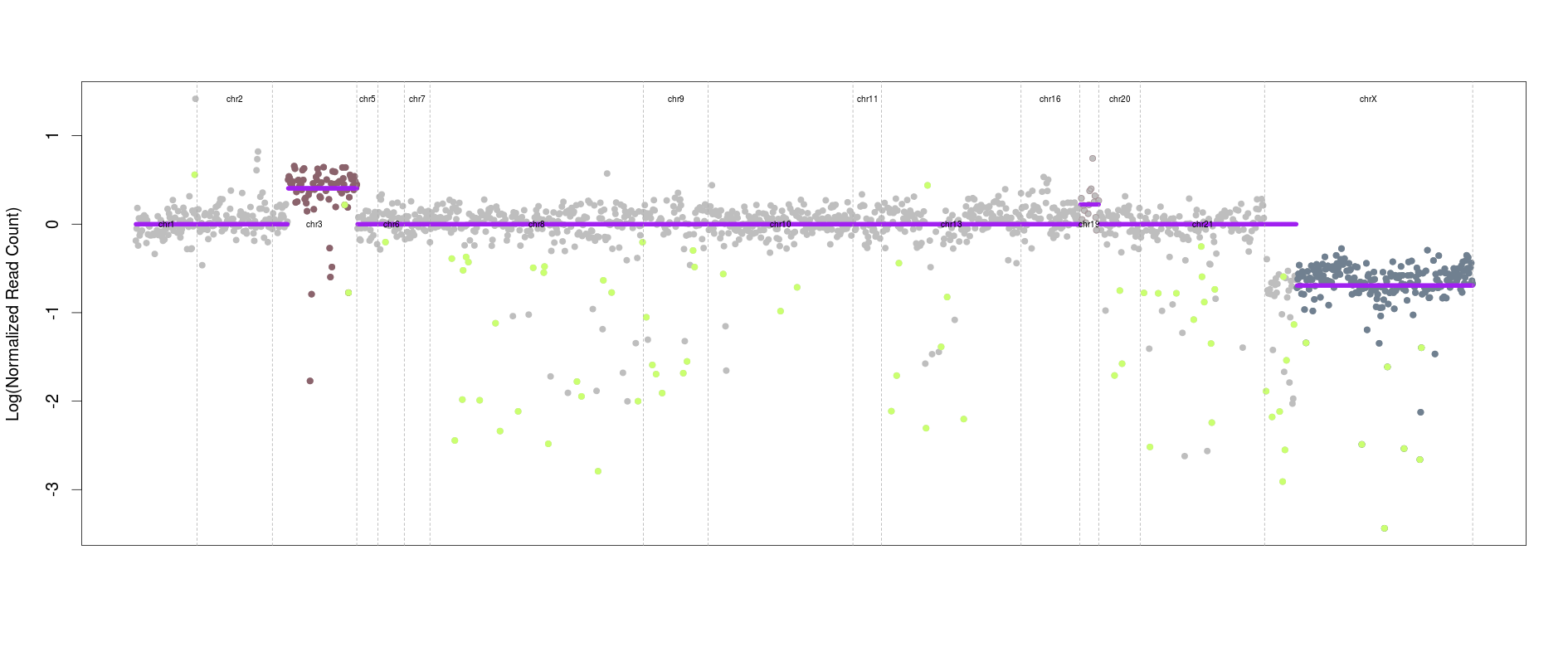

Supplement: Additional file 4 — Comparison with ONCOCNV. This archive (zip) contains all output-files generated for the comparison of quandico and ONCOCNV. [file 12859_2014_428_MOESM4_ESM.zip › ONCOCNV/M062_S10_NA20022.profile.png]

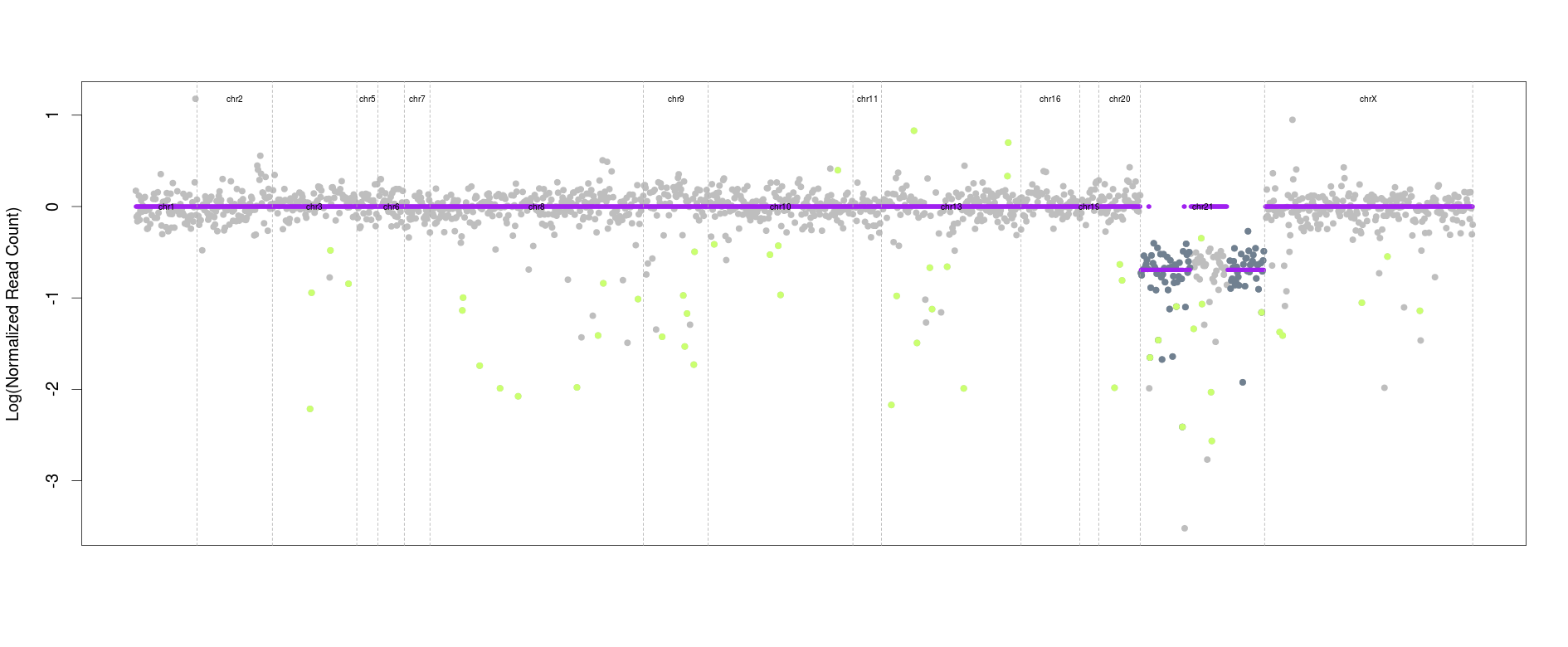

Supplement: Additional file 4 — Comparison with ONCOCNV. This archive (zip) contains all output-files generated for the comparison of quandico and ONCOCNV. [file 12859_2014_428_MOESM4_ESM.zip › ONCOCNV/M062_S1_NA01201.profile.png]

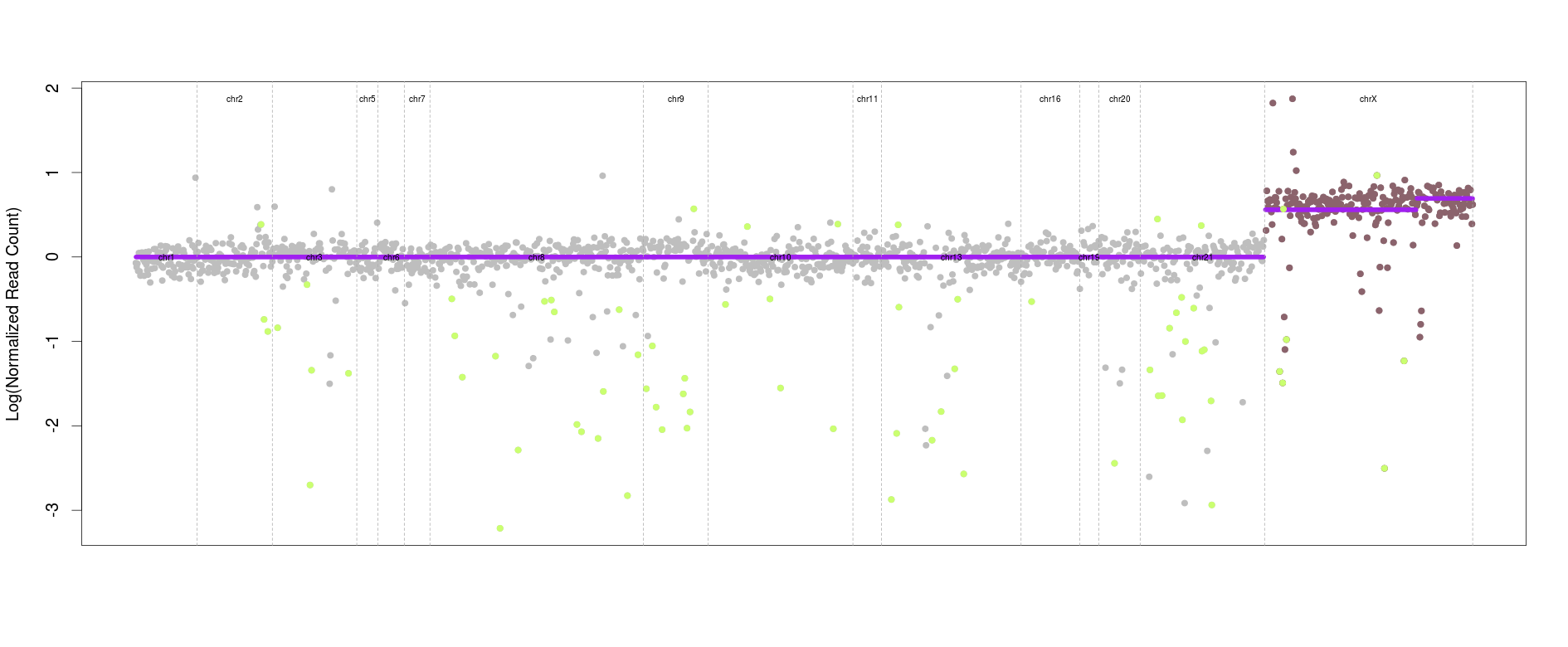

Supplement: Additional file 4 — Comparison with ONCOCNV. This archive (zip) contains all output-files generated for the comparison of quandico and ONCOCNV. [file 12859_2014_428_MOESM4_ESM.zip › ONCOCNV/M062_S2_NA01416.profile.png]

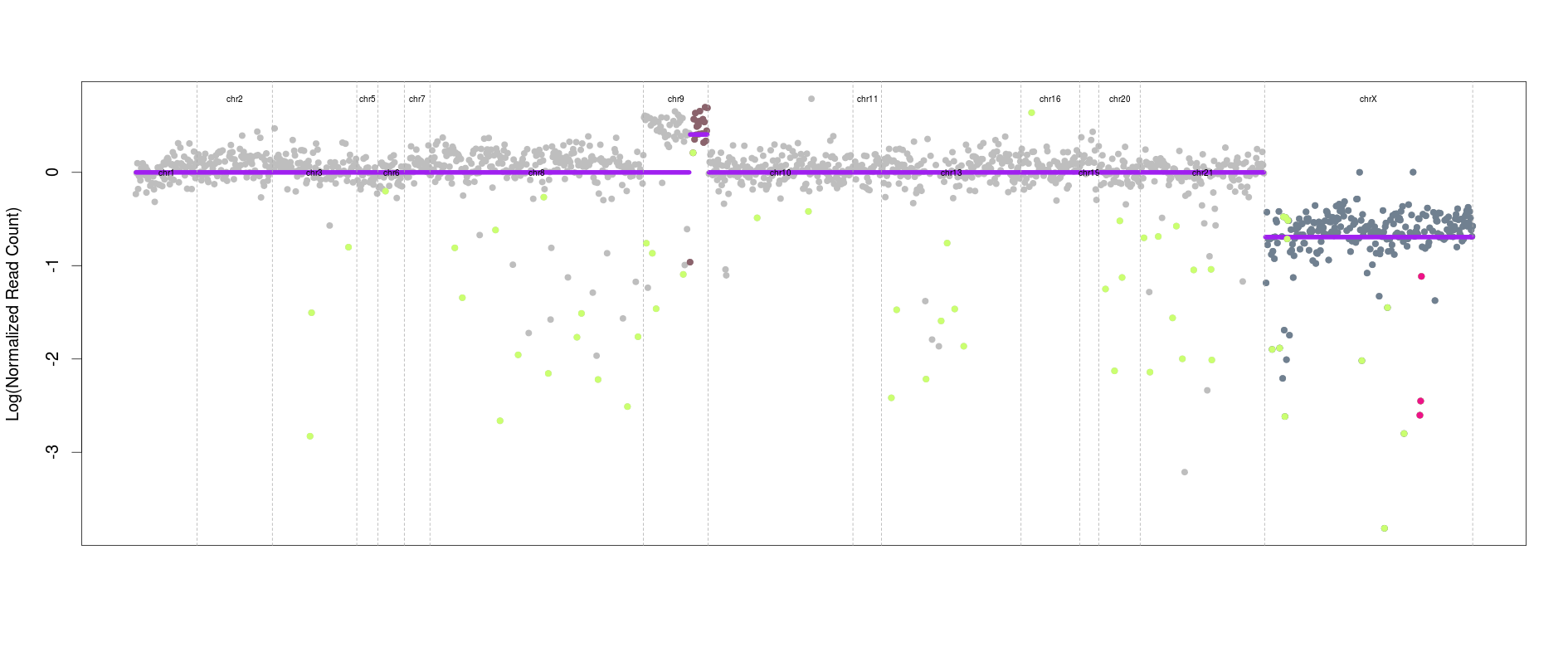

Supplement: Additional file 4 — Comparison with ONCOCNV. This archive (zip) contains all output-files generated for the comparison of quandico and ONCOCNV. [file 12859_2014_428_MOESM4_ESM.zip › ONCOCNV/M062_S3_NA05067.profile.png]

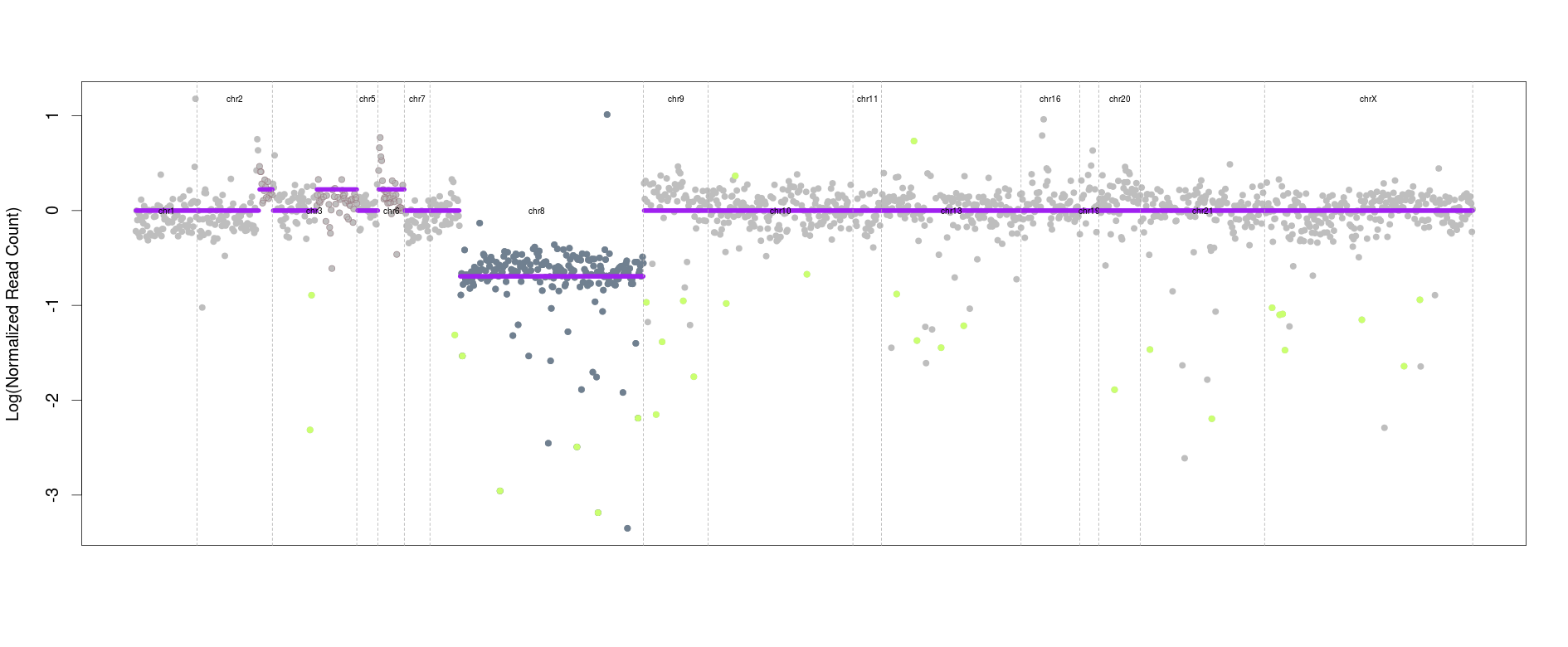

Supplement: Additional file 4 — Comparison with ONCOCNV. This archive (zip) contains all output-files generated for the comparison of quandico and ONCOCNV. [file 12859_2014_428_MOESM4_ESM.zip › ONCOCNV/M062_S4_NA09888.profile.png]

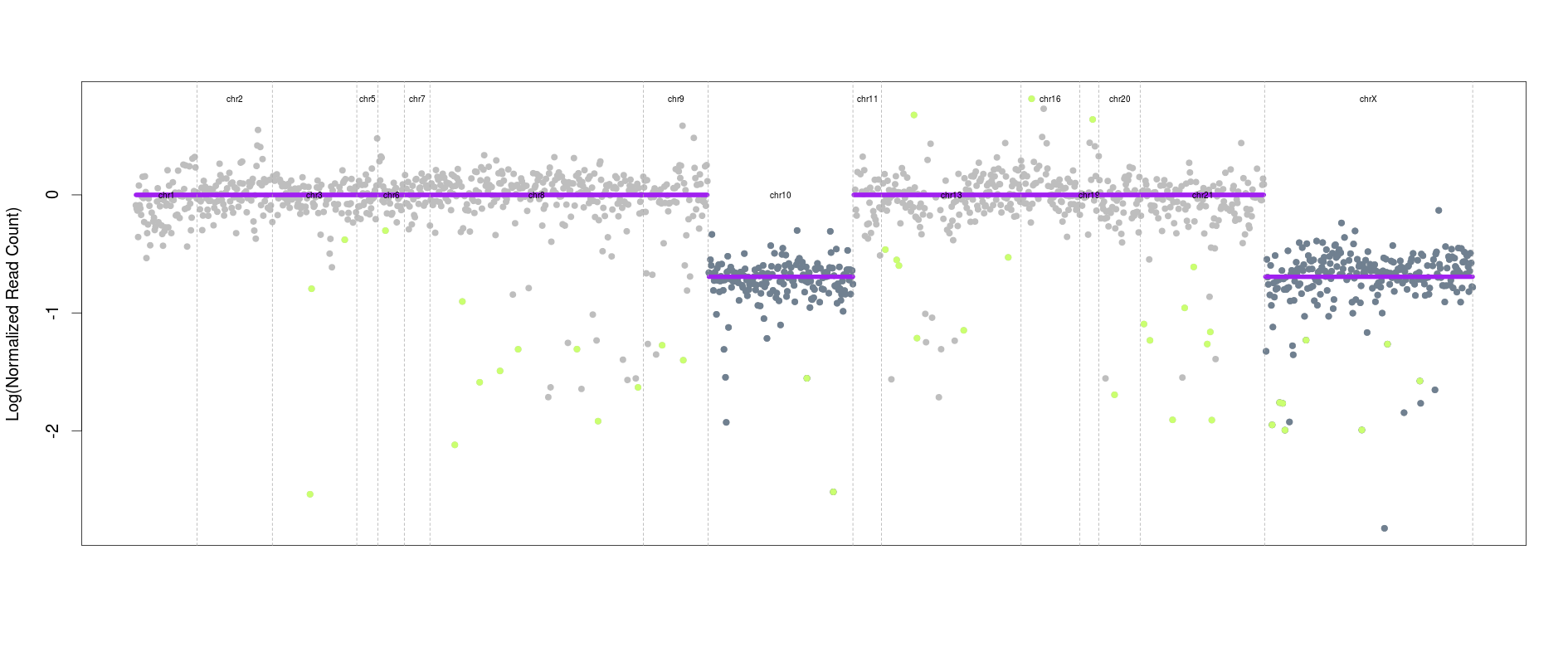

Supplement: Additional file 4 — Comparison with ONCOCNV. This archive (zip) contains all output-files generated for the comparison of quandico and ONCOCNV. [file 12859_2014_428_MOESM4_ESM.zip › ONCOCNV/M062_S5_NA11672.profile.png]

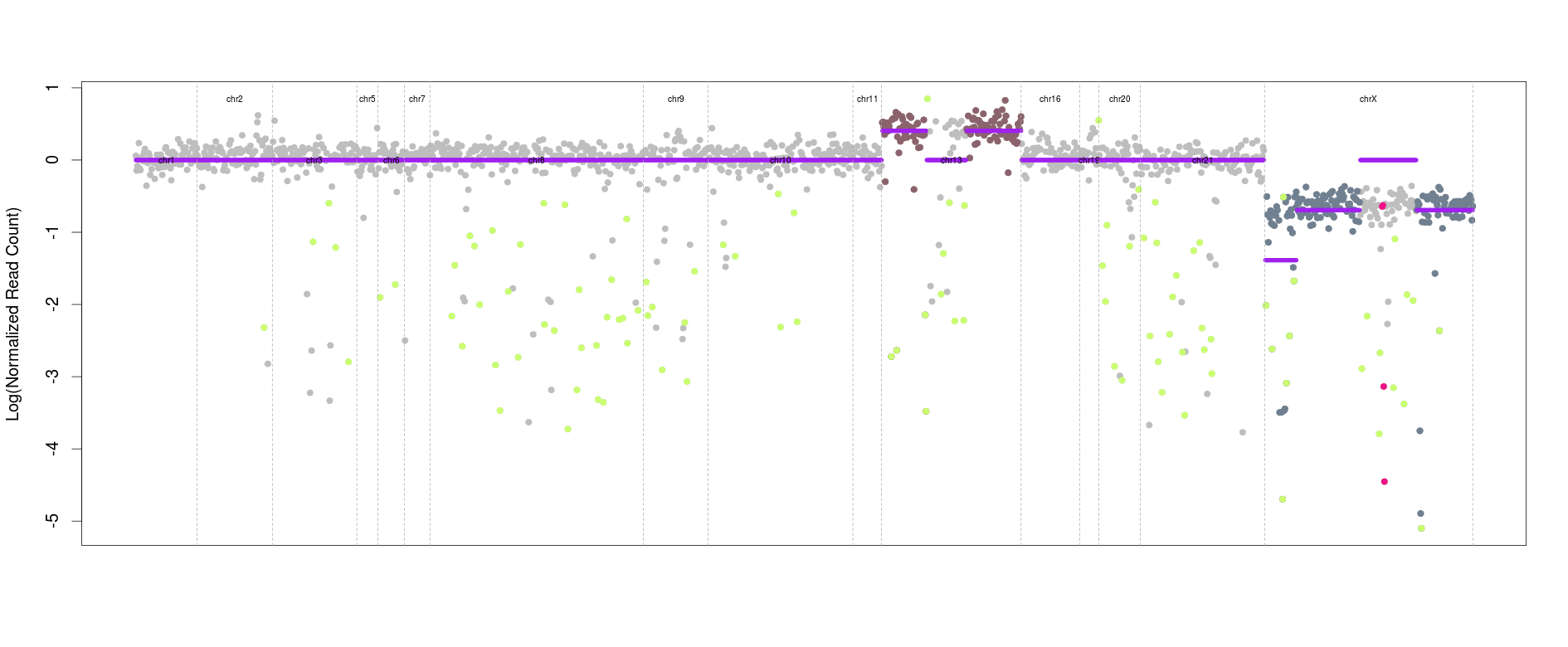

Supplement: Additional file 4 — Comparison with ONCOCNV. This archive (zip) contains all output-files generated for the comparison of quandico and ONCOCNV. [file 12859_2014_428_MOESM4_ESM.zip › ONCOCNV/M062_S6_NA12606.profile.png]

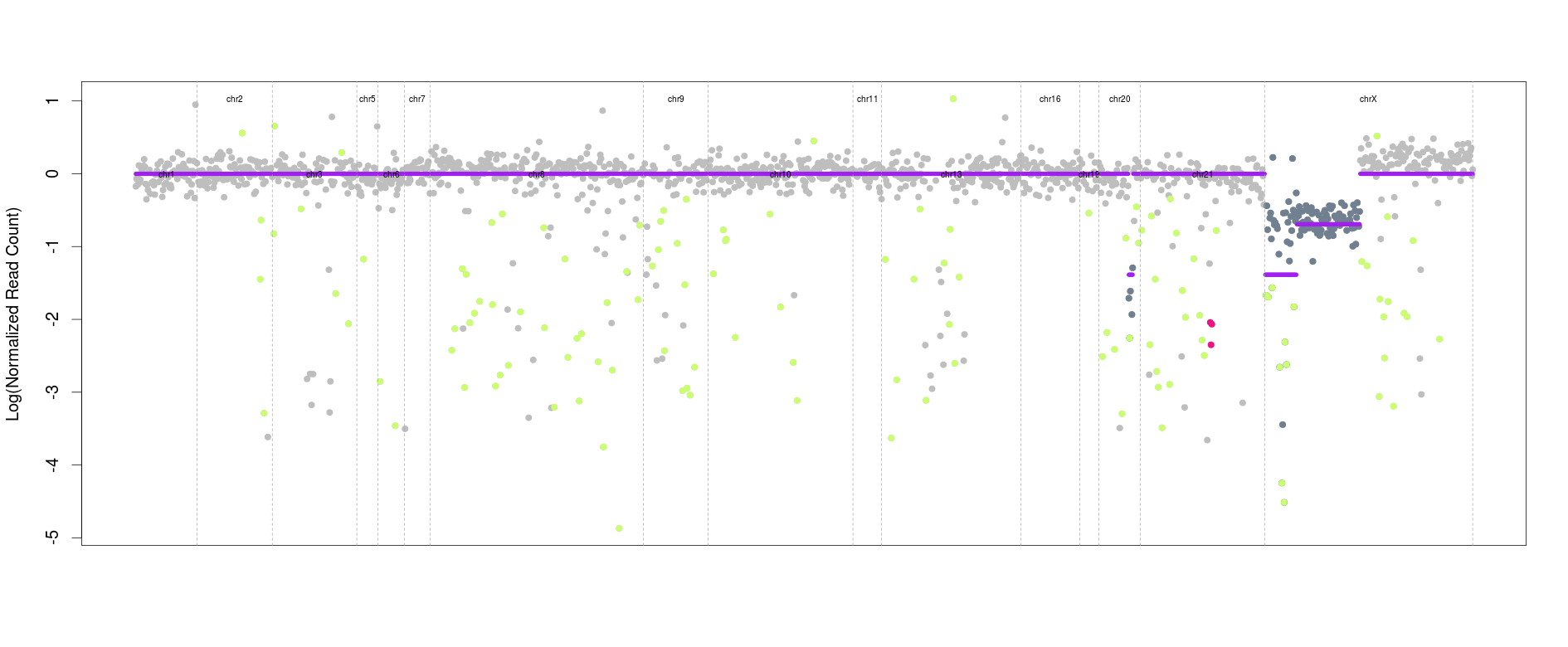

Supplement: Additional file 4 — Comparison with ONCOCNV. This archive (zip) contains all output-files generated for the comparison of quandico and ONCOCNV. [file 12859_2014_428_MOESM4_ESM.zip › ONCOCNV/M062_S7_NA13019.profile.png]

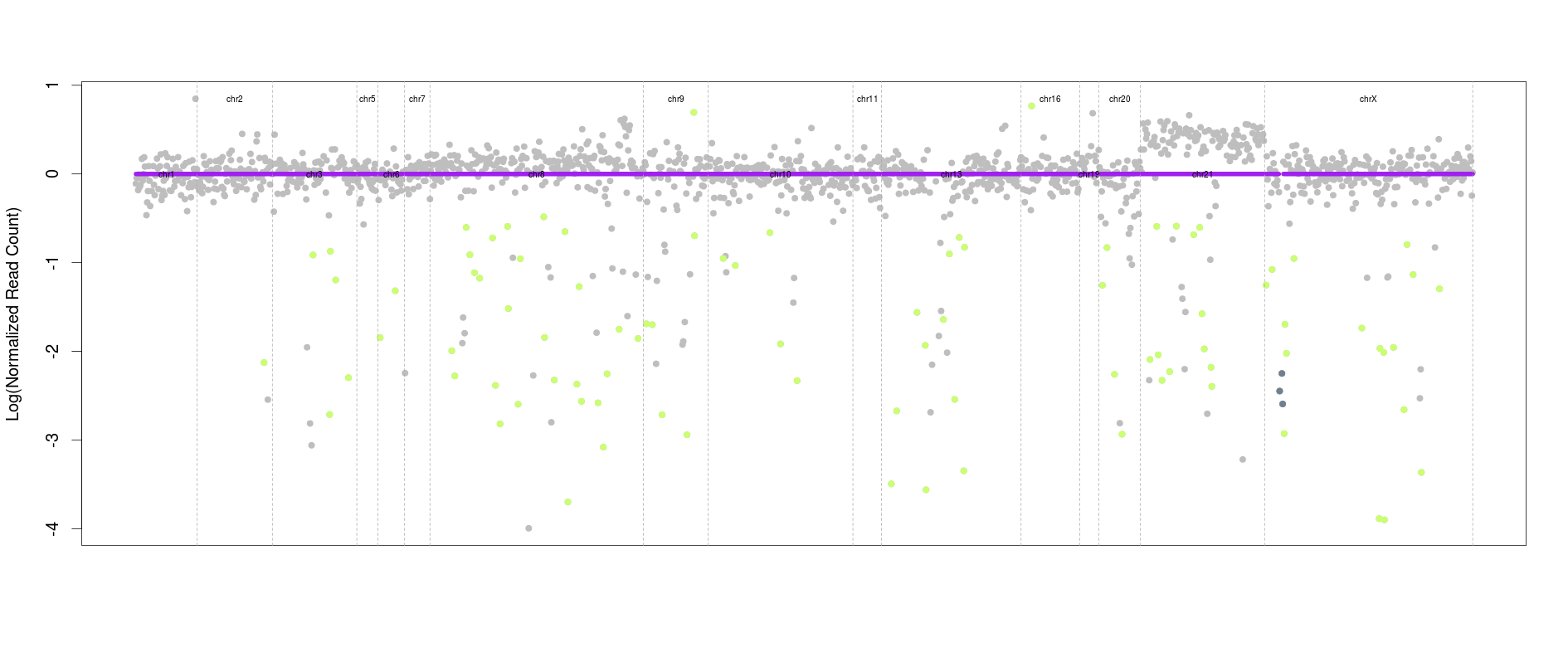

Supplement: Additional file 4 — Comparison with ONCOCNV. This archive (zip) contains all output-files generated for the comparison of quandico and ONCOCNV. [file 12859_2014_428_MOESM4_ESM.zip › ONCOCNV/M062_S8_NA13783.profile.png]

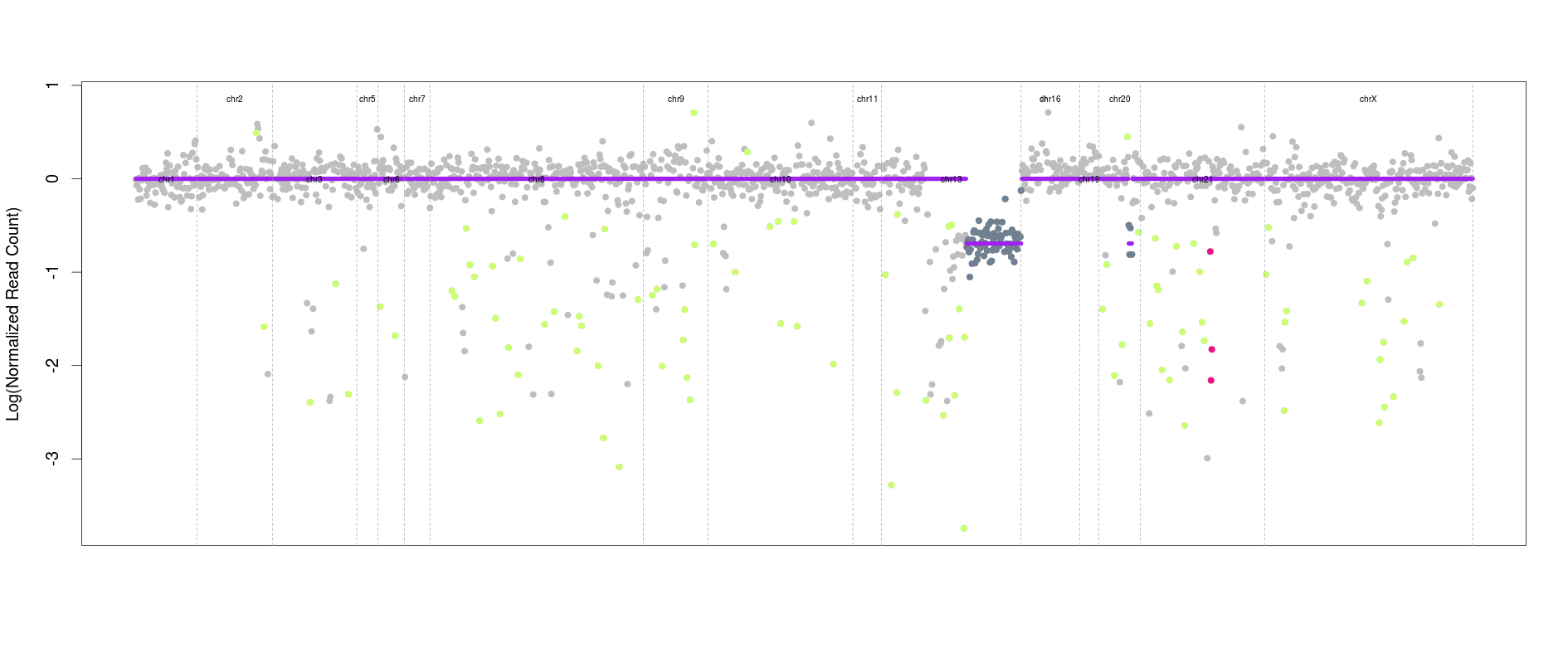

Supplement: Additional file 4 — Comparison with ONCOCNV. This archive (zip) contains all output-files generated for the comparison of quandico and ONCOCNV. [file 12859_2014_428_MOESM4_ESM.zip › ONCOCNV/M062_S9_NA14164.profile.png]

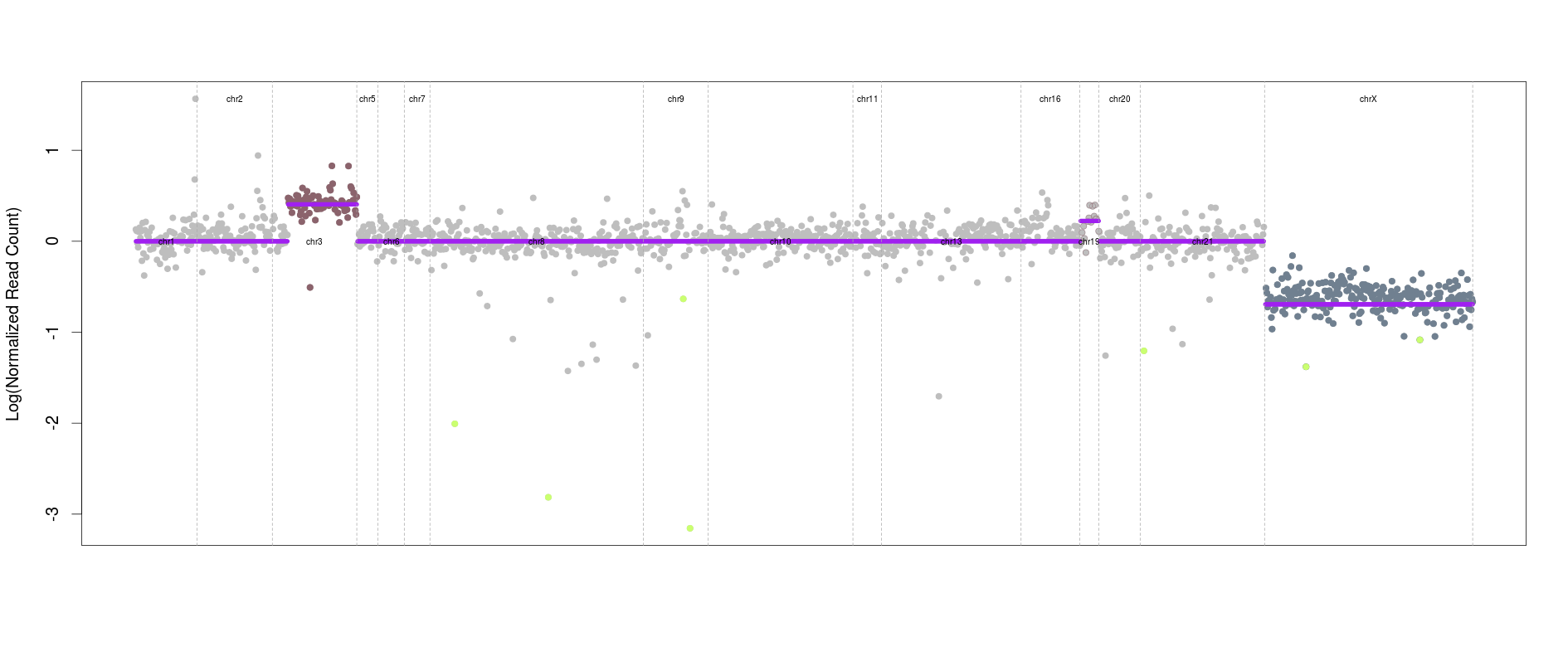

Supplement: Additional file 4 — Comparison with ONCOCNV. This archive (zip) contains all output-files generated for the comparison of quandico and ONCOCNV. [file 12859_2014_428_MOESM4_ESM.zip › ONCOCNV/M063_S10_NA20022.profile.png]

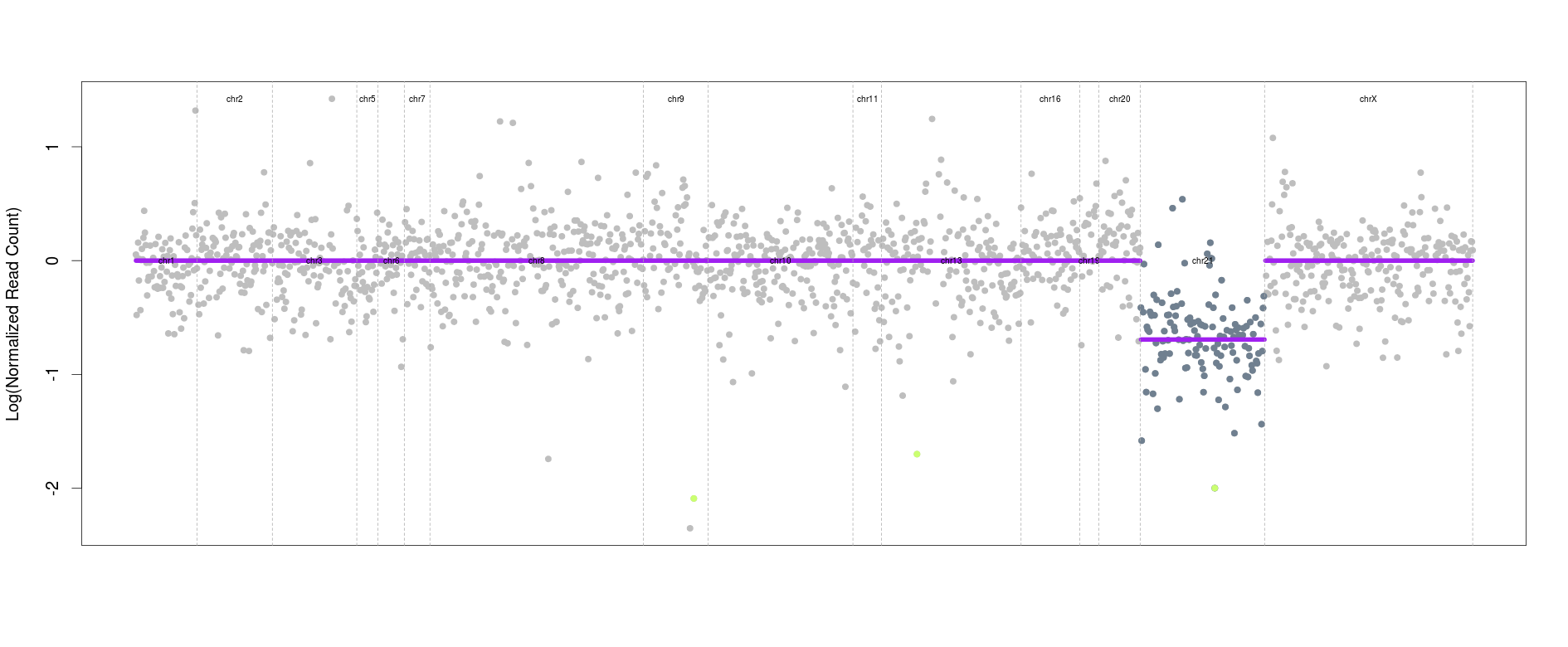

Supplement: Additional file 4 — Comparison with ONCOCNV. This archive (zip) contains all output-files generated for the comparison of quandico and ONCOCNV. [file 12859_2014_428_MOESM4_ESM.zip › ONCOCNV/M063_S1_NA01201.profile.png]

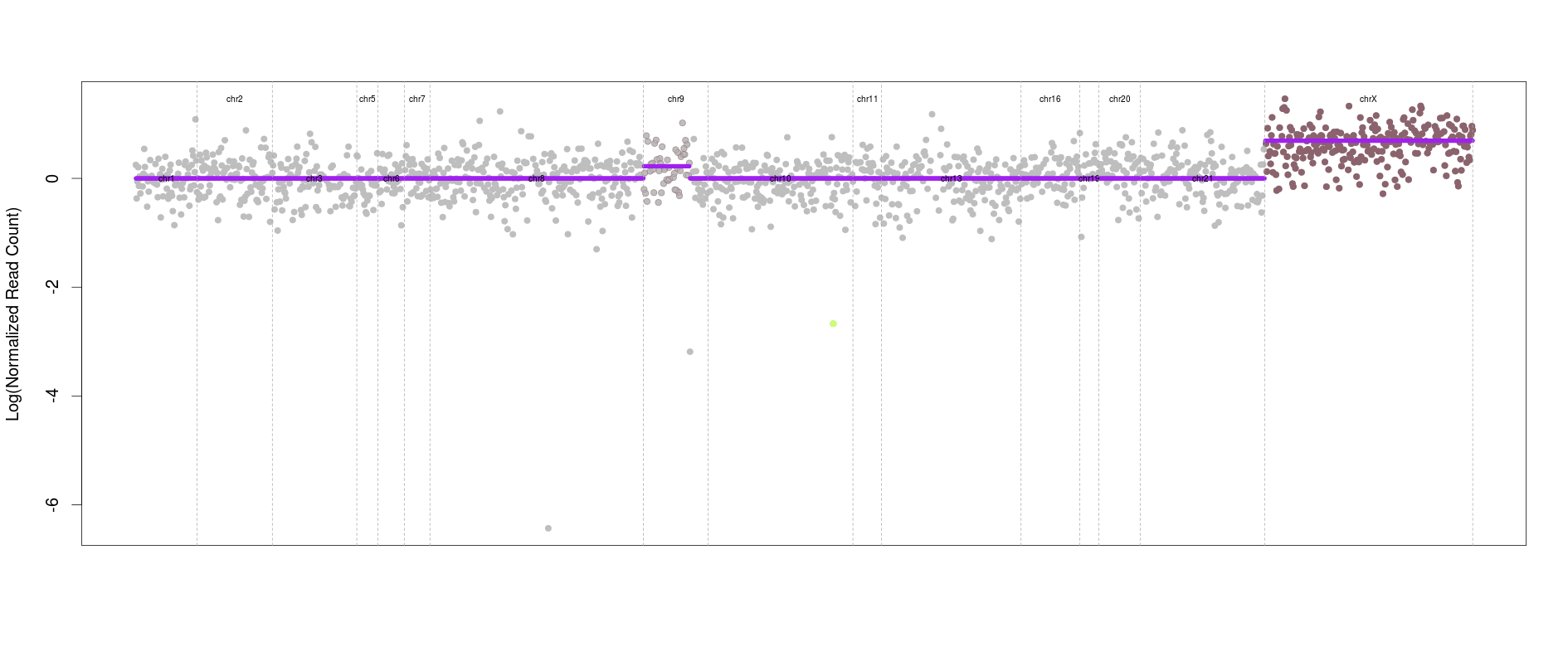

Supplement: Additional file 4 — Comparison with ONCOCNV. This archive (zip) contains all output-files generated for the comparison of quandico and ONCOCNV. [file 12859_2014_428_MOESM4_ESM.zip › ONCOCNV/M063_S2_NA01416.profile.png]

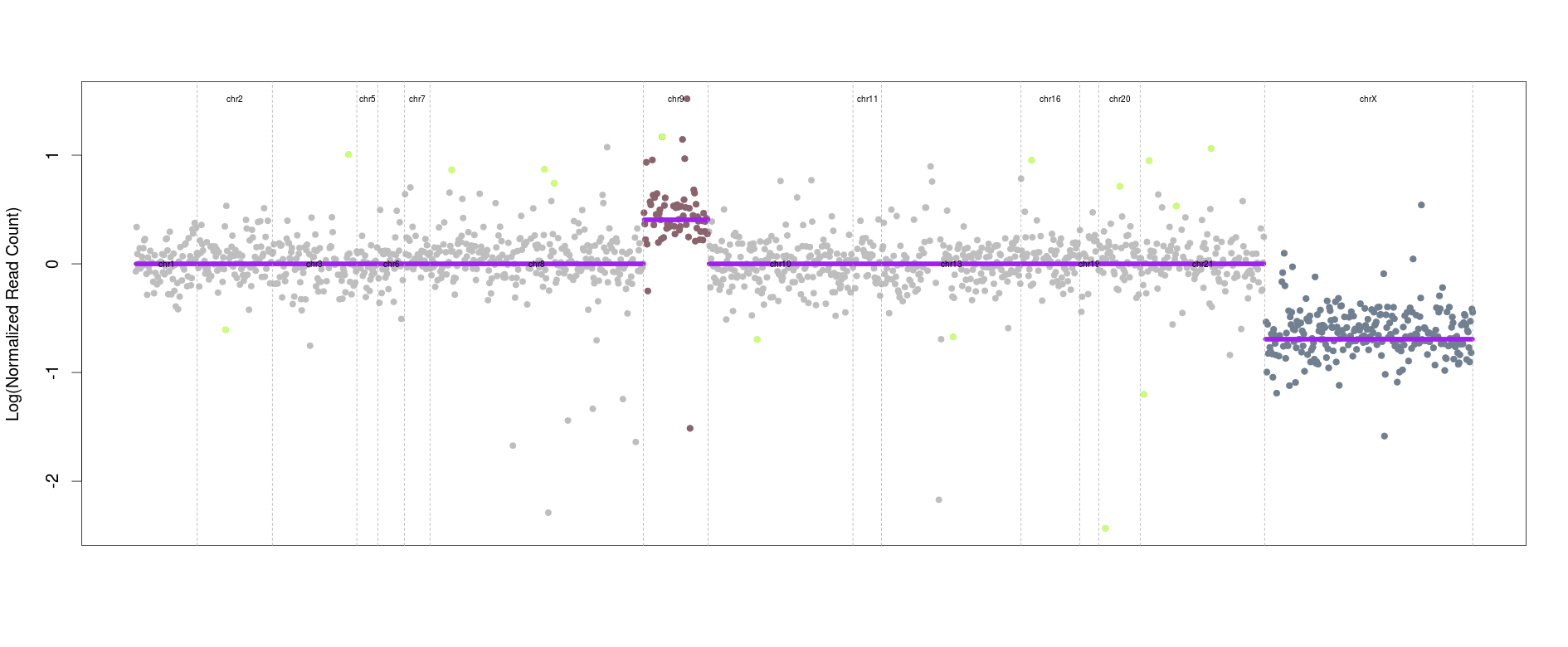

Supplement: Additional file 4 — Comparison with ONCOCNV. This archive (zip) contains all output-files generated for the comparison of quandico and ONCOCNV. [file 12859_2014_428_MOESM4_ESM.zip › ONCOCNV/M063_S3_NA05067.profile.png]

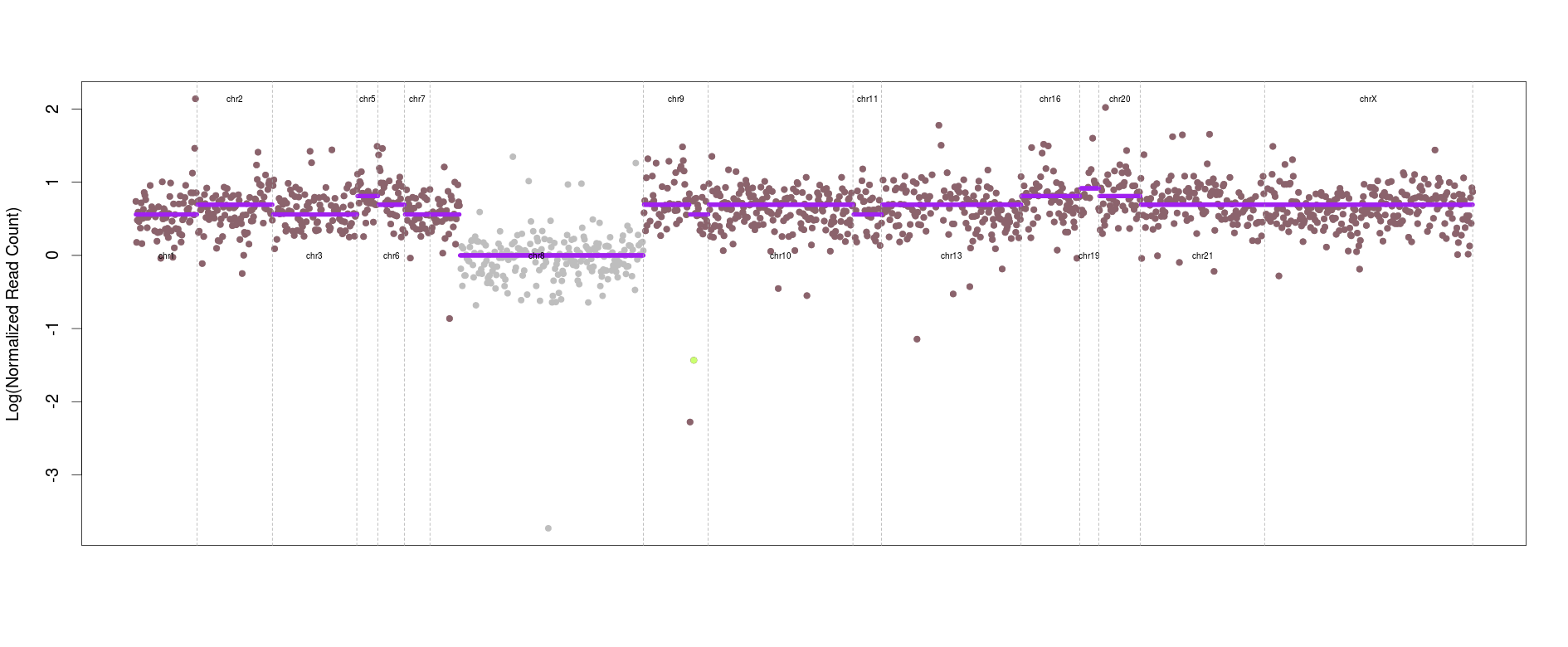

Supplement: Additional file 4 — Comparison with ONCOCNV. This archive (zip) contains all output-files generated for the comparison of quandico and ONCOCNV. [file 12859_2014_428_MOESM4_ESM.zip › ONCOCNV/M063_S4_NA09888.profile.png]

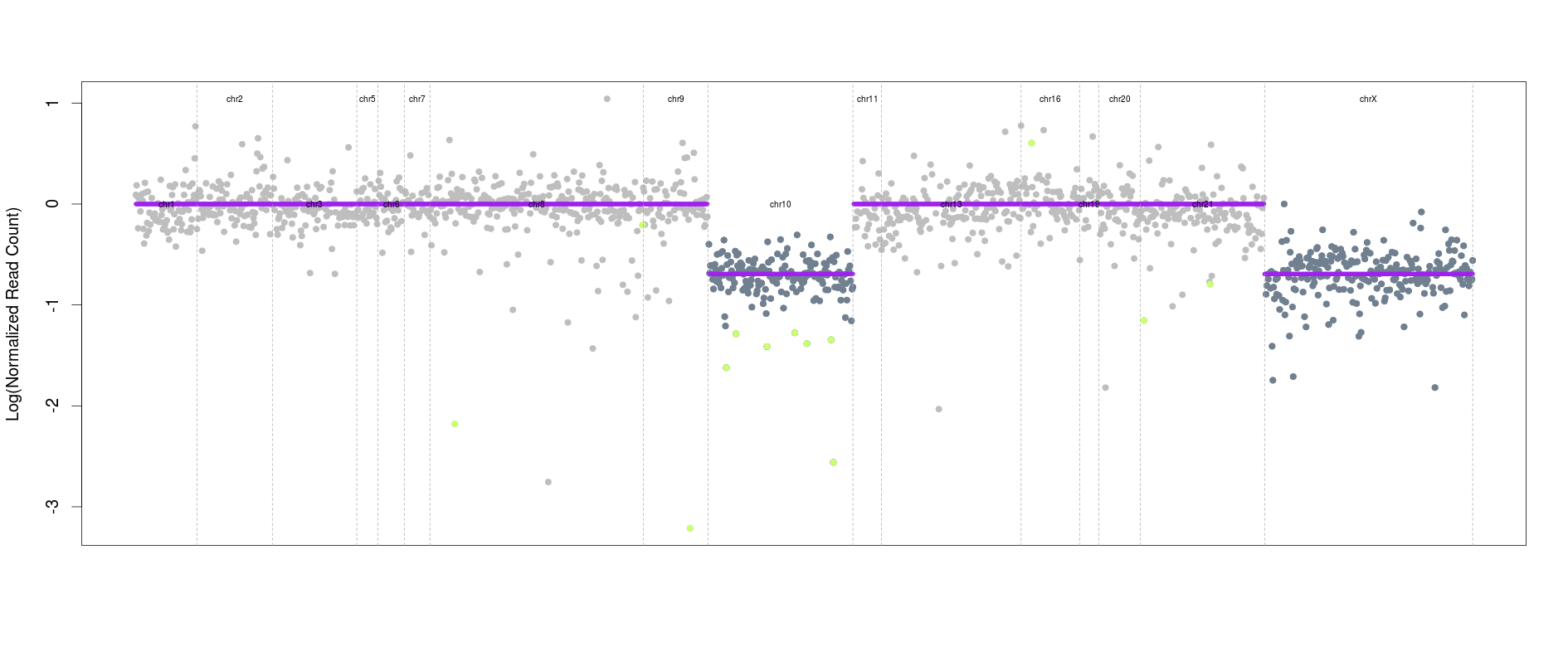

Supplement: Additional file 4 — Comparison with ONCOCNV. This archive (zip) contains all output-files generated for the comparison of quandico and ONCOCNV. [file 12859_2014_428_MOESM4_ESM.zip › ONCOCNV/M063_S5_NA11672.profile.png]

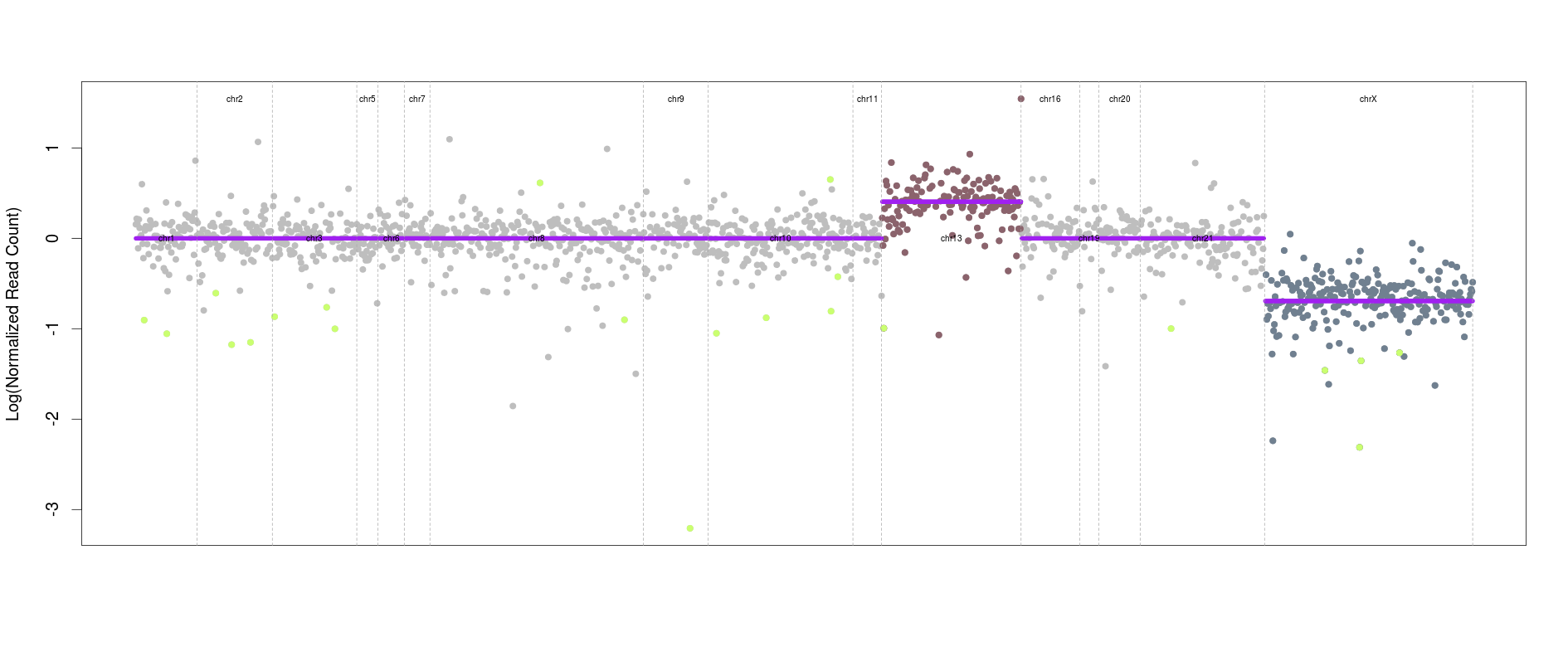

Supplement: Additional file 4 — Comparison with ONCOCNV. This archive (zip) contains all output-files generated for the comparison of quandico and ONCOCNV. [file 12859_2014_428_MOESM4_ESM.zip › ONCOCNV/M063_S6_NA12606.profile.png]

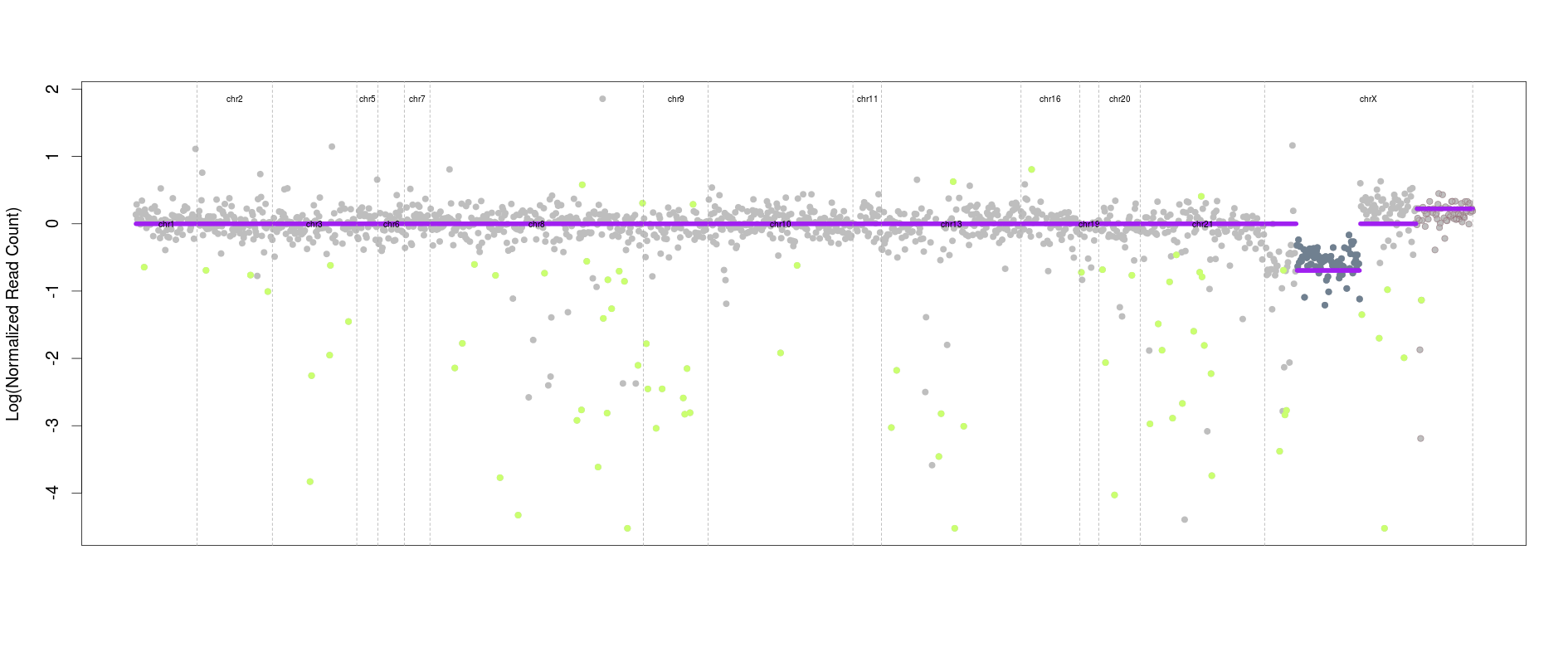

Supplement: Additional file 4 — Comparison with ONCOCNV. This archive (zip) contains all output-files generated for the comparison of quandico and ONCOCNV. [file 12859_2014_428_MOESM4_ESM.zip › ONCOCNV/M063_S7_NA13019.profile.png]

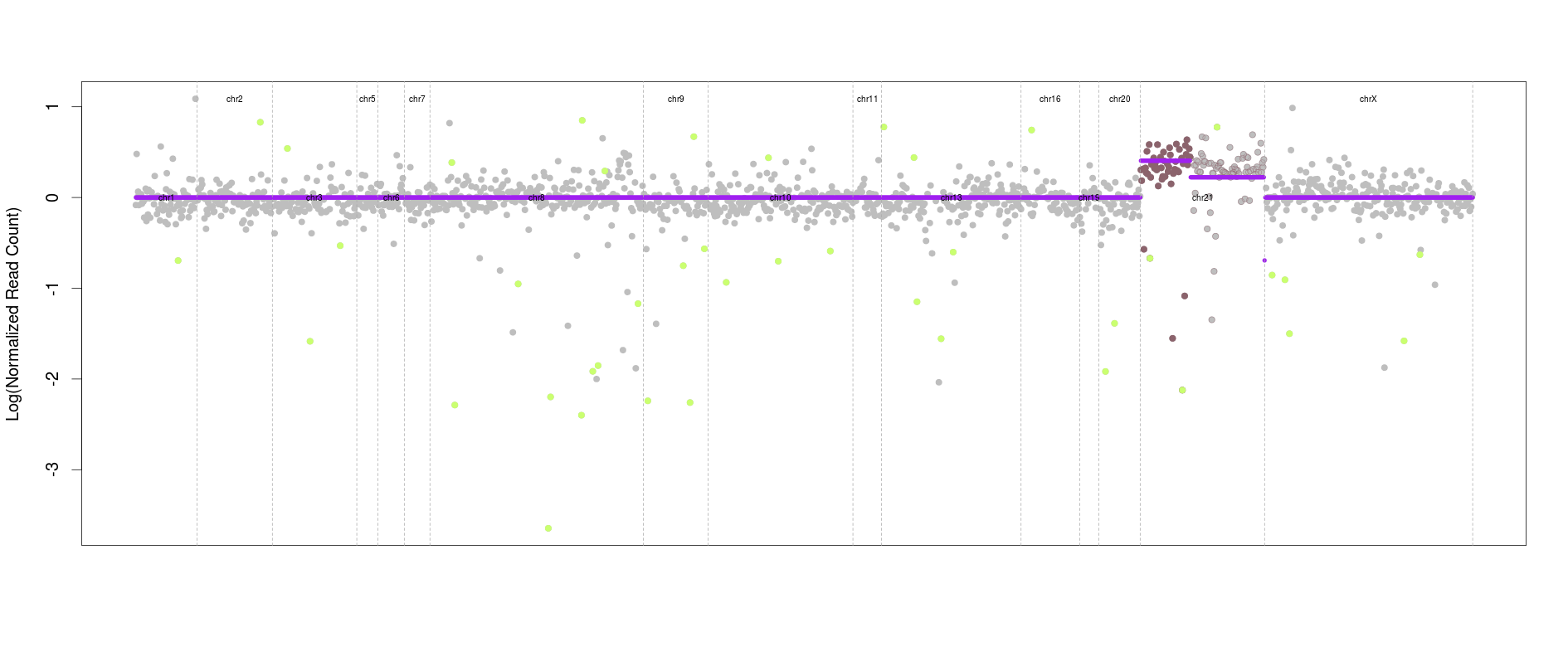

Supplement: Additional file 4 — Comparison with ONCOCNV. This archive (zip) contains all output-files generated for the comparison of quandico and ONCOCNV. [file 12859_2014_428_MOESM4_ESM.zip › ONCOCNV/M063_S8_NA13783.profile.png]

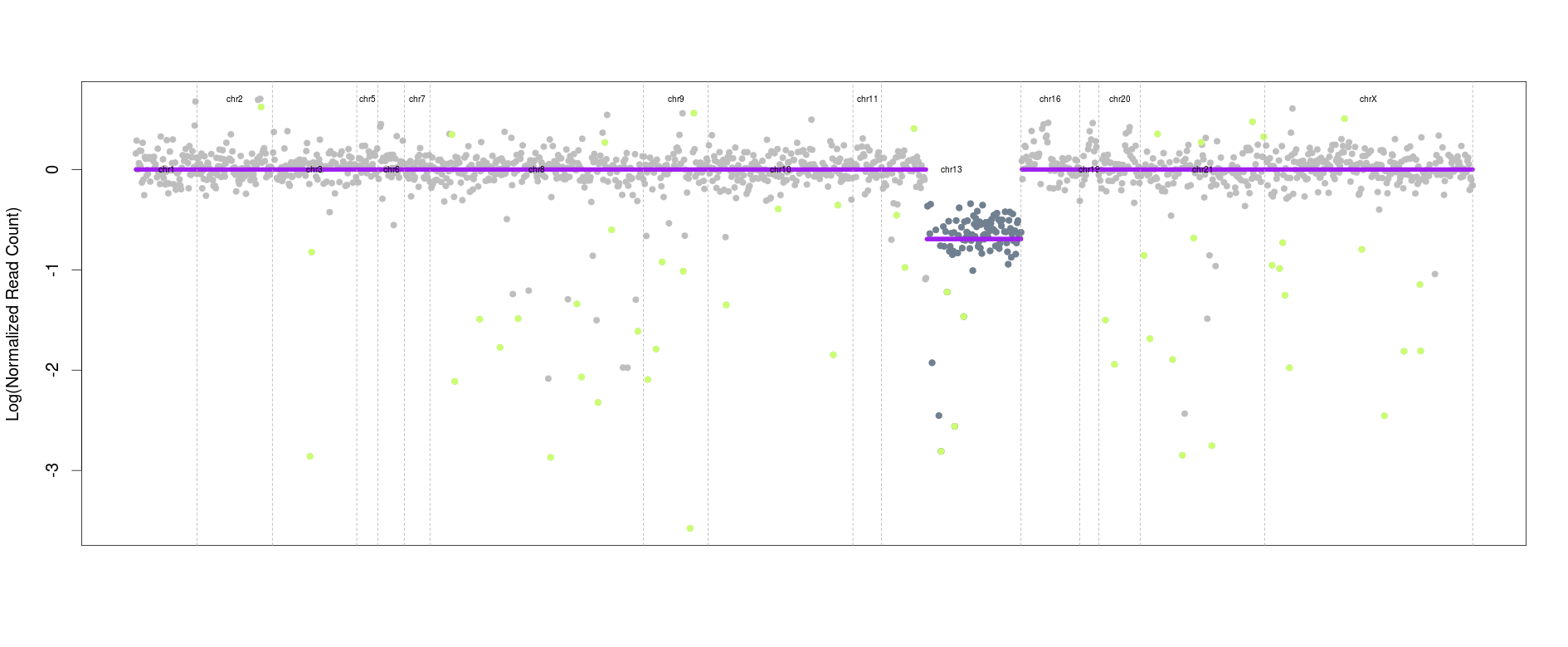

Supplement: Additional file 4 — Comparison with ONCOCNV. This archive (zip) contains all output-files generated for the comparison of quandico and ONCOCNV. [file 12859_2014_428_MOESM4_ESM.zip › ONCOCNV/M063_S9_NA14164.profile.png]

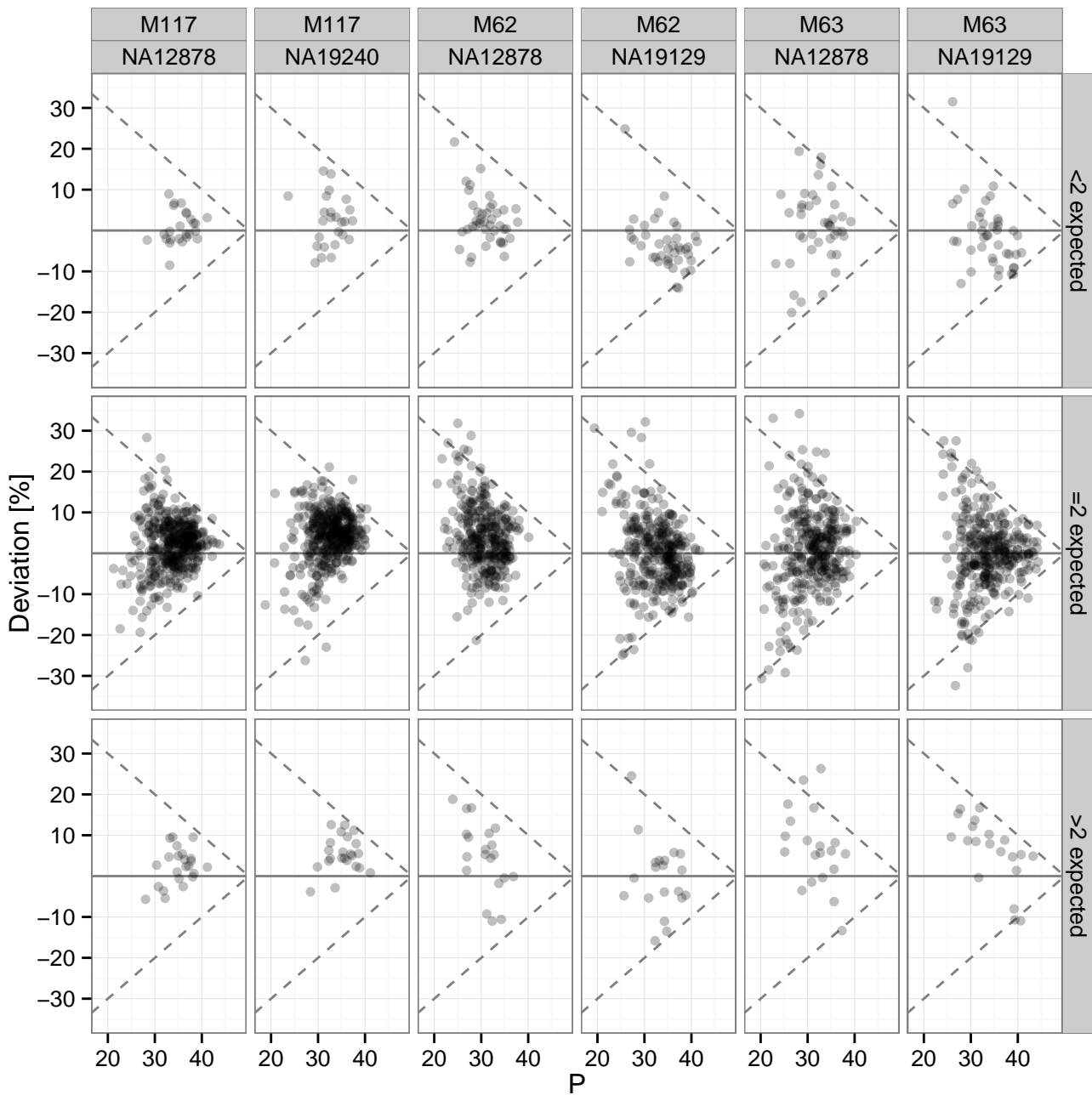

Supplement: Additional file 5 — Correlation of expected and estimated copy number. Graph (pdf) of the score P (derived from the observed standard error, see equation 9) plotted against the actual precision of the copy number assignment. Deviation is calculated as (N called−N expected)/N called. The dashed lines correspond to a linear increase of precision (1 percentage point per increase in score). Different expected copy numbers are shown in three rows (top: <2, center: =2, bottom >2). [file 12859_2014_428_MOESM5_ESM.pdf]

# CNA902Y (M62, M63)

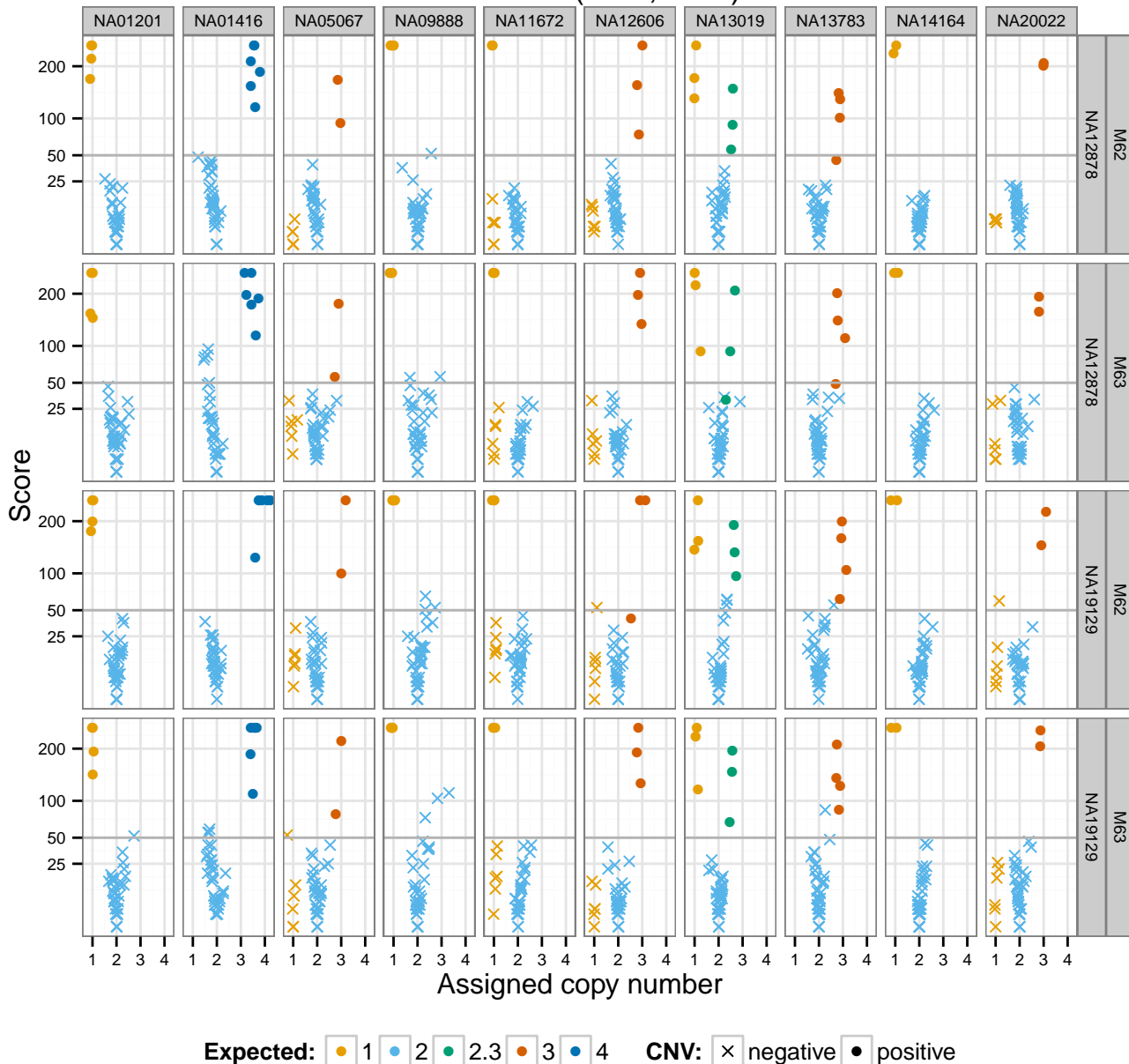

# NGHS-991Y (M117)

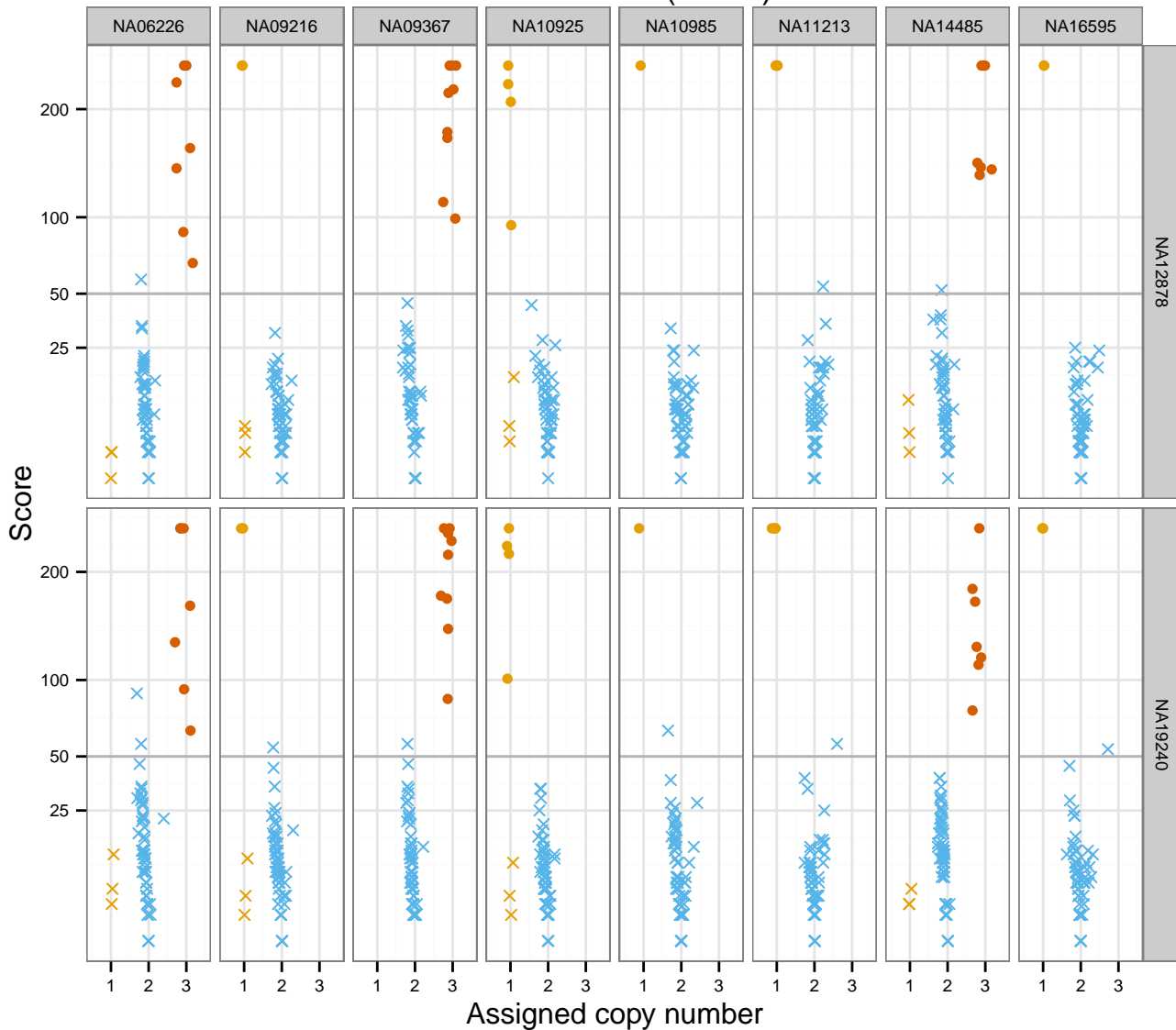

**CNV:** × negative • positive **Expected:** ● 1 ● 2 ● 3

Supplement: Additional file 6 — Summary of all calls. Graphical representation (pdf) of all copy number calls done with the final algorithm. Page 1: CNA902Y with 1600 calls (40 clusters, ten samples, two controls, two runs). Each analysis is based on a comparison of a samples (column) with a control (row) of a certain sequencing run (row). Page 2: Set NGHS-991Y with 960 calls (60 clusters, eight samples, two controls). Regions with no expected CNV are shown as crosses (×) while expected differences are shown as circles (∙). The expected copy numbers are depicted by different colors. [file 12859_2014_428_MOESM6_ESM.pdf]
